# Synthesis and Structure–Activity Relationship Studies of Benzimidazole-4,7-dione-Based P2X3 Receptor Antagonists as Novel Anti-Nociceptive Agents

**DOI:** 10.3390/molecules27041337

**Published:** 2022-02-16

**Authors:** Jinsu Bae, Yeo-Ok Kim, Xuehao Han, Myung-Ha Yoon, Woong-Mo Kim, Yong-Chul Kim

**Affiliations:** 1 Department of Biomedical Science and Engineering, Gwangju Institute of Science and Technology (GIST), Gwangju 61005, Korea; ekdlwj@gmail.com; 2 Department of Anesthesiology and Pain Medicine, Chonnam National University Medical School, Gwangju 61469, Korea; yeok1017@jnu.ac.kr (Y.-O.K.); 178079@jnu.ac.kr (X.H.); mhyoon@jnu.ac.kr (M.-H.Y.); 3 School of Life Sciences, Gwangju Institute of Science and Technology (GIST), Gwangju 61005, Korea

**Keywords:** neuropathic pain, anti-nociceptive agents, P2X3 receptor, adenosine 5′-triphosphate, antagonist, structure−activity relationship study

## Abstract

P2X3 receptors (P2X3R) are ATP-gated ion channels predominantly expressed in C- and Aδ-fiber primary afferent neurons and have been introduced as a novel therapeutic target for neurological disorders, including neuropathic pain and chronic cough. Because of its localized distribution, antagonism of P2X3R has been thoroughly considered, and the avoidance of issues related to CNS side effects has been proven in clinical trials. In this article, benzimidazole-4,7-dione-based derivatives were introduced as a new chemical entity for the development of P2X3R antagonists. Starting from the discovery of a hit compound from the screening of 8364 random library compounds in the Korea Chemical Bank, which had an IC_50_ value of 1030 nM, studies of structure–activity and structure–property relationships enabled further optimization toward improving the antagonistic activities as well as the drug’s physicochemical properties, including metabolic stability. As for the results, the final optimized compound **14h** was developed with an IC_50_ value of 375 nM at P2X3R with more than 23-fold selectivity versus P2X2/3R, along with properties of metabolic stability and improved solubility. In neuropathic pain animal models evoked by either nerve ligation or chemotherapeutics in male Sprague-Dawley rats, compound **14h** showed anti-nociceptive effects through an increase in the mechanical withdrawal threshold as measured by von Frey filament following intravenous administration.

## 1. Introduction

P2X receptors (P2XR) are reported to function as ligand-gated cation channels that are activated by the binding of extracellular adenosine 5′-triphosphate (ATP) [1,2] to induce the influx of cations, e.g., calcium, potassium, and sodium ions, within milliseconds, which consequently triggers neuronal responses via the depolarization of cell membranes [3,4,5]. Seven subtypes of P2X receptors have been identified in mammalian cells and are widely expressed in peripheral and central nerve systems, vascular smooth muscles, platelets, and immune cells, in which various physiological processes are involved with the receptors, including synaptic transmission, presynaptic modulation, smooth muscle contraction, cell proliferation and death, intestinal motility, platelet aggregation, taste, nociception, and inflammation [6,7]. Among the P2X receptor subtypes, P2X3 receptors (P2X3R) are expressed as homomeric P2X3 or heteromeric P2X2/3 trimers predominantly in small-to-medium-diameter C- and Aδ-fibers of primary afferent neurons, which suggests that these receptors could serve as therapeutic targets that are highly specific to the pain sensing systems and thus allow the avoidance of CNS-related side effects [8,9].

**Figure 1:**
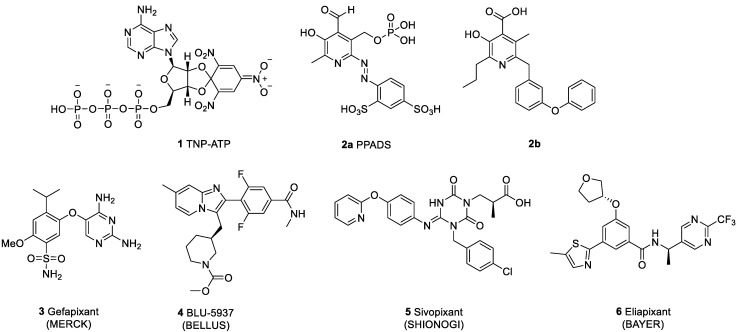
Structures of various hP2X3 receptor antagonists.

Further studies on P2X3 receptors have validated their relationship with pain sensation, including the experimental results of the nociceptive behaviors by the injection of ATP or α,β-methylene ATP, P2X3 receptor agonists, into the hind paws of rats and the pain relief effects with attenuated tactile allodynia and mechanical hyperalgesia in P2X3 receptor knockout or knockdown animals [10,11,12]. In addition, the administration of P2X3 receptor antagonists, e.g., TNP-ATP (**1**: 2′,3′-O-(2,4,6-trinitrophenyl)adenosine-5′-triphosphate) and PPADS (**2a**: pyridoxalphosphate-6-azophenyl-2′,4′-disulfonate), resulted in decreases in dorsal root ganglion neuron responses and neuropathic pain (Figure 1) [13].

For decades, potent and selective P2X3 receptor antagonists have been developed by pharmaceutical industries and academic institutes. In our previous research, extensive structure–activity relationship (SAR) studies were performed to optimize the potency and selectivity of the nonselective P2XR antagonist, PPADS (**2a**), resulting in the discovery of a novel selective P2X3R antagonist (**2b**) with potent anti-nociceptive effects in the nerve ligation and cisplatin-induced pain animal models (Figure 1) [14]. As the first-in-class drug, gefapixant (**3**, MK-7264 or AF-219), a diaminopyridine derivative, was approved by the FDA for the treatment of chronic cough, but not neuropathic pain, in 2021 [15]. However, the loss of taste as a side effect of gefapixant due to the low selectivity between P2X3 vs. P2X2/3 receptors has been considered as an important unmet need [16]. Thus, several pharmaceutical industries, e.g., Bellus, Shionogi, and Bayer, have developed antagonists that are more selective for P2X3 receptor over P2X2/3 receptors, such as BLU-5937 (**4**), Sivopixant (**5**), and Eliapixant (**6**). Those derivatives have been investigated in clinical trials for chronic cough treatment.

In this article, our group reported a discovery of benzimidazole-4,7-dione-based novel hP2X3R antagonists with anti-nociceptive activities in pain animal models derived from library screening, hit to lead optimization with SAR, in vitro and in vivo pharmacokinetics, and in vivo animal studies.

## 2. Results and Discussions

### 2.1. Library Screening: Discovery of New Scaffold for hP2X3 Receptor Antagonist

**Figure 2:**
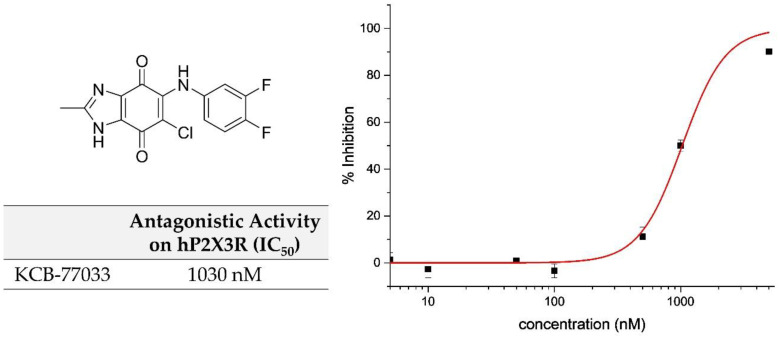
The hit compound identified from the library screening of 8364 compounds.

For the evaluation of P2XR activities, a cell-based assay system was employed to measure the level of fluorescence emission, based on Fluo-4 dye, during Ca^2+^ influx evoked by αβmeATP in HEK293 cells stably expressing hP2X2/3R and hP2X3R. The library containing 8364 representative compounds for the screening of antagonistic activity was provided by the Korea Chemical Bank. The antagonistic activities were evaluated in the presence of P2X3R agonist of the concentration for EC_70_ value (200–250 nM for hP2X3R and 1–4 μM for hP2X2/3R highly expressed cell lines), and AF353 was used as the positive control antagonist for comparison. For the first round of screening, a single concentration (25 μM) of each library compound was tested to identify initial hit compounds, which were subsequently validated using both concentrations of 25 and 10 μM. Using a cut-off threshold of higher than 50% antagonistic activity and after confirming dose-dependency, 12 main hit compounds were selected. Among them, a benzimidazole-4,7-dione analogue (**KCB-77033**, Figure 2) with an IC_50_ value of 1030 nM was finally discovered as the hit compound satisfying several factors such as having good antagonistic activity, a novel scaffold as a P2X3R antagonist, and affordability for studying SAR after further screening of benzimidazole-4,7-dione-related derivatives from the Korea Chemical Bank.

### 2.2. General Synthesis for Benzimidazole-4,7-dione Derivatives

**Scheme 1:**
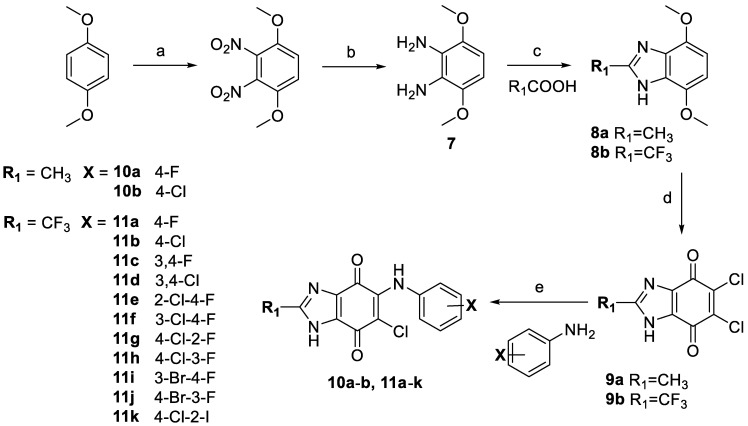
Scheme for synthesis of the benzimidazole-4,7-dione arylamine derivatives under the following conditions: (**a**) conc. HNO_3_, ice bath 1 h, rt 1 h, 90 °C 1 h, 91%; (**b**) Pd/C, H_2_ gas, MeOH, rt, 1 day, 66%; (**c**) neat, 3–6 h, reflux, 88%; (**d**) conc. HCl/conc. HNO_3_, 90 °C 1 h, rt 1 h, 30–47%; and (**e**) ethanol, reflux, 3–4 h, 24–77%.

Derivatives of the benzimidazole-4,7-dione (**10a**–**10b** and **11a**–**11i**) were synthesized following a previously reported procedure [17] shown in Scheme 1. From the starting compound, 1,4-dimethoxy-2,3-diaminobenzene (**7**)**,** cyclo-condensation reactions were performed using neat acetic acid and trifluoroacetic acid to produce 4,7-dimethoxy-2-methylbenzimidazole (**8a**) and 4,7-dimethoxy-2-trifluoromethylbenzimidazole (**8b**). Then, aqua regia (3:1 ratio of conc. HCl/conc. HNO_3_) was used for demethylation and chlorination to produce 5,6-dichloro-2-methylbenzimidazole-4,7-dione (**9a**) and 5,6-dichloro-2-trifluoromethylbenzimidazole-4,7-dione (**9b**). Various halide substituted aniline derivatives were reacted with **9a** and **9b** to afford the final compounds **10a**–**10b** and **11a**–**11k**.

### 2.3. Hit Optimization: Structure–Activity and Structure–Property Relationship (SAR and SPR) Studies

#### 2.3.1. The Importance of Halide Substitutions

The 3,4-difluoroaniline moiety of the hit compound **KCB-77033** was first modified to 4-fluoro (**10a**) and 4-chloro (**10b**) aniline groups, which showed approximately 4–5-fold decreased P2X3R antagonistic activities. The same profile of 2-trifluoromethyl derivatives was also observed from the comparison of compounds **11a**–**c**. In addition, 2-trifluoromehtyl derivative (**11a**) showed a much higher preference of metabolic stability profile compared with 2-methyl derivatives (**KCB-77033** and **10a**), which indicated that the 2-methyl group would be metabolically labile for phase 1 metabolism (Table 1). Therefore, various halide substitutions of the aniline group were further explored to increase P2X3R antagonistic activities while maintaining the 2-trifluoromethyl moiety for metabolic stability in the evaluation of the next series of compounds.

#### 2.3.2. Halide-Substituted Aniline Derivatives

Since trifluoromethyl substitution resulted in an almost 10-times increase in metabolic stability, further aniline derivatives were synthesized based on the fixed core structure of 2-trifluoromethylbenzimidazole-4,7-dione. In Table 2, various combinations of halide groups were introduced to develop aniline derivatives that were investigated using SAR studies as the P2X3R antagonistic activities seemed to vary according to the identity of the halide group used in substitution of the aniline moiety. As a result, the SAR of antagonistic activity profile was analyzed, and the following order determined: F^−^, Cl^−^ > F^−^, Br^−^ or I, Cl^−^ > di-Cl^−^ or mono-Cl^−^ or di-F^−^ > mono-F^−^. The 3-monochloro (**11b**), 3,4-difluoro (**11c**), and 3,4-dichloro (**11d**) substituted aniline derivatives showed slightly increased antagonistic activity compared with 3-monofluro analogue (**11a**). Next, fluoro- and chloro- disubstituted aniline derivatives, **11e**–**11h,** showed relatively higher antagonistic activities than variations based on other halides such as the bromine- or iodine-substituted aniline derivatives **11i**–**11k**. From the series of compounds in Table 1 and Table 2, **11g** and **11h** showed the greatest increase in antagonistic activity toward hP2X3R, with IC_50_ values of 526 and 541 nM, respectively. However, because the poor water solubility profile of the aniline derivatives (e.g., insoluble in 40% DMSO in water) would make further in vivo study difficult, it was decided that the incorporation of another heterocyclic moiety, namely, the piperidine group, would be attempted to address the solubility problem, rather than to continue further investigation of the aniline derivatives.

#### 2.3.3. Synthesis and Biological Evaluation of Piperidine Derivatives at hP2X3R

**Scheme 2:**
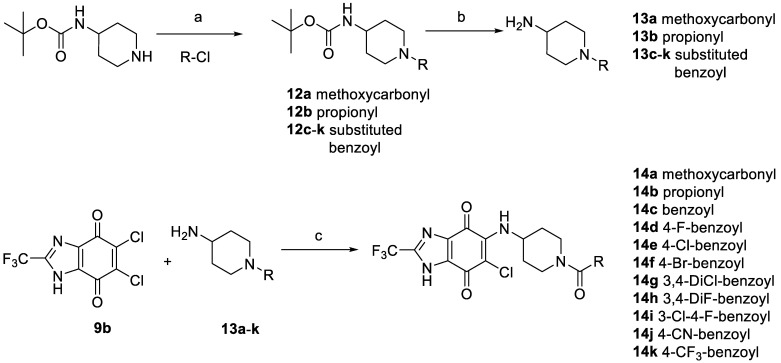
Scheme for synthesis of the benzimidazole-4,7-dione piperidine derivatives under the following conditions: (**a**) DCM, ice, 1 h, 95%; (**b**) TFA 30% in DCM or DCE, rt, 2 h, 99%; and (**c**) THF/DMF (10:1), rt, 1–2 h, 30–77%.

As shown in Scheme 2, various piperidine analogues were substituted at the core structure, 2-trifluoromethylbenzimidazole-4,7-dione. Starting from 4-(Boc-amino)piperidine, various methoxycarbonyl, propionyl and benzoyl groups were conjugated to yield **12a**–**k**, and subsequent deprotection reactions of their Boc groups was carried out using 30% TFA in DCM or DCE conditions to afford compounds **13a**–**k**. Finally, compounds **13a**–**k** were reacted with compound **9b** to yield compounds **14a**–**k**, which were evaluated using the same procedures of the P2X3R cell-based assay system.

**Figure 3:**
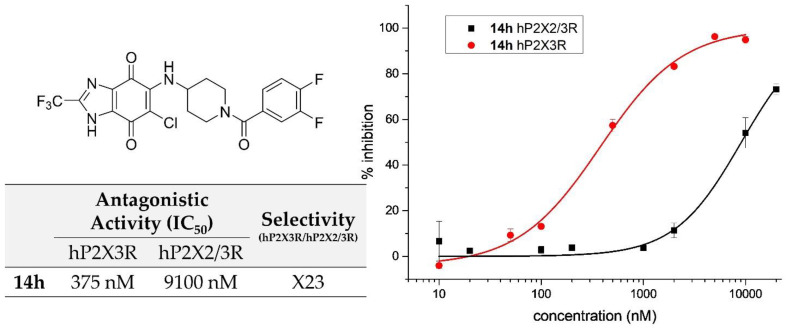
The in vitro antagonistic activity of compound **14h**.

In Table 3, comparing **14a**–**b** with **14c**, the compound with the benzoyl moiety (IC_50_ = 864 nM) had slightly higher potency compared to those with aliphatic acyl groups (IC_50_ = 979 and 921 nM) when considering the substituent at the piperidine terminal nitrogen. Thus, derivatives **14c**–**k** with various substituted benzoyl moieties were investigated to optimize P2X3R antagonistic activities. It seems that the size and electrostatic potential of substituents at the para-position of the benzoyl group affected the antagonistic activities with variations of different IC_50_ values. For example, relatively larger group containing analogues **14f** (-Br), **14j** (-CN), and **14k** (-CF_3_) showed decreased antagonistic activities compared to compounds with fluoro- or chloro- substitutions. Among the mono- and para-substituted analogues, the 4-fluoro derivative **14d** was the most active antagonist with an IC_50_ value of 450 nM. In the case of the compounds with dihalide substituents, **14g**–**i**, 3,4-difluoro substituted derivative **14h** showed further optimized antagonistic activity with an IC_50_ value of 375 nM. However, 3,4-dichloro (**14g**) or 3-chloro-4-fluoro (**14i**) substitutions did not contribute to increasing the antagonistic activities from the corresponding mono- and para-substituted compounds (**14e** and **14d**, respectively). The final optimized compound, **14h**, also displayed improved solubility (soluble in 30% DMSO in water) and a more than 23-fold selectivity versus P2X2/3R (IC_50_ = 9100 nM, Figure 3), and was selected for further investigation in pharmacokinetic (PK) and in vivo efficacy studies (Figure 3).

### 2.4. Pharmacokinetic and hERG Channel Studies of Compound ***14h***

The final optimized compound **14h** was first evaluated for its in vitro pharmacokinetic profiles, including determination of its metabolic stability in microsomal fractions and BBB-PAMPA permeability, resulting in very stable metabolic profiles being found for human, rat, and mouse species, albeit disappointingly low (<−6.0 logPe) as shown in Table 4. In the in vitro toxicity profiles, compound **14h** showed acceptable CYP450 inhibitory profiles at 10 μM with 6.78, 25.6, and 7.74% for 1A2, 2C19, and 2D6 CYP enzymes, respectively; however, over 50% inhibition was observed for the 2C9 and 3A4 CYP enzymes. For the evaluation of compound **14h** in the ligand-binding assay for hERG protein to predict cardiotoxicity, less than 1% inhibition for hERG channel binding was observed (Table 4).

**Figure 4:**
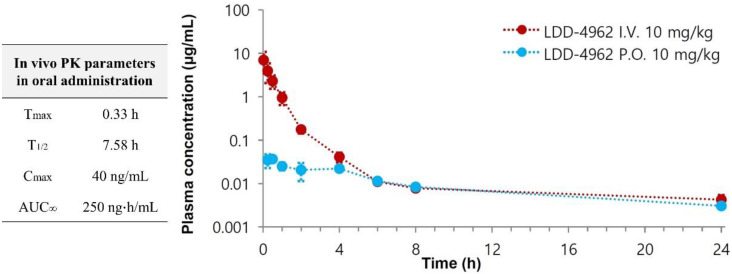
In vivo pharmacokinetic study of compound **14h**.

Next, compound **14h** was investigated for its in vivo pharmacokinetic study through both intravenous and oral administrations with 10 mpk doses. As shown in Figure 4, although the blood concentrations after intravenous administration were sufficient to allow systemic exposure for the compound, all the PK parameters from oral administration indicated poor profile, with short T_max_ (0.33 h) and very low blood concentrations in both C_max_ and AUC values (40 ng/mL and 250 ng·h/mL, respectively), concentrations that are not sufficient for evoking P2X3R antagonistic efficacy via oral administration. Therefore, it was decided that compound **14h** would be administered via intravenous injection for evaluation using in vivo pain models.

### 2.5. Antiallodynic Effect of Compound ***14h*** in Intravenous Administration of Animal Pain Models

**Figure 5:**
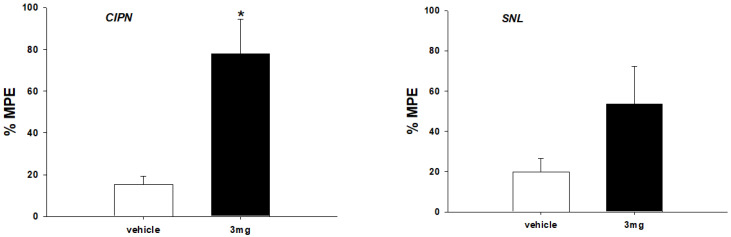
Antiallodynic effect of compound **14h** in animal pain models. Each bar represents the mean ± SEM of five rats. * *p* < 0.05, compared with vehicle.

The antiallodynic effect of compound **14h** was measured by counting the paw withdrawal responses to von Frey filaments in SNL and CIPN rats. Data are expressed as the maximal possible effect (% MPE). Each bar represents the mean ± SEM of five rats. * *p* < 0.05, compared with vehicle. Intravenous injection of compound **14h** (12 mg/kg) significantly attenuated mechanical allodynia in rats with the neuropathic pain and, hence, increased paw withdrawal threshold in CIPN rats but not SNL rats compared to the controls. The maximum possible effects of **14h** in CIPN and SNL rats were 78% and 54%, respectively (Figure 5).

## 3. Materials and Methods

### 3.1. Chemistry

All the chemicals and solvents were used from chemical suppliers without purification. Column chromatography was carried out for purification using precoated silica gel plates (MERCK silica gel 60; F254, 0.040–0.063 mm). NMR spectra were obtained using a JEOL ECS 400 NMR spectrometer (Tokyo, Japan) at a 1H frequency of 400 MHz and 13C frequency of 100 MHz. Proton and carbon chemical shifts were reported in parts per million (ppm) relative to an internal standard. Chemical shifts, multiplicities, and coupling constants (J) were reported and calculated using Delta 5.3.1 software provided by JEOL and ACD NMR processor academic edition software. Mass spectrometry of all synthesized compounds was carried out on a BEH C18 column (1.7 μm, 2.1 mm × 50 mm; Waters, Milford, MA, USA) maintained at 40 °C during separation under isocratic conditions (mobile phase A/mobile phase B = 20:80) using a Waters ACQUITY ultraperformance liquid chromatograph coupled to a triple quadrupole mass spectrometer (Micromass Quattro Micro, Waters). Mobile phase A consisted of water (LC–MS grade) with 0.1% formic acid (*v*/*v*) and mobile phase B consisted of CH3CN (LC–MS grade) with 0.1% formic acid (*v*/*v*); flow rate, 0.2 mL/min.

### 3.2. General Procedure

To derive compounds **10a**–**10b** and **11a**–**11k**, the starting materials **9a** and **9b** (1.0 equiv.) were stirred with the appropriate anilines (1.2–1.3 equiv.) in TEA (1.5 equiv.) and EtOH solvent for reflux. The reaction times ranged from 6 h to overnight in TLC monitoring. To quench reactions, the reaction mixture was poured into ammonium chloride solution and extracted using ethyl acetate. The organic layer was washed successively with water, dried over sodium sulfate anhydrous filter, and evaporated under reduced pressure to afford the product. The reaction mixtures were purified by column chromatography in hexane–ethyl acetate, chloroform–methanol, or ammonia-saturated chloroform–methanol systems.

To derive compounds **14a**–**14k**, **9b** (1.0 equiv.) was mixed with various moieties of **13a**–**13k** (1.2 equiv.) in DCM:THF = 10:1 solvent and stirred for 1–2 h. The reaction mixture was evaporated under reduced pressure and high vacuum and directly added into the column chromatography for purification in chloroform–methanol system. All analytical data are reported in the Supplementary Materials.

### 3.3. Synthesis of Compounds

*6-Chloro-5-((4-fluorophenyl)amino)-2-methyl-1H-benzo[d]imidazole-4,7-dione* (**10a**) [18]. Following the general procedure for the synthesis of **10a**–**b**, the substitution reaction of **9a** with 4-fluoroaniline afforded **10a**. Red-purple powder. Yield 67%; ^1^H NMR (400 MHz, DMSO-*d*_6_) δ ppm 2.32 (s, 4 H) 7.03–7.16 (m, 4 H); LC/MS (ESI, *m*/*z*) 303.7 [M − H]^−^ 305.8 [M + H]^+^.

*6-Chloro-5-((4-chlorophenyl)amino)-2-methyl-1H-benzo[d]imidazole-4,7-dione* (**10b**) [18]. Following the general procedure for the synthesis of **10a**–**b**, the substitution re-action of **9a** with 4-chloroaniline afforded **10b**. Red-purple powder. Yield 71%; ^1^H NMR (400 MHz, DMSO-*d*_6_) δ ppm 13.6 (s, 1H, NH), 9.0 (s, 1H, NH), 7.0–7.3 (dd, *J* = 2.4, 8.4 Hz, 4H, Ph-H), 2.2 (s, 3H, CH3); LC/MS (ESI, *m*/*z*) 319.8 [M − H]^−^ 321.8 [M + H]^+^.

*6-Chloro-5-((4-fluorophenyl)amino)-2-(trifluoromethyl)-1H-benzo[d]imidazole-4,7-dione* (**11a**) [17]. Following the general procedure for the synthesis of **11a**–**k**, the substitution reaction of **9b** with 4-fluoroaniline afforded **11a**. Purple powder. Yield 41%; ^1^H NMR (400 MHz, METHANOL-*d*_4_) δ ppm 6.98–7.13 (m, 4 H); LC/MS (ESI, *m*/*z*) 358.3 [M − H]^−^ 360.0 [M + H]^+^.

*6-Chloro-5-((4-chlorophenyl)amino)-2-(trifluoromethyl)-1H-benzo[d]imidazole-4,7-dione* (**11b**) [17]. Following the general procedure for the synthesis of **11a**–**k**, the substitution reaction of **9b** with 4-chloroaniline afforded **11b**. Purple powder. Yield 56%; ^1^H NMR (400 MHz, METHANOL-*d*_4_) δ ppm 7.01–7.10 (m, 2 H) 7.23–7.31 (m, 2 H); LC/MS (ESI, *m*/*z*) 374.3 [M − H]^−^ 375.8 [M + H]^+^.

*6-Chloro-5-((3,4-difluorophenyl)amino)-2-(trifluoromethyl)-1H-benzo[d]imidazole-4,7-dione* (**11c**). Following the general procedure for the synthesis of **11a**–**k**, the substitution reaction of **9b** with 3,4-difluoroaniline afforded **11c**. Dark red powder. Yield 31%; ^1^H NMR (400 MHz, ACETONE-*d*_6_) δ ppm 7.00–7.07 (m, 1 H) 7.20 (ddd, *J* = 11.91, 7.33, 2.75 Hz, 1 H) 7.24–7.34 (m, 1 H) 8.51 (s, 1 H); ^13^C NMR (100 MHz, ACETONE-*d*_6_) δ 174.18, 173.99, 142.43, 141.55, 137.99, 135.97, 135.86, 121.28, 120.73, 118.60, 116.57, 116.39, 113.53, 113.33, 111.59; LC/MS (ESI, *m*/*z*) 376.3 [M − H]^−^ 377.1 [M + H]^+^.

*6-Chloro-5-((3,4-dichlorophenyl)amino)-2-(trifluoromethyl)-1H-benzo[d]imidazole-4,7-dione* (**11d**). Following the general procedure for the synthesis of **11a**–**k**, the substitution reaction of **9b** with 3,4-dichloroaniline afforded **11d**. Red-purple powder. Yield 38%; ^1^H NMR (400 MHz, METHANOL-*d*_4_) δ ppm 7.01 (dd, *J* = 8.47, 2.52 Hz, 1 H) 7.24 (d, *J* = 2.29 Hz, 1 H) 7.43 (d, *J* = 8.70 Hz, 1 H); ^13^C NMR (100 MHz, METHANOL-*d*_4_) δ 174.16, 173.42, 142.22, 139.94, 139.24, 137.54, 131.91, 129.96, 127.88, 125.59, 123.75, 120.98, 113.37, 2 carbon peaks in 125.59 ppm overlapped; LC/MS (ESI, *m*/*z*) 408.0 [M − H]^−^ 410.2 [M + H]^+^.

*6-Chloro-5-((2-chloro-4-fluorophenyl)amino)-2-(trifluoromethyl)-1H-benzo[d]imidazole-4,7-dione* (**11e**). Following the general procedure for the synthesis of **11a**–**k**, the substitution reaction of **9b** with 2-chloro-4-fluoroaniline afforded **11e**. Red-purple powder. Yield 41%; ^1^H NMR (400 MHz, METHANOL-*d*_4_) δ ppm 7.09 (td, *J* = 8.47, 2.75 Hz, 1 H) 7.24–7.35 (m, 2 H); ^13^C NMR (100 MHz, METHANOL-*d*_4_) δ 173.34, 173.26, 142.69, 140.09, 136.91, 132.87, 131.87, 131.76, 129.34, 120.64, 117.95, 116.14, 115.89, 113.73, 113.50, 109.94; LC/MS (ESI, *m*/*z*) 392.2 [M − H]^−^ 394.1 [M + H]^+^.

*6-Chloro-5-((3-chloro-4-fluorophenyl)amino)-2-(trifluoromethyl)-1H-benzo[d]imidazole-4,7-dione* (**11f**). Following the general procedure for the synthesis of **11a**–**k**, the substitution reaction of **9b** with 3-chloro-4-fluoroaniline afforded **11f**. Red-purple powder. Yield 46%; ^1^H NMR (400 MHz, METHANOL-*d*_4_) δ ppm 7.05 (ddd, *J* = 8.93, 4.12, 2.52 Hz, 1 H) 7.13–7.24 (m, 2 H); ^13^C NMR (100 MHz, METHANOL-*d*_4_) δ 178.81, 142.11, 140.72, 137.51, 135.81, 126.00, 124.28, 124.21, 119.86, 119.67, 115.65, 115.42, 111.30, 2 carbon peaks in 178.81 ppm overlapped; LC/MS (ESI, *m*/*z*) 392.2 [M − H]^−^ 393.7 [M + H]^+^.

*6-Chloro-5-((4-chloro-2-fluorophenyl)amino)-2-(trifluoromethyl)-1H-benzo[d]imidazole-4,7-dione* (**11g**). Following the general procedure for the synthesis of **11a**–**k**, the substitution reaction of **9b** with 4-chloro-2-fluoroaniline afforded **11g**. Red-purple powder. Yield 52%; ^1^H NMR (400 MHz, DMSO-*d*_6_) δ ppm 7.20–7.34 (m, 2 H) 7.44 (dd, *J* = 10.30, 2.06 Hz, 1 H); ^13^C NMR (100 MHz, DMSO-*d*_6_) δ 174.06, 173.44, 142.84, 142.73, 138.43, 130.20, 130.09, 128.65, 127.65, 127.53, 124.67, 116.48, 116.25, 111.93; LC/MS (ESI, *m*/*z*) 392.2 [M − H]^−^ 394.1 [M + H]^+^.

*6-Chloro-5-((4-chloro-3-fluorophenyl)amino)-2-(trifluoromethyl)-1H-benzo[d]imidazole-4,7-dione* (**11h**). Following the general procedure for the synthesis of **11a**–**k**, the substitution reaction of **9b** with 4-chlro-3-fluoroaniline afforded **11h**. Red-purple powder. Yield 39%; ^1^H NMR (400 MHz, METHANOL-*d*_4_) δ ppm 6.86 (dd, *J* = 8.70, 0.92 Hz, 1 H) 6.89–6.98 (m, 1 H) 7.36 (t, *J* = 8.47 Hz, 1 H); ^13^C NMR (100 MHz, METHANOL-*d*_4_) δ 175.09, 174.29, 143.04, 141.47, 139.63, 139.05, 129.36, 119.91, 119.87, 115.26, 115.07, 113.25, 111.45, 111.21; LC/MS (ESI, *m*/*z*) 392.2 [M − H]^−^ 394.1 [M + H]^+^.

*5-((3-Bromo-4-fluorophenyl)amino)-6-chloro-2-(trifluoromethyl)-1H-benzo[d]imidazole-4,7-dione* (**11i**). Following the general procedure for the synthesis of **11a**–**k**, the substitution reaction of **9b** with 3-bromo-4-fluoroaniline afforded **11i**. Purple powder. Yield 63%; ^1^H NMR (400 MHz, METHANOL-*d*_4_) δ ppm 7.06–7.11 (m, 1 H) 7.13–7.18 (m, 1 H) 7.34 (dd, *J* = 5.95, 2.29 Hz, 1 H); ^13^C NMR (100 MHz, METHANOL-*d*_4_) δ 174.43, 173.89, 142.12, 141.68, 137.99, 136.05, 128.83, 124.98, 124.90, 115.41, 115.17, 111.07, 107.54, 107.32; LC/MS (ESI, *m*/*z*) 436.1 [M − H]^−^ 437.8 [M + H]^+^.

*5-((4-Bromo-3-fluorophenyl)amino)-6-chloro-2-(trifluoromethyl)-1H-benzo[d]imidazole-4,7-dione* (**11j**). Following the general procedure for the synthesis of **11a**–**k**, the substitution reaction of **9b** with 4-bromo-3-fluoroaniline afforded **11j**. Red-purple powder. Yield 51%; ^1^H NMR (400 MHz, METHANOL-*d*_4_) δ ppm 6.77–6.88 (m, 1 H) 6.94 (dd, *J* = 10.08, 2.29 Hz, 1 H) 7.41–7.60 (m, 1 H); ^13^C NMR (100 MHz, METHANOL-*d*_4_) δ 174.81, 174.27, 148.44, 148.06, 142.67, 141.38, 140.45, 140.35, 138.87, 132.27, 120.21, 113.56, 111.23, 110.98; LC/MS (ESI, *m*/*z*) 436.1 [M − H]^−^ 438.0 [M + H]^+^.

*6-Chloro-5-((4-chloro-2-iodophenyl)amino)-2-(trifluoromethyl)-1H-benzo[d]imidazole-4,7-dione* (**11k**). Following the general procedure for the synthesis of **11a**–**k**, the substitution reaction of **9b** with 4-chloro-2-iodoaniline afforded **11k**. Purple powder. Yield 24%; ^1^H NMR (400 MHz, METHANOL-*d*_4_) δ ppm 7.14 (d, *J* = 8.24 Hz, 1 H) 7.38 (d, *J* = 10.99 Hz, 1 H) 7.88 (d, *J* = 2.29 Hz, 1 H); ^13^C NMR (100 MHz, METHANOL-*d*_4_) δ 175.75, 174.94, 141.80, 140.10, 138.43, 137.95, 131.35, 127.92, 127.78, 127.54, 125.09, 117.77, 110.53, 96.60; LC/MS (ESI, *m*/*z*) 499.8 [M − H]^−^ 501.9 [M + H]^+^.

*Methyl-4-((6-chloro-4,7-dioxo-2-(trifluoromethyl)-4,7-dihydro-1H-benzo[d]imidazol-5-yl)amino)piperidine-1-carboxylate* (**14a**). Following the general procedure for the synthesis of **14a**–**k**, the substitution reaction of **9b** with **13a** afforded **14a**. Purple powder. Yield 37%; ^1^H NMR (400 MHz, METHANOL-*d*_4_) δ ppm 1.52 (d, *J* = 15.11 Hz, 2 H) 2.04 (br. s., 2 H) 3.00 (br. s., 2 H) 3.69 (s, 3 H) 4.09 (br. s., 2 H) 4.61 (s, 1 H); ^13^C NMR (100 MHz, METHANOL-*d*_4_) δ 175.76, 173.83, 169.11, 156.24, 144.98, 143.53, 138.29, 122.12, 119.44, 51.97, 50.52, 42.29, 32.84; LC/MS (ESI, *m*/*z*) 404.9 [M − H]^−^ 406.8 [M + H]^+^.

*6-Chloro-5-((1-propionylpiperidin-4-yl)amino)-2-(trifluoromethyl)-1H-benzo[d]imidazole-4,7-dione* (**14b**). Following the general procedure for the synthesis of **14a**–**k**, the substitution reaction of **9b** with **13b** afforded **14b**. Red-purple powder. Yield 30%; ^1^H NMR (400 MHz, METHANOL-*d*_4_) δ ppm 1.06–1.16 (m, 3 H) 1.42–1.56 (m, 1 H) 1.56–1.64 (m, 1 H) 2.00–2.15 (m, 2 H) 2.44 (q, *J* = 7.48 Hz, 2 H) 2.82 (t, *J* = 14.20 Hz, 1 H) 3.22 (t, *J* = 11.68 Hz, 1 H) 3.98 (d, *J* = 15.11 Hz, 1 H) 4.51 (d, *J* = 14.66 Hz, 1 H) 4.67 (s, 1 H); ^13^C NMR (100 MHz, METHANOL-*d*_4_) δ 175.44, 173.79, 173.41, 149.41, 144.47, 143.58, 138.12, 121.97, 119.29, 50.62, 43.92, 40.20, 25.95, 8.64; LC/MS (ESI, *m*/*z*) 403.3 [M − H]^−^ 405.4 [M + H]^+^.

*5-((1-Benzoylpiperidin-4-yl)amino)-6-chloro-2-(trifluoromethyl)-1H-benzo[d]imidazole-4,7-dione* (**14c**). Following the general procedure for the synthesis of **14a**–**k**, the substitution reaction of **9b** with **13c** afforded **14c**. Purple powder. Yield 43%; ^1^H NMR (400 MHz, METHANOL-*d*_4_) δ ppm 1.66 (d, *J* = 13.74 Hz, 2 H) 2.02 (br. s., 1 H) 2.20 (br. s., 1 H) 3.05 (br. s., 1 H) 3.26 (br. s., 1 H) 3.76 (br. s., 1 H) 4.63 (br. s., 1 H) 4.75 (s, 1 H) 7.42–7.50 (m, 5 H); ^13^C NMR (100 MHz, METHANOL-*d*_4_) δ 173.56, 171.21, 146.20, 143.85, 141.35, 136.89, 135.64, 129.76, 129.37, 128.41, 126.51, 50.88, 46.23, 40.68, 2 carbon peaks in 173.56 ppm overlapped; LC/MS (ESI, *m*/*z*) 451.5 [M − H]^−^ 453.1 [M + H]^+^.

*6-Chloro-5-((1-(4-fluorobenzoyl)piperidin-4-yl)amino)-2-(trifluoromethyl)-1H-benzo[d]imidazole-4,7-dione* (**14d**). Following the general procedure for the synthesis of **14a**–**k**, the substitution reaction of **9b** with **13d** afforded **14d**. Red-purple powder. Yield 67%; ^1^H NMR (400 MHz, ACETONE-*d*_6_) δ ppm 1.65–1.85 (m, 2 H) 2.11 (br. s., 2 H) 2.98–3.28 (m, 2 H) 3.80 (br. s., 1 H) 4.56 (br. s., 1 H) 4.76 (br. s., 1 H) 6.47 (br. s., 1 H) 7.14–7.24 (m, 2 H) 7.45–7.54 (m, 2 H); ^13^C NMR (100 MHz, DMSO-*d*_6_) δ 174.06, 173.14, 168.60, 144.11, 137.82, 137.76, 133.13, 133.10, 129.79, 129.70, 116.05, 115.83, 105.68, 51.15, 49.12, 46.49 LC/MS (ESI, *m*/*z*) 469.4 [M − H]^−^ 471.1 [M + H]^+^.

*6-Chloro-5-((1-(4-chlorobenzoyl)piperidin-4-yl)amino)-2-(trifluoromethyl)-1H-benzo[d]imidazole-4,7-dione* (**14e**). Following the general procedure for the synthesis of **14a**–**k**, the substitution reaction of **9b** with **13e** afforded **14e**. Purple powder. Yield 77%; ^1^H NMR (400 MHz, METHANOL-*d*_4_) δ ppm 1.65 (br. s., 2 H) 2.03 (br. s., 1 H) 2.16 (br. s., 1 H) 3.04 (br. s., 1 H) 3.27 (br. s., 1 H) 3.74 (br. s., 1 H) 4.61 (br. s., 1 H) 4.73 (s, 1 H) 7.42–7.45 (m, 2 H) 7.46–7.50 (m, 2 H); ^13^C NMR (100 MHz, METHANOL-*d*_4_) δ 173.72, 173.46, 170.02, 143.95, 141.67, 136.97, 135.70, 134.19, 128.61, 128.37, 50.84, 46.27, 40.77, 2 carbon peaks in 141.67 ppm are overlapped; LC/MS (ESI, *m*/*z*) 485.3 [M − H]^−^ 487.1 [M + H]^+^.

*5-((1-(4-Bromobenzoyl)piperidin-4-yl)amino)-6-chloro-2-(trifluoromethyl)-1H-benzo[d]imidazole-4,7-dione* (**14f**). Following the general procedure for the synthesis of **14a**–**k**, the substitution reaction of **9b** with **13f** afforded **14f**. Purple powder. Yield 68%; ^1^H NMR (400 MHz, METHANOL-*d*_4_) δ ppm 1.65 (br. s., 2 H) 2.04 (br. s., 1 H) 2.20 (br. s., 1 H) 3.06 (br. s., 1 H) 3.27 (br. s., 1 H) 3.73 (br. s., 1 H) 4.61 (br. s., 1 H) 4.76 (s, 1 H) 7.38 (d, *J* = 8.70 Hz, 2 H) 7.65 (d, *J* = 8.24 Hz, 2 H); ^13^C NMR (100 MHz, METHANOL-*d*_4_) δ 173.46, 173.36, 170.07, 143.99, 141.08, 138.59, 136.80, 134.63, 131.63, 128.51, 123.81, 120.86, 118.17, 115.484, 50.88, 46.25, 40.72; LC/MS (ESI, *m*/*z*) 529.2 [M − H]^−^ 532.9 [M + H]^+^.

*6-Chloro-5-((1-(3,4-dichlorobenzoyl)piperidin-4-yl)amino)-2-(trifluoromethyl)-1H-benzo[d]imidazole-4,7-dione* (**14g**). Following the general procedure for the synthesis of **14a**–**k**, the substitution reaction of **9b** with **13g** afforded **14g**. Purple powder. Yield 66%; ^1^H NMR (400 MHz, METHANOL-*d*_4_) δ ppm 1.68 (br. s., 2 H) 2.03 (br. s., 1 H) 2.18 (br. s., 1 H) 3.05 (br. s., 1 H) 3.28 (br. s., 1 H) 3.72 (br. s., 1 H) 4.60 (br. s., 1 H) 4.76 (s, 1 H) 7.38 (dd, *J* = 8.24, 1.83 Hz, 1 H) 7.58–7.68 (m, 2 H); ^13^C NMR (100 MHz, METHANOL-*d*_4_) δ 174.24, 173.44, 168.49, 144.06, 142.34, 137.26, 135.90, 133.71, 132.55, 130.69, 128.84, 126.41, 123.50, 122.12, 118.55, 50.77, 46.21, 40.87, 40.72; LC/MS (ESI, *m*/*z*) 519.2 [M − H]^−^ 521.0 [M + H]^+^.

*6-Chloro-5-((1-(3,4-difluorobenzoyl)piperidin-4-yl)amino)-2-(trifluoromethyl)-1H-benzo[d]imidazole-4,7-dione* (**14h**). Following the general procedure for the synthesis of **14a**–**k**, the substitution reaction of **9b** with **13h** afforded **14h**. Red-purple powder. Yield 67%; ^1^H NMR (400 MHz, METHANOL-*d*_4_) δ ppm 1.57 (br. s., 2 H) 1.99 (br. s., 1 H) 2.11 (br. s., 1 H) 3.00 (br. s., 1 H) 3.20 (br. s., 1 H) 3.69 (br. s., 1 H) 4.50 (br. s., 1 H) 4.61 (s, 1 H) 7.18–7.24 (m, 1 H) 7.26–7.41 (m, 2 H); ^13^C NMR (100 MHz, METHANOL-*d*_4_) δ 173.33, 173.02, 168.73, 143.97, 140.64, 136.48, 132.83, 132.78, 123.74, 123.70, 120.67, 117.99, 117.63, 117.46, 116.47, 116.29, 50.91, 46.24, 40.87; LC/MS (ESI, *m*/*z*) 487.4 [M − H]^−^ 489.1 [M + H]^+^.

*6-Chloro-5-((1-(3-chloro-4-fluorobenzoyl)piperidin-4-yl)amino)-2-(trifluoromethyl)-1H-benzo[d]imidazole-4,7-dione* (**14i**). Following the general procedure for the synthesis of **14a**–**k**, the substitution reaction of **9b** with **13i** afforded **14i**. Red-purple powder. Yield 75%; ^1^H NMR (400 MHz, ACETONE-*d*_6_) δ ppm 1.68–1.84 (m, 2 H) 2.13 (br. s., 1 H) 3.04 (br. s., 2 H) 3.82 (br. s., 1 H) 4.51 (br. s., 1 H) 4.76 (br. s., 1 H) 7.35–7.41 (m, 1 H) 7.47 (ddd, *J* = 8.47, 4.58, 2.06 Hz, 1 H) 7.58–7.62 (m, 1 H); ^13^C NMR (100 MHz, METHANOL-*d*_4_) δ 173.49, 168.67, 136.98, 133.14, 129.42, 127.38, 127.31, 127.10, 121.10, 121.01, 120.92, 116.84, 116.73, 116.62, 50.79, 46.33, 41.43, 2 carbon peaks in 173.49 ppm overlapped; LC/MS (ESI, *m*/*z*) 505.1 [M − H]^−^ 503.3 [M + H]^+^.

*4-(4-((6-Chloro-4,7-dioxo-2-(trifluoromethyl)-4,7-dihydro-1H-benzo[d]imidazol-5-yl)amino)piperidine-1-carbonyl)benzonitrile* (**14j**). Following the general procedure for the synthesis of **14a**–**k**, the substitution reaction of **9b** with **13j** afforded **14j**. Purple powder. Yield 70%; ^1^H NMR (400 MHz, METHANOL-*d*_4_) δ ppm 1.62–1.76 (m, 2 H) 2.00–2.07 (m, 1 H) 2.20 (d, *J* = 10.99 Hz, 1 H) 3.06 (br. s., 1 H) 3.22–3.30 (m, 1 H) 3.66 (d, *J* = 11.45 Hz, 1 H) 4.65 (d, *J* = 12.82 Hz, 1 H) 4.78 (s, 1 H) 7.62 (d, *J* = 8.70 Hz, 2 H) 7.86 (d, *J* = 8.24 Hz, 2 H); ^13^C NMR (100 MHz, METHANOL-*d*_4_) δ 173.55, 169.09, 143.89, 143.00, 142.10, 140.21, 137.57, 132.43, 129.39, 127.46, 117.72, 113.36, 50.66, 46.07, 40.65, 2 carbon peaks in 173.55 ppm overlapped; LC/MS (ESI, *m*/*z*) 476.6 [M − H]^−^ 478.3 [M + H]^+^.

*6-Chloro-2-(trifluoromethyl)-5-((1-(4-(trifluoromethyl)benzoyl)piperidin-4-yl)amino)-1H-benzo[d]imidazole-4,7-dione* (**14k**). Following the general procedure for the synthesis of **14a**–**k**, the substitution reaction of **9b** with **13k** afforded **14k**. Purple powder. Yield 72%; ^1^H NMR (400 MHz, METHANOL-*d*_4_) δ ppm 1.64 (d, *J* = 6.87 Hz, 1 H) 1.72 (d, *J* = 11.45 Hz, 1 H) 2.04 (br. s., 2 H) 2.21 (d, *J* = 6.87 Hz, 1 H) 2.99–3.15 (m, 1 H) 3.15–3.29 (m, 1 H) 3.67 (d, *J* = 12.37 Hz, 1 H) 4.65 (d, *J* = 14.20 Hz, 1 H) 4.76 (s, 1 H) 7.64 (m, *J* = 7.79 Hz, 2 H) 7.79 (m, *J* = 8.24 Hz, 2 H); ^13^C NMR (100 MHz, METHANOL-*d*_4_) δ 173.51, 169.56, 143.96, 139.61, 136.98, 131.58, 131.25, 127.26, 125.48, 125.44, 50.81, 46.14, 40.66, 2 carbon peaks in 173.51 ppm overlapped; LC/MS (ESI, *m*/*z*) 519.3 [M − H]^−^ 521.4 [M + H]^+^.

### 3.4. Cell-Based Assay: Measurement of Calcium Influx by Fluorescence-Based Fluo-4 Dye Uptake in HEK293 Cells Expressing hP2X2/3 and hP2X3 Receptors

Human P2X3-expressing HEK293 cells were grown in DMEM supplemented with 10% fetal bovine serum and 1% antibiotic–antimycotic as a monolayer culture at 37 °C in a humidified atmosphere of 5% CO_2_. The 96-well black plates used in the experiments were prepared by coating them with 1× poly-D-lysine (A3890401, Thermo-Fisher Scientific Korea, Seoul, South Korea). An aliquot of 40 μL 1× poly-D-lysine was used to treat each of the wells in a flat orientation. After leaving the plates for at least 4 h to absorb the poly-D-lysine, the plates were allowed to dry for at least 6 h. The DMEM used in the culture of HEK293 cells was removed from the dish, followed by application of 3 mL 1× PBS for washing. Removal of 1× PBS was followed by application of 1 mL trypsin/EDTA, and the cells were collected by centrifugation at 1200 rpm for 2 min. The supernatant was removed, and 1 mL fresh DMEM added. The cells were resuspended in media and plates prepared with 4.8 × 10^4^ cells/well in 56 μL/well. After 18 h of cell culture in the 96-well black plate, 56 μL of Fluo-4 dye (Fluo-4 Direct^TM^ Calcium Assay Kit, F10471, Invitrogen, Thermo-Fisher Scientific Korea, Seoul, South Korea) was added to each well, prepared according to the manufacturer’s protocol, and incubated for 50 min~1 h under culturing conditions. Then, 12.4 μL of the appropriate compound solution was added to each well, and the positive control was AF353 (A1612, Sigma Aldrich Korea, Seoul, South Korea) and the negative control was 5% DMSO. Plates were incubated at room temperature and sealed to avoid interference from light for 50 min~1 h. Flexstation 3 and its software provided by Molecular Devices was used for the measurement of fluorescence in real time of treatments using prepared α,β-meATP (M6517, Sigma Aldrich) solution to evoke calcium influx and increased cell membrane potential.

### 3.5. Pharmacokinetic Studies

Experiments were used to determine any changes during in vivo pharmacokinetics, such as absorption, distribution, metabolism, and excretion of compounds, which are correlated with drug efficacy and toxicity. Pharmacokinetic experiments using rats were mainly conducted using 8-week-old SD male rats (Osan, Kyungki-Do, Korea). A tube was inserted into the femoral vein, the intravenous administration group was administered through the tube, and the oral administration group was administered the drug orally using oral gavage (1–2 mL/kg). Blood collection for the drug was performed at fixed times (0.33, 0.25, 0.5, 1, 2, 4, 6, 8, and 24 h) from the time when the tube was inserted into the femoral vein. Blood was centrifuged to separate plasma, and plasma and urine samples were pretreated (minimum 0.5 ng/mL and maximum 8000 ng/mL) using an appropriate organic solvent. For sample preparation, the working solution of rat blank plasma was spiked, followed by preparation of plasma samples with solid phase extraction. Next, the concentration of 100 μL eluent was analyzed by LC–MS/MS (Mass spectrometry 4000 Qtrap with HPLC, Agilent 1200, Santa Clara, CA, USA). The noncompartmental pharmacokinetic parameters were calculated from the blood concentration–time data of the drug analyzed using the noncompartmental analysis model of WinNonlin (Pharsight ver 6.4, Princeton, NJ, USA).

### 3.6. Neuropathic Pain Model and Behavioral Assessment

The experimental protocol (CNUHIACUC-20039) was approved by the Institutional Animal Care and Use Committee of Chonnam National University Hospital. Animal models of neuropathic pain (NP) were constructed by spinal nerve ligation (SNL) or chemotherapeutics injection (chemotherapy-induced peripheral neuropathy, CIPN) in male Sprague-Dawley rats (weight, 150–180 g). Briefly, under sevoflurane anesthesia, the left L5 and L6 spinal nerves near the vertebral column were separated and tightly ligated using 6-0 silk. Cisplatin was administered intraperitoneally at 2 mg/kg once daily for 4 days. The paw withdrawal threshold (PWT) to mechanical stimuli was measured using calibrated 0.41–15.2 g von Frey filaments (Stoelting, Wood Dale, IL, USA), as described previously [19]. Mechanical allodynia was defined as a PWT < 4 or 5 g in SNL and CIPN models, respectively. The antiallodynic effects of the intravenous vehicle and LDD4692 (3mg/rat) were evaluated and expressed as the percentage of the maximal possible effect (% MPE). % MPE = [(post drug PWT − post injured baseline PWT)/(cutoff PWT − post injured baseline PWT)] × 100

### 3.7. Statistical Analysis of Antagonistic Activities and Behavioral Assessments of Pain Models

For the analysis of antagonistic activities, all data were calculated by Origin Pro 9.1 software provided by Originlab and the fitting of graphs was proceeded by the growth/sigmoidal and hill1 process within no weights for data selections. For the analysis of the behavioral assessments of pain models, all data are expressed as mean ± SEM. The dose–response data are presented as percentages of the maximum possible effect. Statistical significance was analyzed by independent *t*-test. The criterion for statistical significance was *p* < 0.05. The statistical analysis was performed using SPSS 25.0 software (IBM Corp., Armonk, NY, USA).

## 4. Conclusions

In this study, a benzimidazole-4,7-dione hit compound, KCB-77033, as a P2X3R antagonist with an IC_50_ value of 1030 nM, was identified from the screening of 8364 random compounds in the Korea Chemical Bank and further optimized for the improvement of antagonistic activities as well as drug physicochemical properties, including metabolic stability. As a result, compound 14h was identified through final optimizations with 2-trifluoromethyl and dihalide-substituted benzoyl piperidine groups at the 2 and 5 positions of the core skeleton based on its metabolic stability (>92% remaining after 30 min incubation) and biological activities (P2X3R IC_50_ = 375 nM) with improved solubility (soluble in 30% DMSO in water). Furthermore, compound 14h showed an in vivo pain relief effect, with a statistically meaningful % MPE value of 78% in the CIPN model, which is for the neuropathic pain induced by cisplatin. This study presents a new chemical entity in the field of new drug discovery programs for P2X3R antagonists, which have been pursued for the treatment of neurological disorders, including neuropathic pains and chronic cough, among others.

## Figures and Tables

**Table 1:**
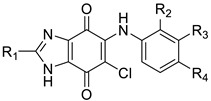
The antagonistic activity and metabolic stability profile of the first set of derivatives.

No.	R1	R2	R3	R4	hP2X3R	Metabolic Stability		
					IC_50_ (nM)	Mouse (%)	Rat (%)	Human (%)
**KCB-77033**	CH_3_	H	F	F	1030 ± 73	5.2 ± 1.6	3.4 ± 0.1	9.0 ± 1.5
**10a**	CH_3_	H	H	F	4910 ± 821	18.4 ± 2.7	26.2 ± 3.4	43.1 ± 4.7
**10b**	CH_3_	H	H	Cl	3720 ± 731	-	-	-
**11a**	CF_3_	H	H	F	1020 ± 111	95.7 ± 17.2	>99	81.4 ± 1.9
**11b**	CF_3_	H	H	Cl	825 ± 70	-	-	-
**11c**	CF_3_	H	F	F	834 ± 117	-	-	-

(-: not determined).

**Table 2:**
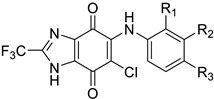
The antagonistic activity profile of halide-substituted aniline derivatives.

No.	R1	R2	R3	hP2X3R
				IC_50_ (nM)
**11a**	H	H	F	1020 ± 111
**11b**	H	H	Cl	825 ± 70
**11c**	H	F	F	834 ± 117
**11d**	H	Cl	Cl	808 ± 75
**11e**	Cl	H	F	660 ± 113
**11f**	H	Cl	F	703 ± 96
**11g**	F	H	Cl	526 ± 141
**11h**	H	F	Cl	541 ± 103
**11i**	H	Br	F	1040 ± 221
**11j**	H	F	Br	782 ± 139
**11k**	I	H	Cl	774 ± 166

**Table 3:**
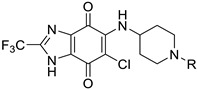
The antagonistic activity profile of various piperidine derivatives.

No.	R	hP2X3R
		IC_50_ (nM)
**14a**	methoxycarbonyl	979 ± 104
**14b**	propionyl	921 ± 117
**14c**	benzoyl	864 ± 112
**14d**	4-fluorobenzoyl	450 ± 59
**14e**	4-chlorobenzoyl	481 ± 20
**14f**	4-bromobenzoyl	804 ± 91
**14g**	3,4-dichlorobenzoyl	577 ± 66
**14h**	3,4-difluorobenzoyl	375 ± 33
**14i**	3-chloro-4-fluorobenzoyl	505 ± 51
**14j**	4-cyanobenzoyl	4440 ± 480
**14k**	4-trifluoromethylbenzoyl	>10,000

**Table 4:** In vitro pharmacokinetic and hERG channel studies of compound **14h**.

Property	Condition	Parameter	14h
**Metabolic Stability** **(Microsomal Fr.)**	Human	% remaining after 30 min	>99%
	Rat		>99%
	Mouse		92.8%
**CYP450 inhibition**	1A2	Compound 10 μM	6.78%
	2C9		79.8%
	2C19		25.6%
	2D6		7.74%
	3A4		55.4%
**BBB-PAMPA**	UV/vis	logPe	<−6.000
	spectrophotometry		
**hERG Channel**	ligand-binding assay	Compound 10 μM	<1%

## Data Availability

The presented data are available in this article and Supplementary Materials.

## Supplementary Materials

The following are available online. General procedures and copies of ^1^H, ^13^C NMR spectra, and LCMS data for all new compounds.

## Sample Availability

Samples of all compounds are available from the authors.
