# Supplementary material for: Synthesis and Structure–Activity Relationship Studies of Benzimidazole-4,7-dione-Based P2X3 Receptor Antagonists as Novel Anti-Nociceptive Agents
Source: Molecules. 2022 Feb 16;27(4):1337. doi: 10.3390/molecules27041337 (PMC8877008; doi:10.3390/molecules27041337)
Supplement: Supplementary file 1 [file molecules-27-01337-s001.zip › molecules-1595698-supplementary.pdf]

Supplementary Information

Synthesis and Structure-activity Relationship Studies of Benzimidazole-4,7-dione-based P2X3 Receptor Antagonists as Novel Anti-nociceptive Agents

Jinsu Bae[1], Yeo Ok Kim[2], Xuehao Han[2], Myung Ha Yoon[2], Woong Mo Kim[2,*] and Yong-Chul Kim[1,3,*]

** Previously reported compounds, 10a-10b[18], 11a-11b[17] were reported by mass spectroscopy data according to the Instructions for Authors of MDPI.

6-chloro-5-((4-fluorophenyl)amino)-2-methyl-1H-benzo[d]imidazole-4,7-dione (10a)[18] Following the general procedure for the synthesis of **10a-b**, the substitution reaction of **9a** with 4-fluoroaniline afforded **10a**. red purple powder. Yield 67%; ^{1}H NMR (400 MHz, DMSO-d6) δ ppm 2.32 (s, 4 H) 7.03 - 7.16 (m, 4 H); LC/MS (ESI, m/z) 303.7 $[M - H]^-$ 305.8 $[M + H]^+$.

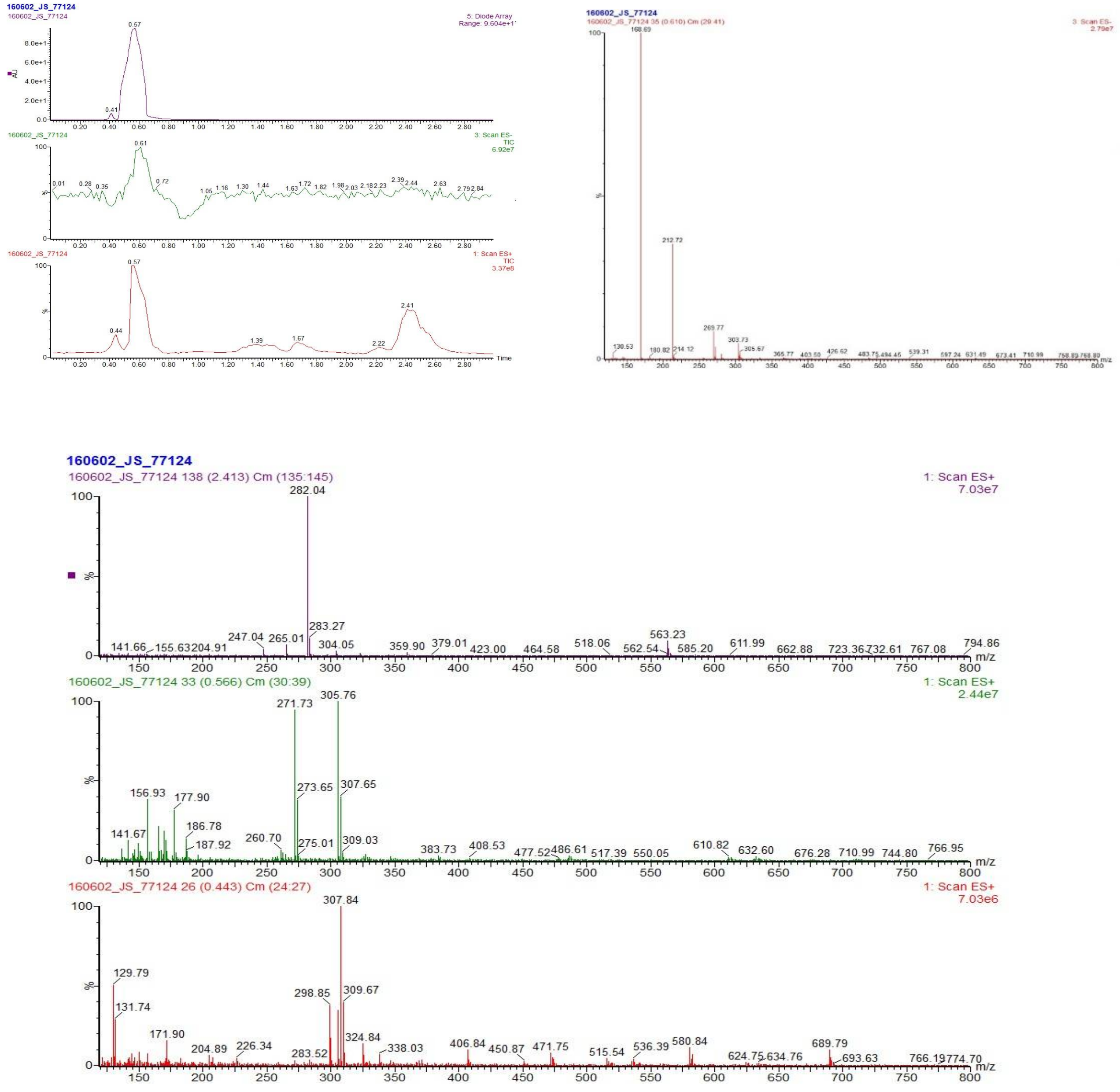

Figure S1. LC/MS spectrum of compound 10a

6-chloro-5-((4-chlorophenyl)amino)-2-methyl-1H-benzo[d]imidazole-4,7-dione (10b)[18] Following the general procedure for the synthesis of **10a-b**, the substitution reaction of **9a** with 4-chloroaniline afforded **10b**. red purple powder. Yield 71%; ^{1}H NMR (400 MHz, DMSO-d6) δ ppm 13.6 (s, 1H, NH), 9.0 (s, 1H, NH), 7.0–7.3 (dd, J = 2.4, 8.4 Hz, 4H, Ph-H), 2.2 (s, 3H, CH3); LC/MS (ESI, m/z) 319.8 [M - H]$^-$ 321.8 [M + H]$^+$.

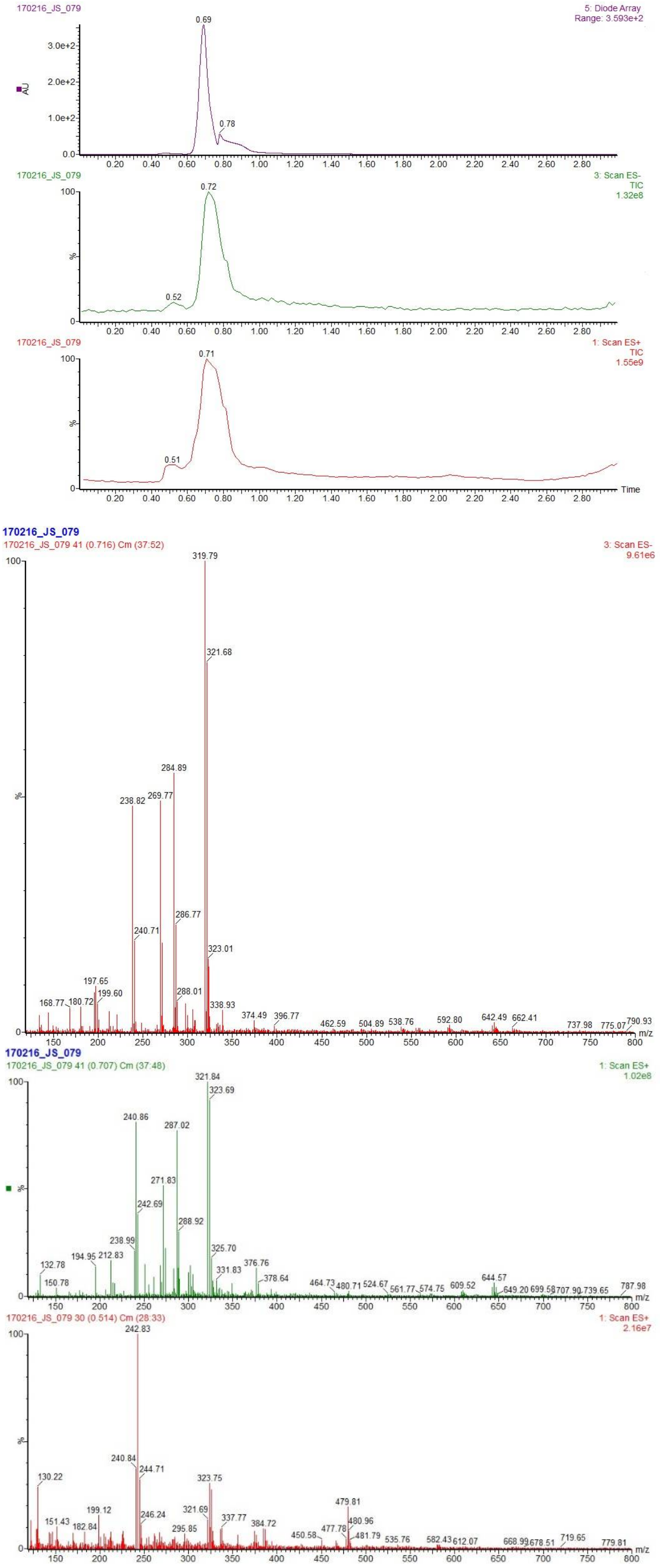

Figure S2. LC/MS spectrum of compound 10b

6-chloro-5-((4-fluorophenyl)amino)-2-(trifluoromethyl)-1H-benzo[d]imidazole-4,7-dione (11a)(17) Following the general procedure for the synthesis of **11a-k**, the substitution reaction of **9b** with 4-fluoroaniline afforded **11a**. purple powder. Yield 41%; ^{1}H NMR (400 MHz, METHANOL-d4) δ ppm 6.98 - 7.13 (m, 4 H); LC/MS (ESI, m/z) 358.3 [M - H]$^-$ 360.0 [M + H]$^+$.

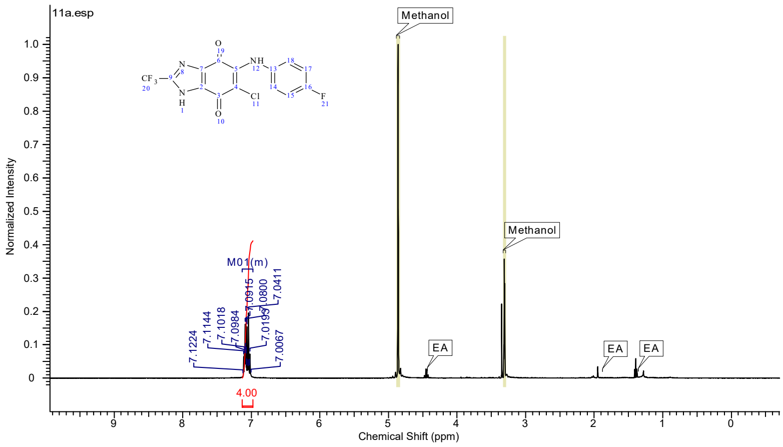

Figure S3. 1D 1H NMR spectrum of compound 11a

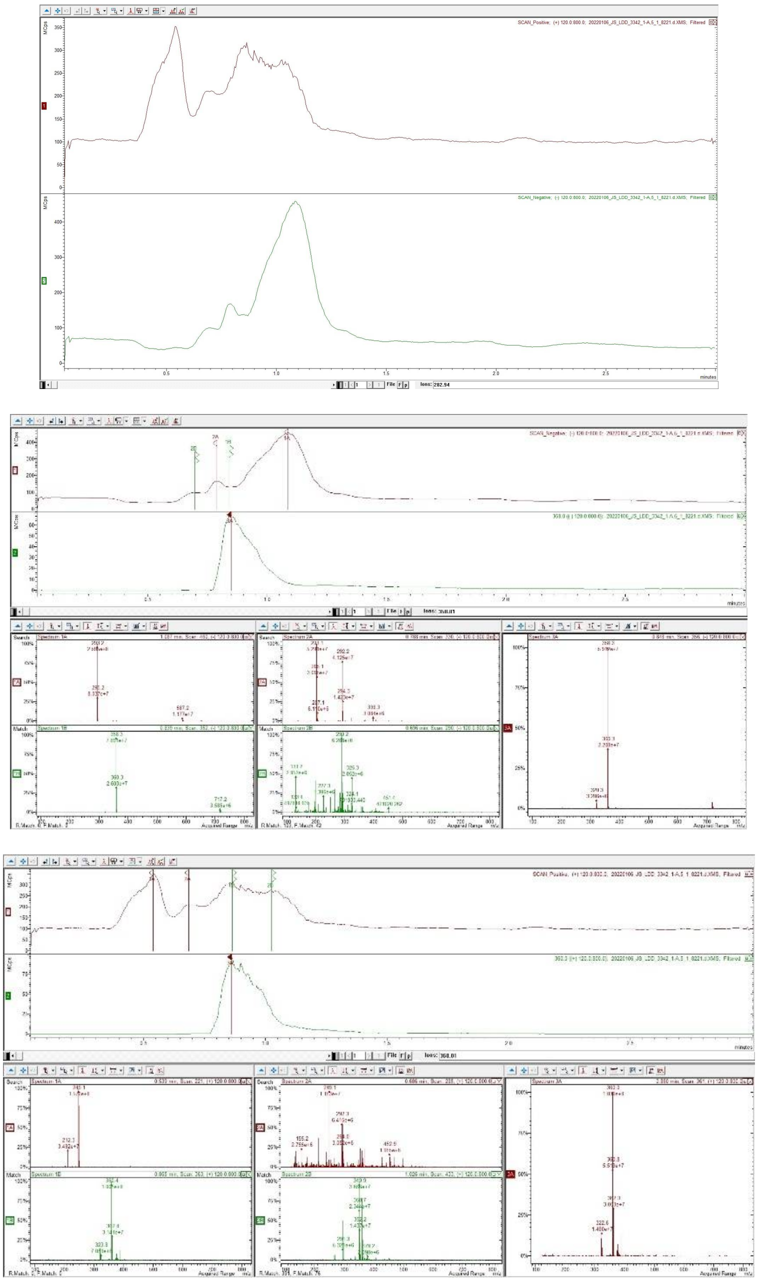

Figure S4. LC/MS spectrum of compound 11a

6-chloro-5-((4-chlorophenyl)amino)-2-(trifluoromethyl)-1H-benzo[d]imidazole-4,7-dione (11b)[17] Following the general procedure for the synthesis of **11a-k**, the substitution reaction of **9b** with 4-chloroaniline afforded **11b**. purple powder. Yield 56%; ^{1}H NMR (400 MHz, METHANOL-d4) δ ppm 7.01 - 7.10 (m, 2 H) 7.23 - 7.31 (m, 2 H); LC/MS (ESI, m/z) 374.3 [M - H]$^-$ 375.8 [M + H]$^+$.

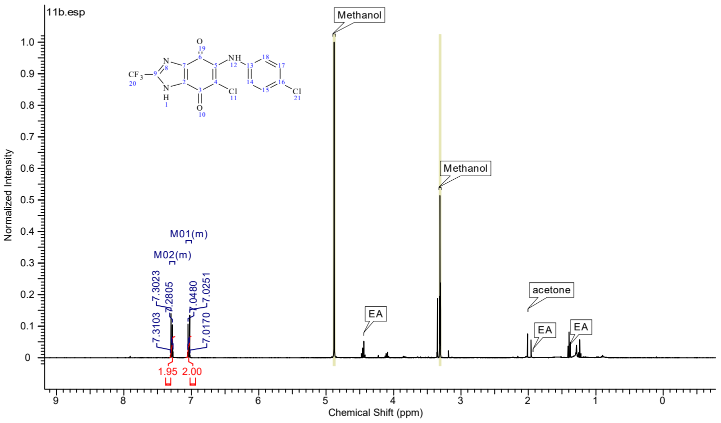

Figure S5. 1D 1H NMR spectrum of compound 11b

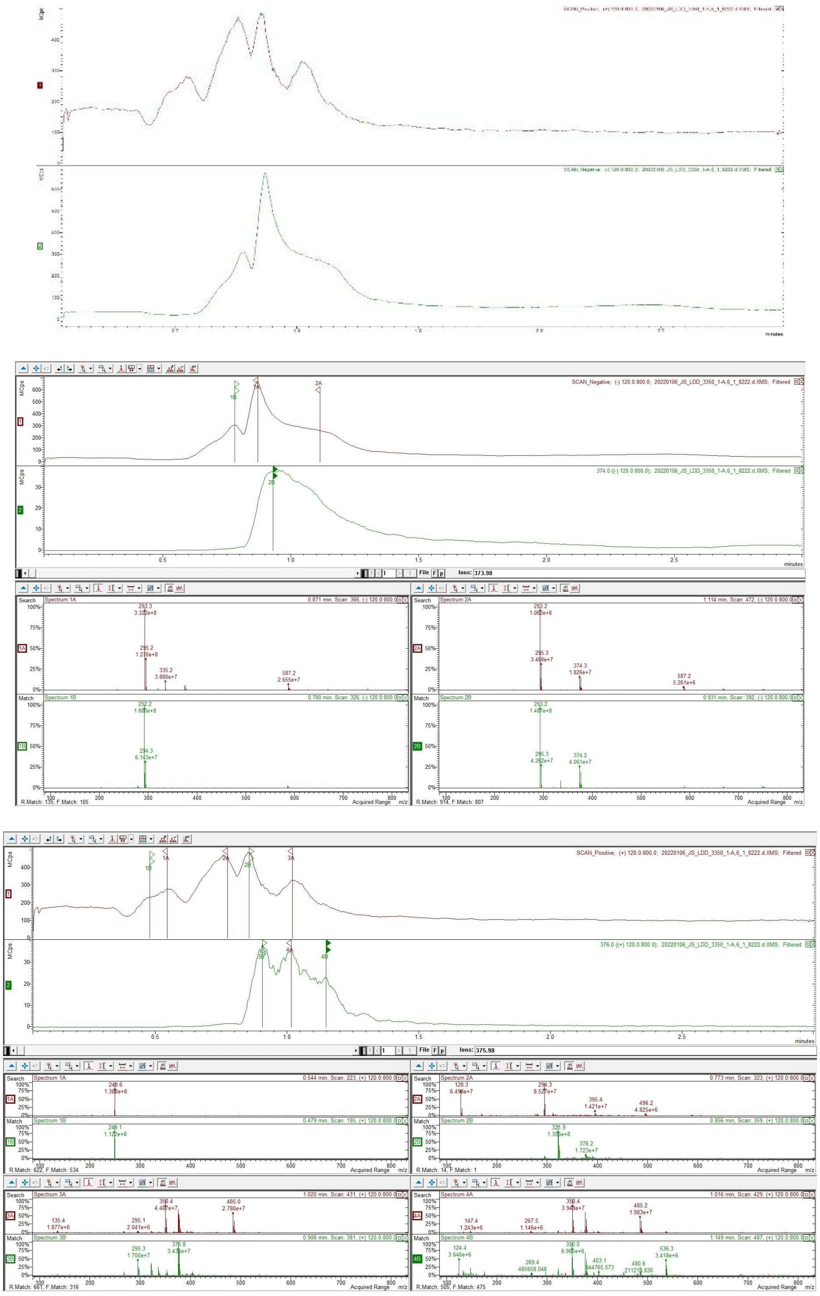

Figure S6. LC/MS spectrum of compound 11b

6-chloro-5-((3,4-difluorophenyl)amino)-2-(trifluoromethyl)-1H-benzo[d]imidazole-4,7-dione (11c) Following the general procedure for the synthesis of **11a-k**, the substitution reaction of **9b** with 3,4-difluoroaniline afforded **11c**. dark red powder. **11c** fully dissolved in 600 μL of the acetone-d6 and few drops of methanol. Yield 31%; ^{1}H NMR (400 MHz, ACETONE-d6) δ ppm 7.00 - 7.07 (m, 1 H) 7.20 (ddd, J=11.91, 7.33, 2.75 Hz, 1 H) 7.24 - 7.34 (m, 1 H) 8.51 (s, 1 H); ^{13}C NMR (100 MHz, ACETONE-d6) δ 174.18, 173.99, 142.43, 141.55, 137.99, 135.97, 135.86, 121.28, 120.73, 118.60, 116.57, 116.39, 113.53, 113.33, 111.59, 145~155 ppm peaks are considered as impurities; LC/MS (ESI, m/z) 376.3 [M - H]$^{-}$ 377.1 [M + H]$^{+}$.

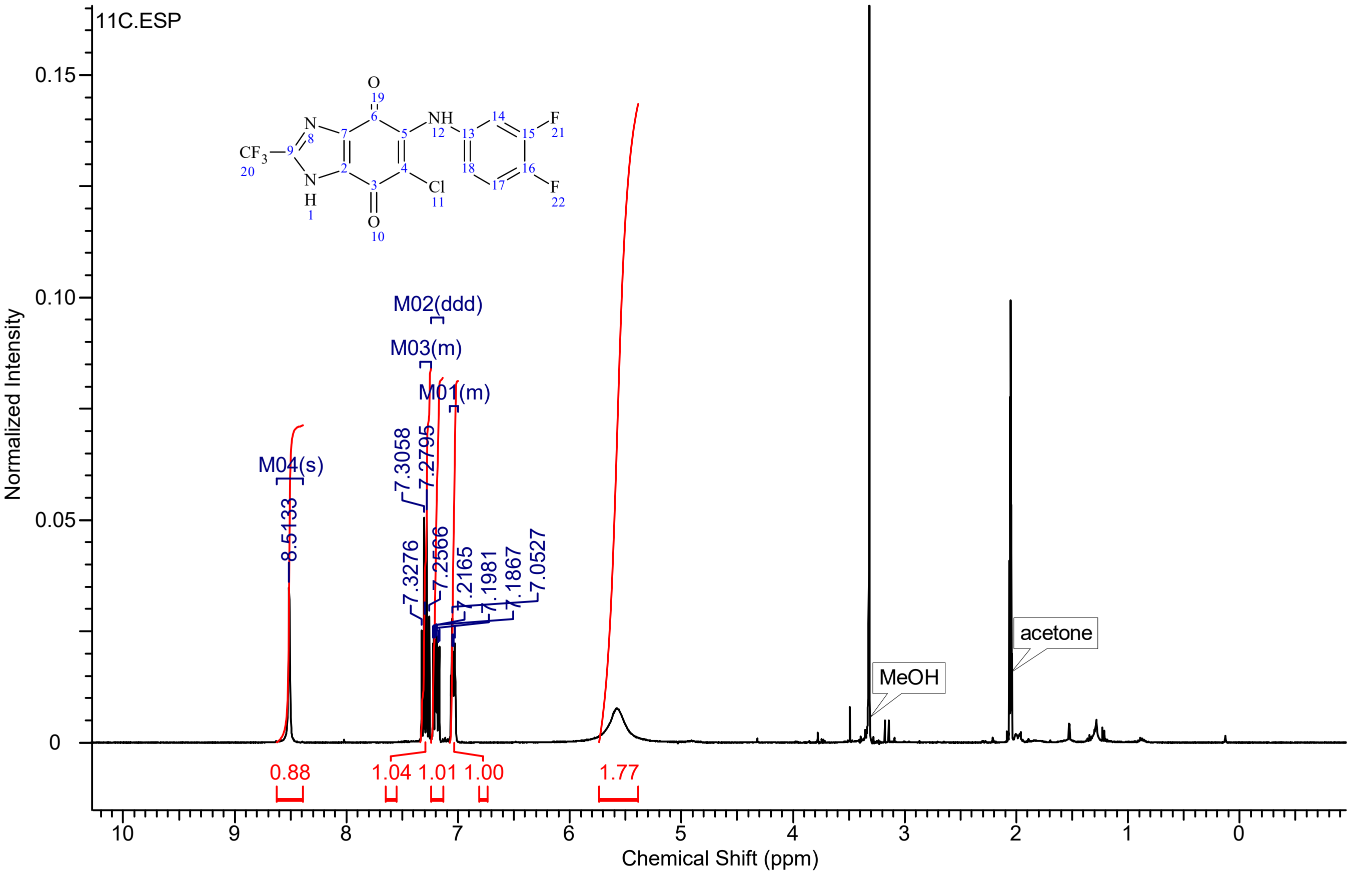

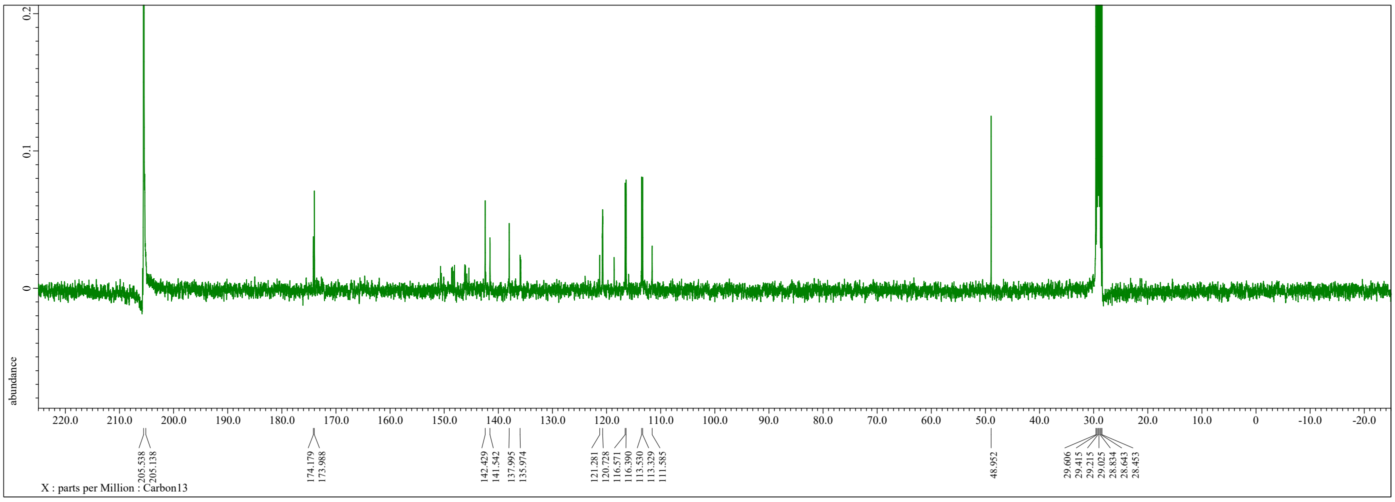

Figure S7. 1D 1H and 13C NMR spectrum of compound 11c

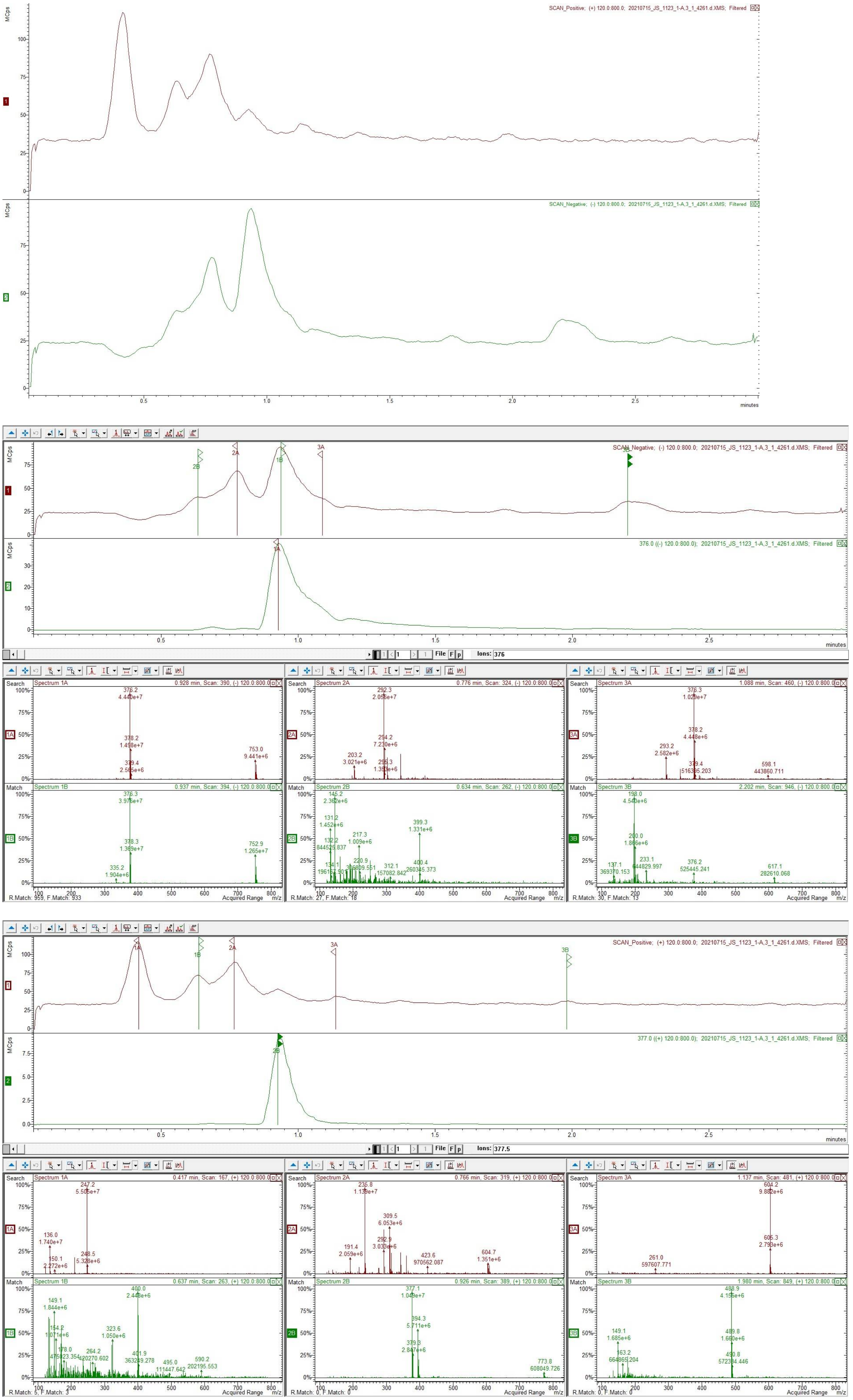

Figure S8. LC/MS spectrum of compound 11c

6-chloro-5-((3,4-dichlorophenyl)amino)-2-(trifluoromethyl)-1H-benzo[d]imidazole-4,7-dione (11d) Following the general procedure for the synthesis of **11a-k**, the substitution reaction of **9b** with 3,4-dichloroaniline afforded **11d**. red purple powder. **11d** fully dissolved in 600 μL of the methanol-d4 and few drops of acetone. Yield 38%; ^{1}H NMR (400 MHz, METHANOL-d4) δ ppm 7.01 (dd, J=8.47, 2.52 Hz, 1 H) 7.24 (d, J=2.29 Hz, 1 H) 7.43 (d, J=8.70 Hz, 1 H); ^{13}C NMR (100 MHz, METHANOL-d4) δ 174.16, 173.42, 142.22, 139.94, 139.24, 137.54, 131.91, 129.96, 127.88, 125.59, 123.75, 120.98, 113.37, 2 carbon peaks in 125.59 ppm overlapped; LC/MS (ESI, m/z) 408.0 [M - H]$^{-}$ 410.2 [M + H]$^{+}$.

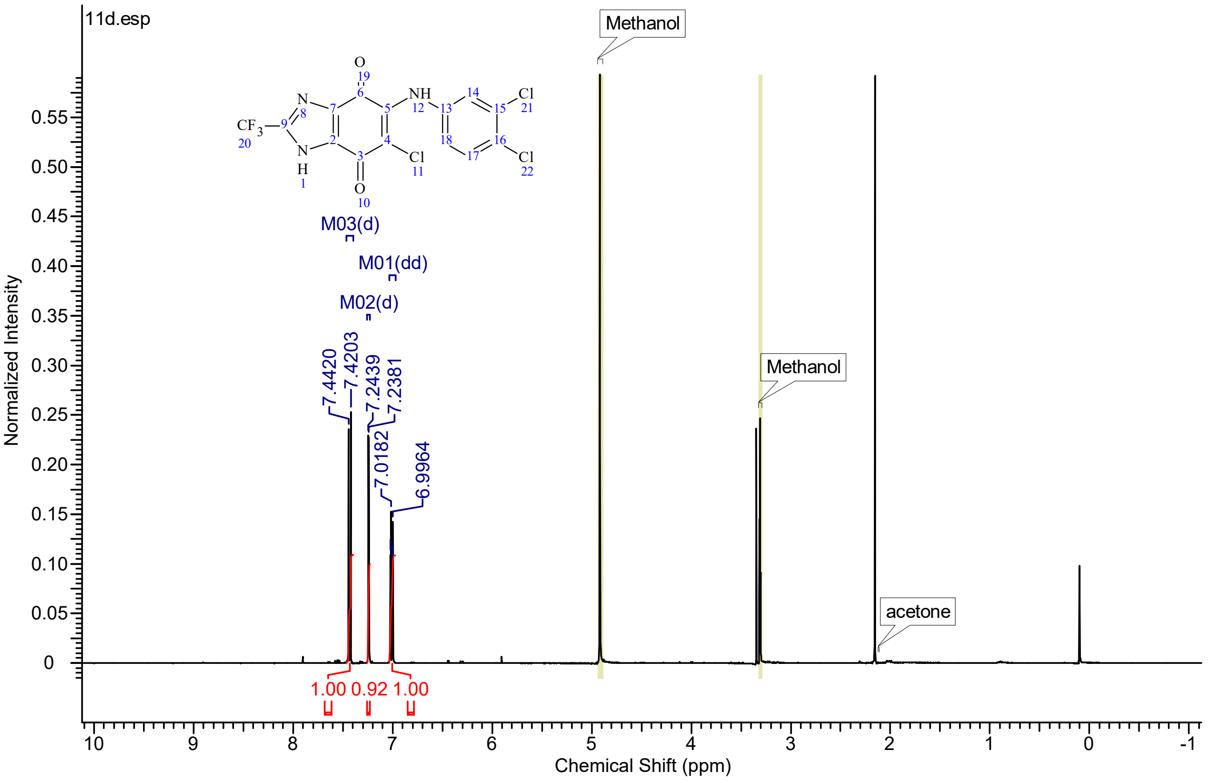

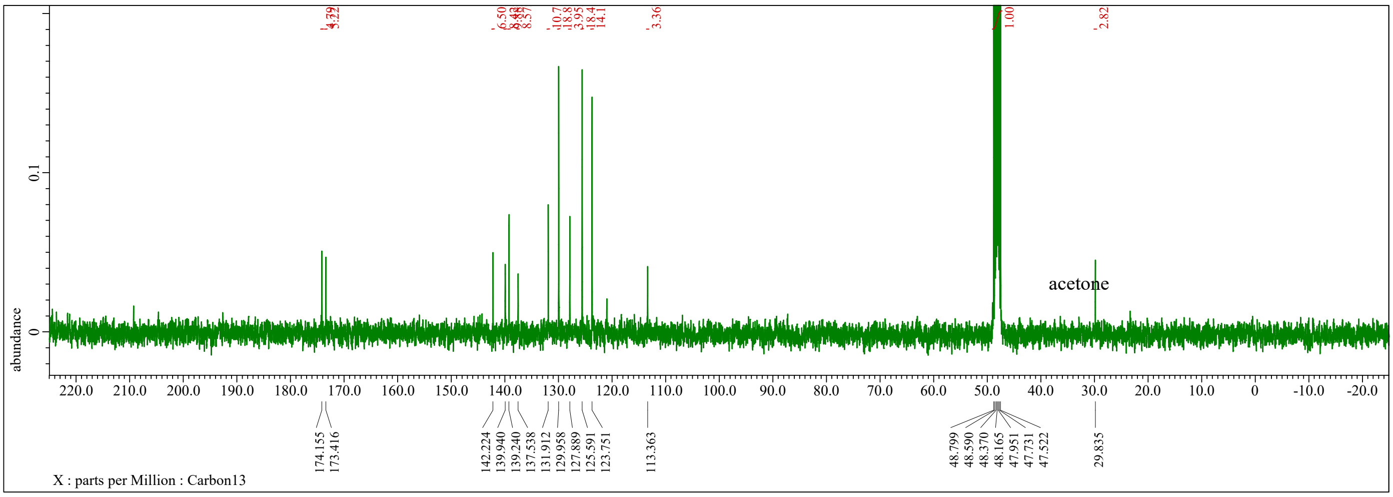

Figure S9. 1D 1H and 13C NMR spectrum of compound 11d

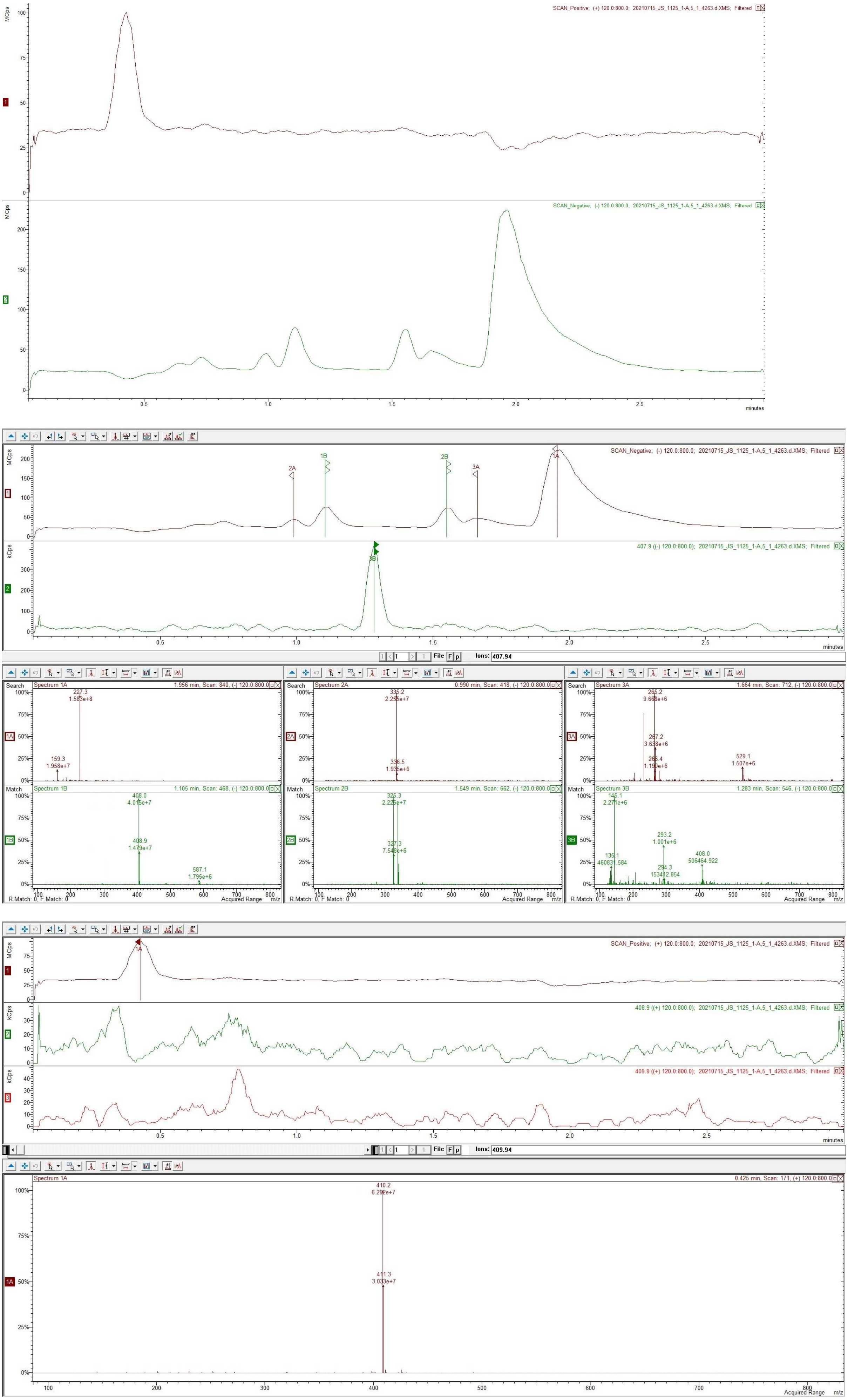

Figure S10. LC/MS spectrum of compound 11d

6-chloro-5-((2-chloro-4-fluorophenyl)amino)-2-(trifluoromethyl)-1H-benzo[d]imidazole-4,7-dione (11e) Following the general procedure for the synthesis of **11a-k**, the substitution reaction of **9b** with 2-chloro-4-fluoroaniline afforded **11e**. red purple powder. Yield 41%; ^{1}H NMR (400 MHz, METHANOL-d4) δ ppm 7.09 (td, J=8.47, 2.75 Hz, 1 H) 7.24 - 7.35 (m, 2 H); ^{13}C NMR (100 MHz, METHANOL-d4) δ 173.34, 173.26, 142.69, 140.09, 136.91, 132.87, 131.87, 131.76, 129.34, 120.64, 117.95, 116.14, 115.89, 113.73, 113.50, 109.94; LC/MS (ESI, m/z) 392.2 [M - H]$^{-}$ 394.1 [M + H]$^{+}$.

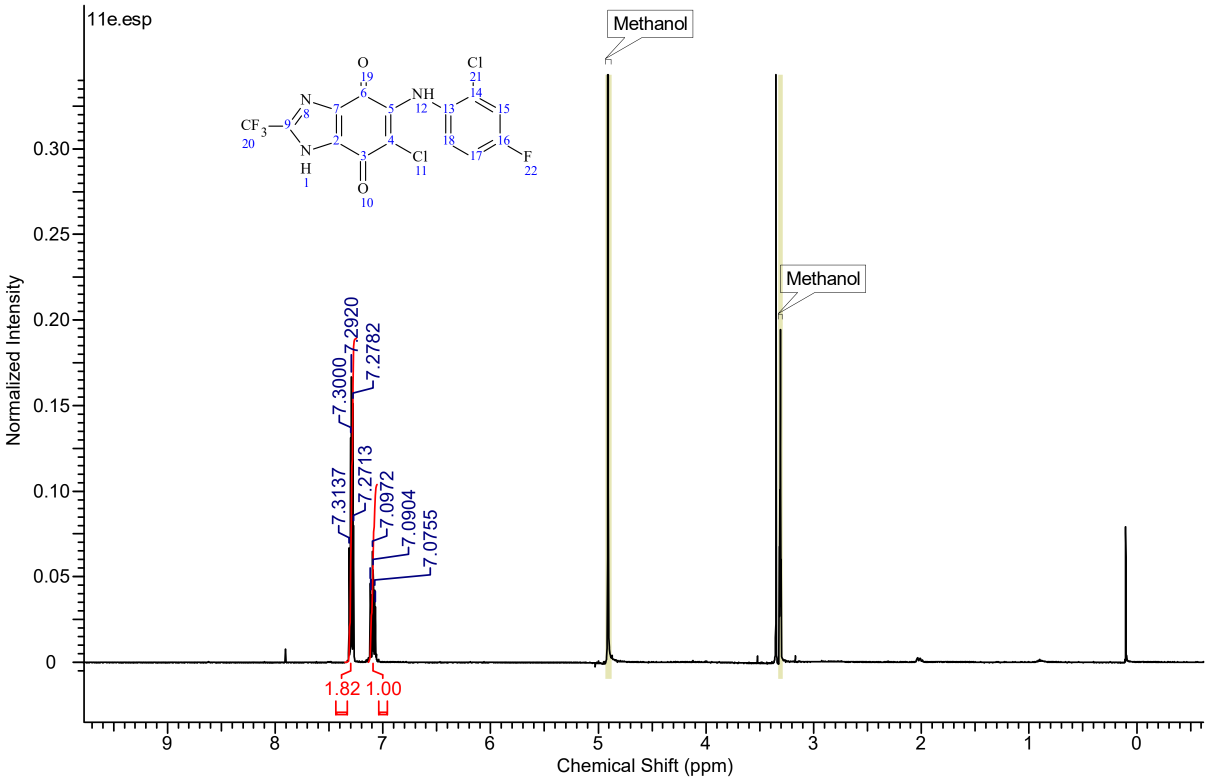

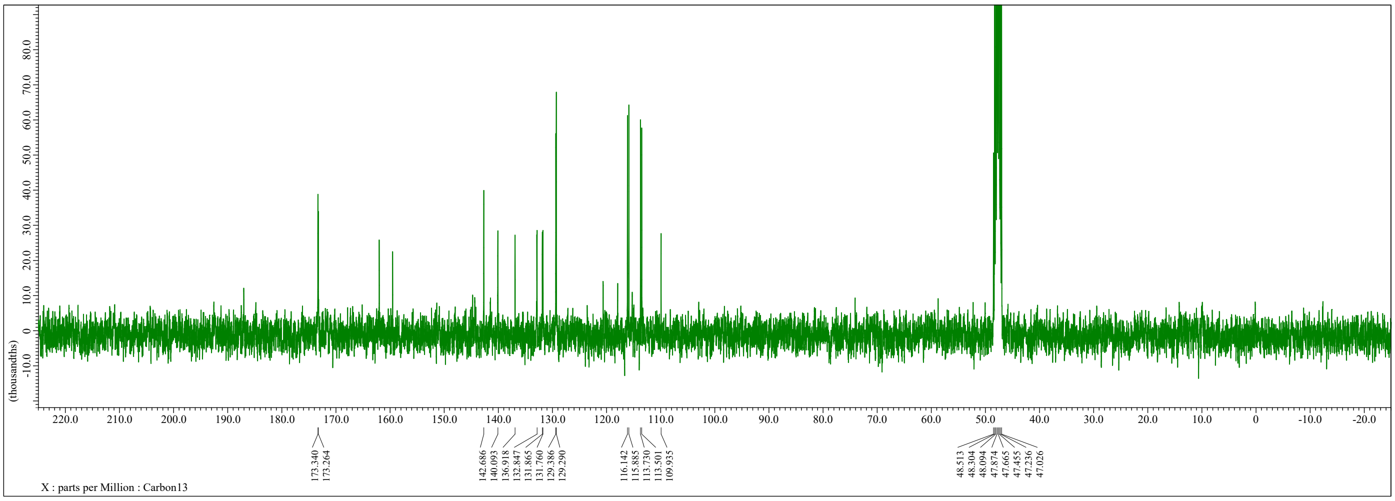

Figure S11. 1D 1H and 13C NMR spectrum of compound 11e

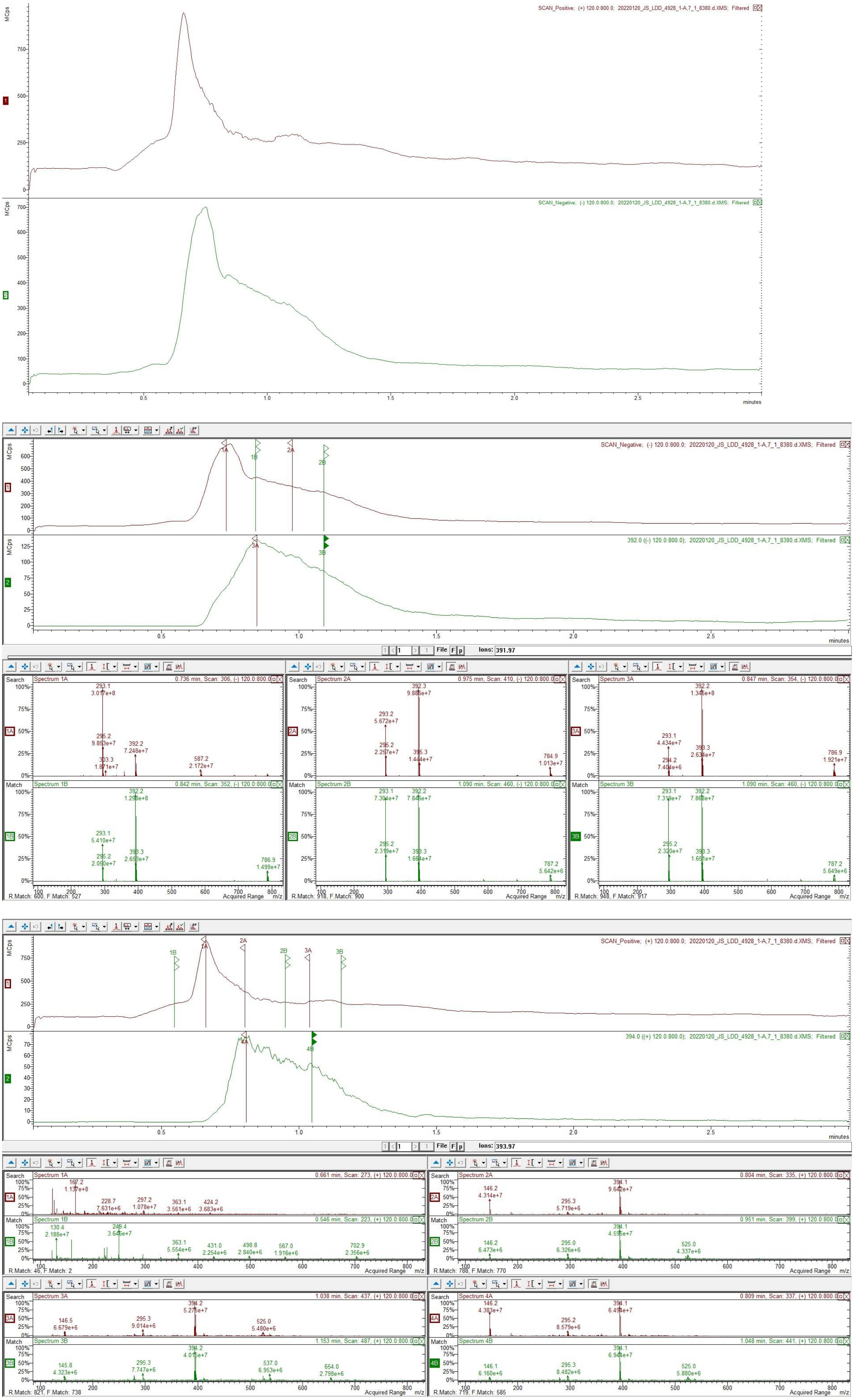

Figure S12. LC/MS spectrum of compound 11e

6-chloro-5-((3-chloro-4-fluorophenyl)amino)-2-(trifluoromethyl)-1H-benzo[d]imidazole-4,7-dione (11f) Following the general procedure for the synthesis of **11a-k**, the substitution reaction of **9b** with 3-chloro-4-fluoroaniline afforded **11f**. red purple powder. Yield 46%; ^{1}H NMR (400 MHz, METHANOL-d4) δ ppm 7.05 (ddd, J=8.93, 4.12, 2.52 Hz, 1 H) 7.13 - 7.24 (m, 2 H); ^{13}C NMR (100 MHz, METHANOL-d4) δ 178.81, 142.11, 140.72, 137.51, 135.81, 126.00, 124.28, 124.21, 119.86, 119.67, 115.65, 115.42, 111.30, 2 carbon peaks in 178.81 ppm overlapped, other peaks are impurities; LC/MS (ESI, m/z) 392.2 [M - H]$^{-}$ 393.7 [M + H]$^{+}$.

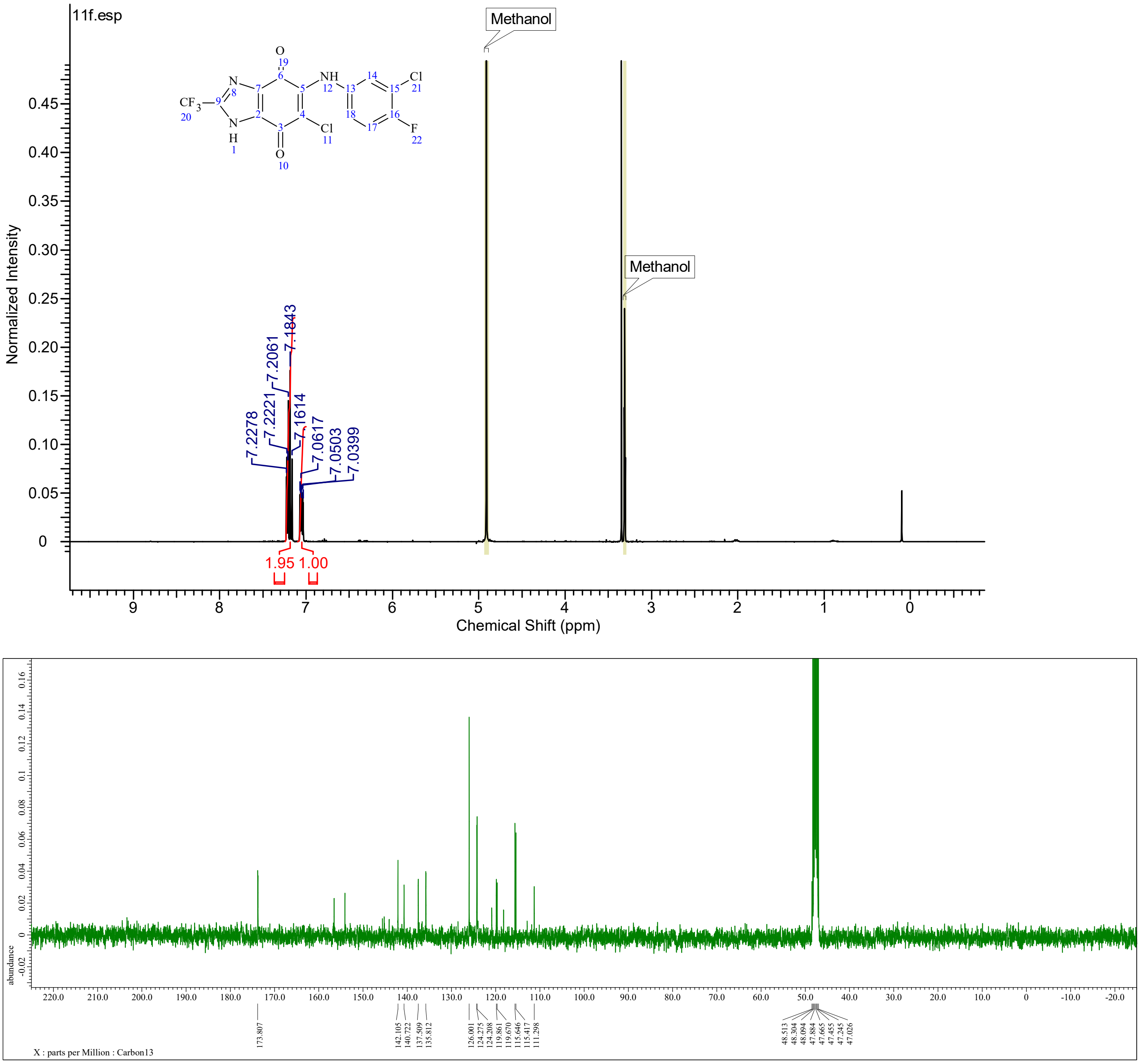

Figure S13. 1D 1H and 13C NMR spectrum of compound 11f

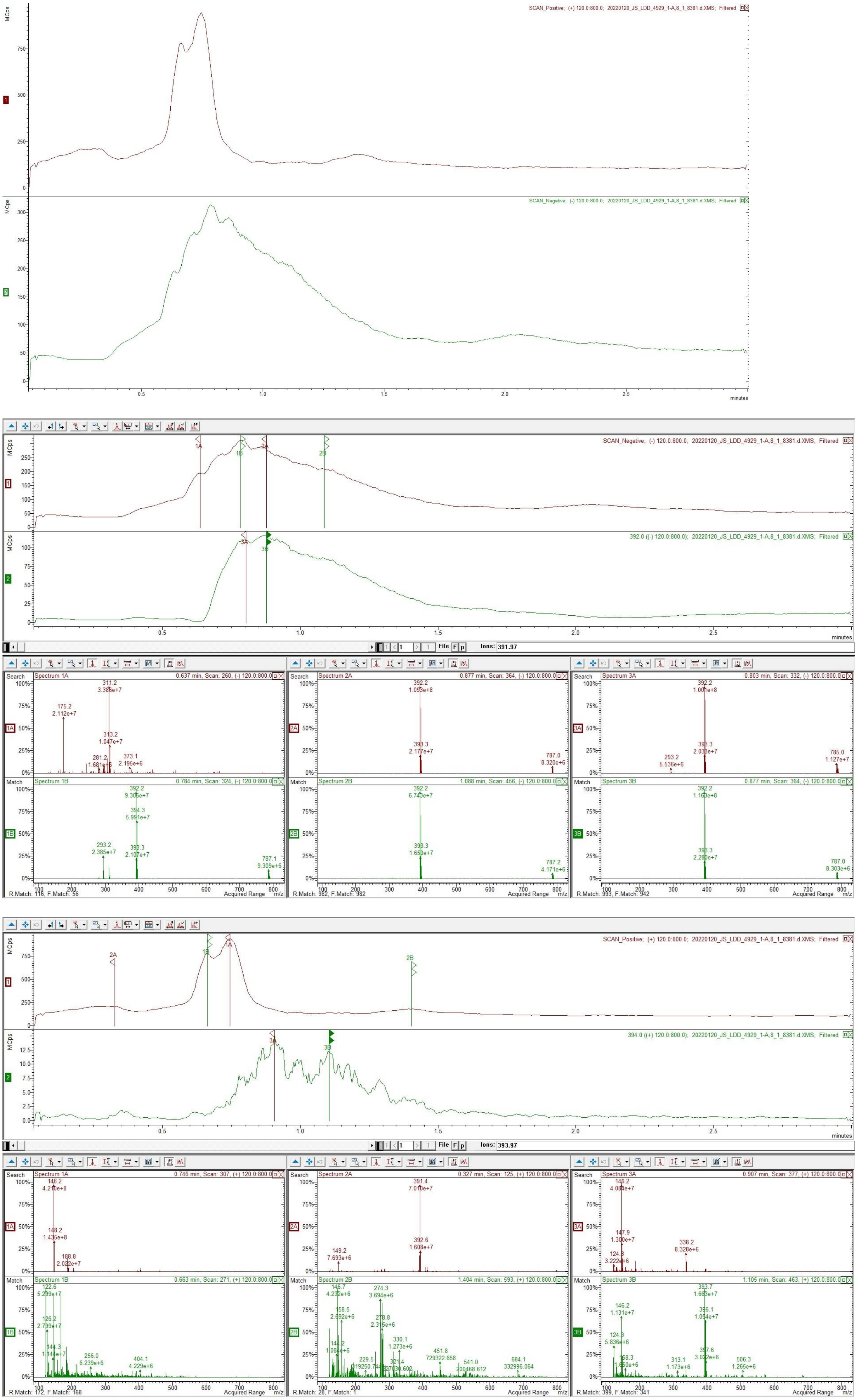

Figure S14. LC/MS spectrum of compound 11f

6-chloro-5-((4-chloro-2-fluorophenyl)amino)-2-(trifluoromethyl)-1H-benzo[d]imidazole-4,7-dione (11g) Following the general procedure for the synthesis of **11a-k**, the substitution reaction of **9b** with 4-chloro-2-fluoroaniline afforded **11g**. red purple powder. **11g** fully dissolved in 600 µL of the DMSO-d6 and few drops of acetone. Yield 52%; ^{1}H NMR (400 MHz, DMSO-d6) δ ppm 7.20 - 7.34 (m, 2 H) 7.44 (dd, J=10.30, 2.06 Hz, 1 H); ^{13}C NMR (100 MHz, DMSO-d6) δ 174.06, 173.44, 142.84, 142.73, 138.43, 130.20, 130.09, 128.65, 127.65, 127.53, 124.67, 116.48, 116.25, 111.93, peaks in 155 and 157 ppm are impurities; LC/MS (ESI, m/z) 392.2 [M - H]$^{-}$ 394.1 [M + H]$^{+}$.

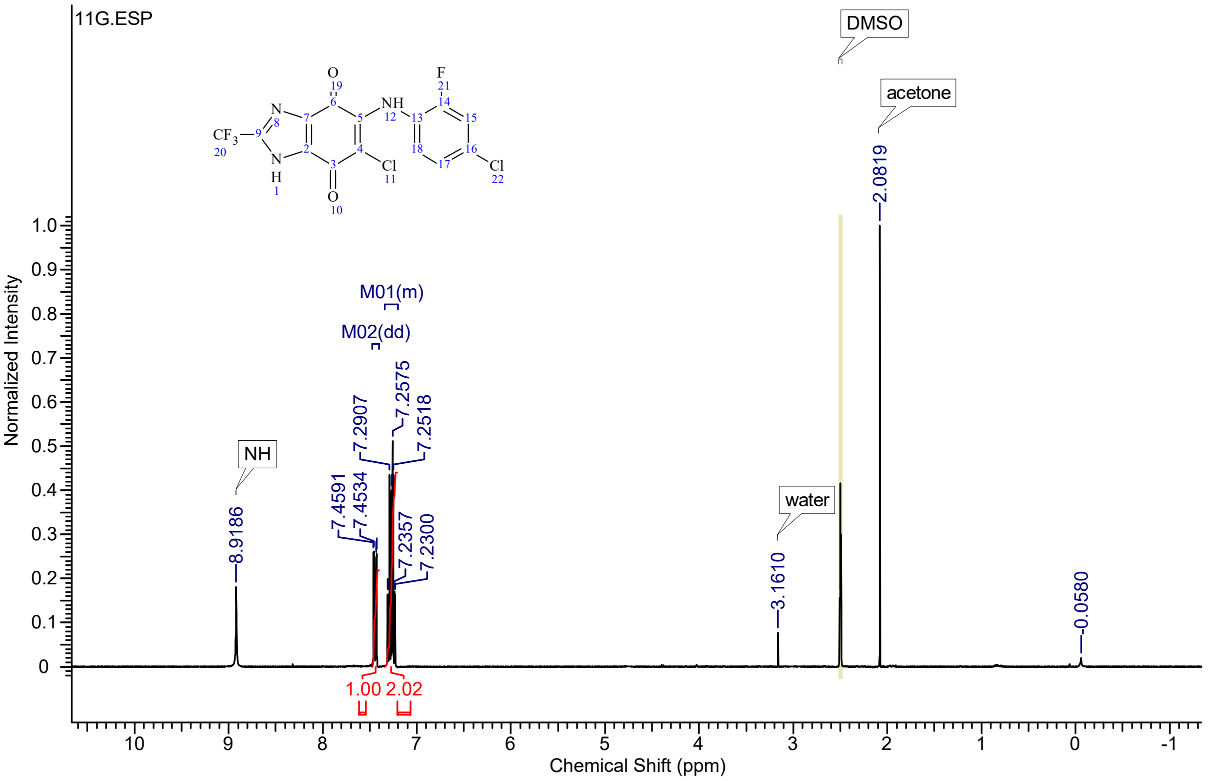

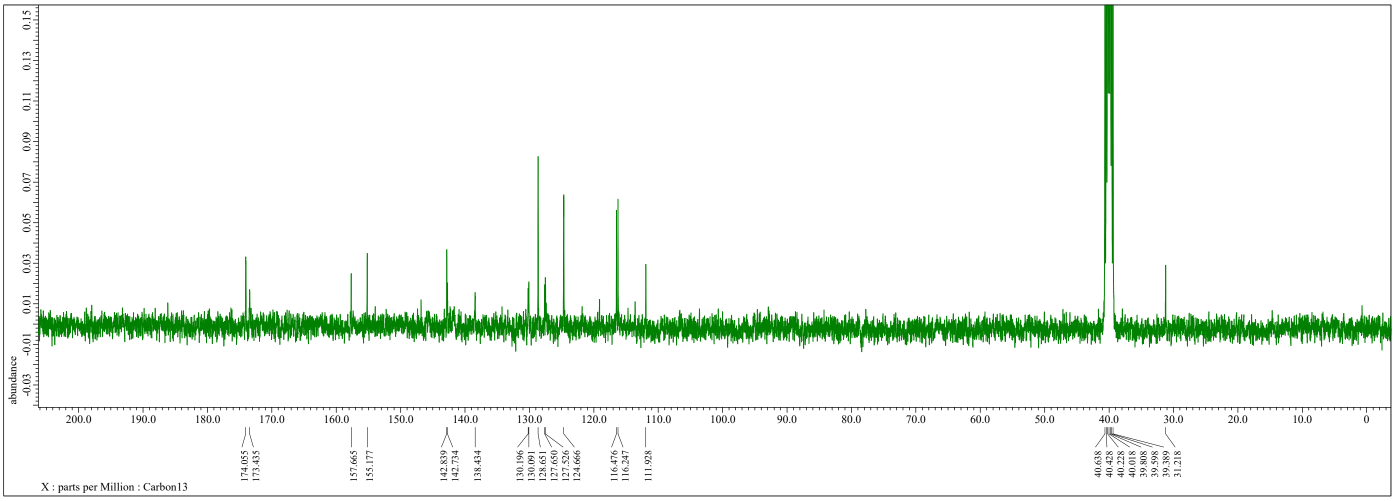

Figure S15. 1D 1H and 13C NMR spectrum of compound 11g

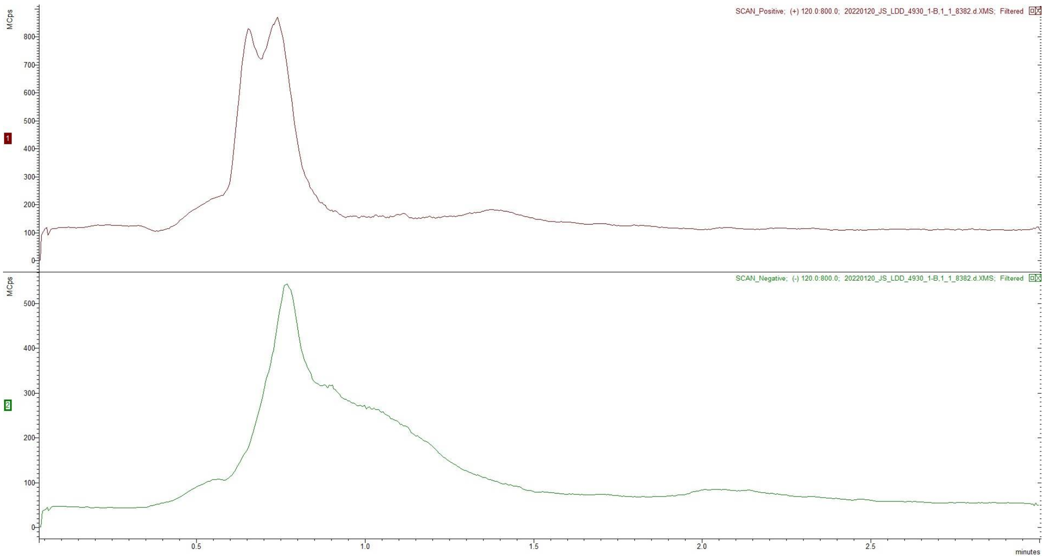

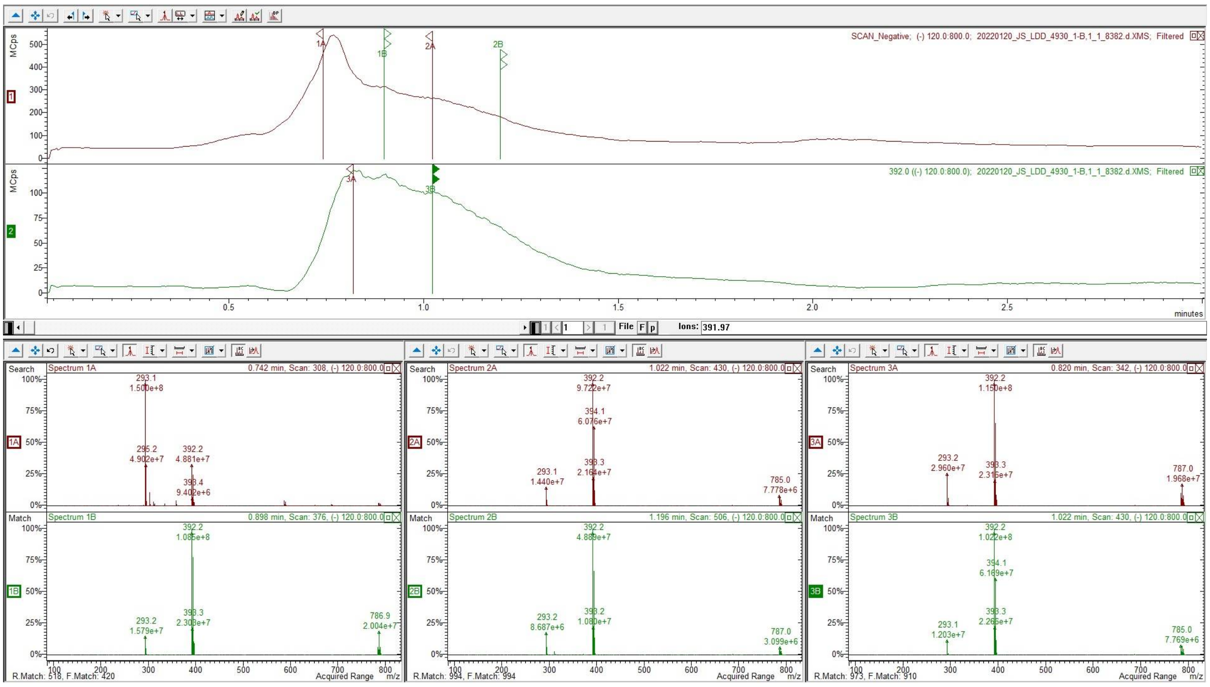

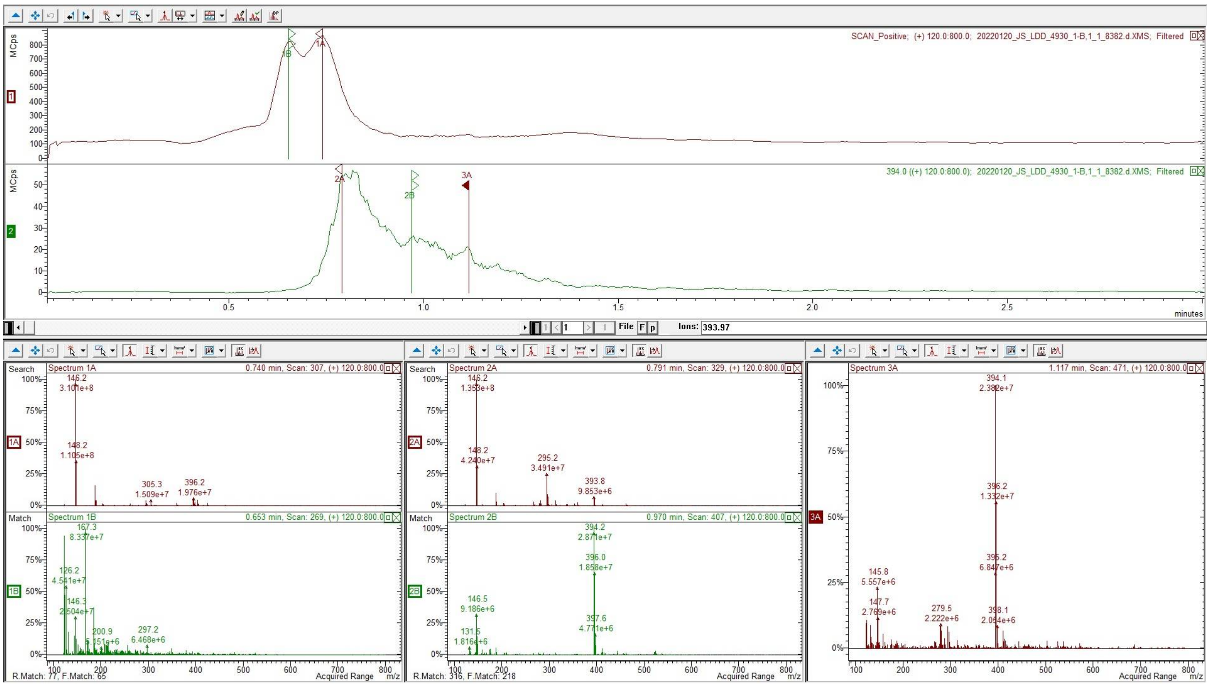

Figure S16. LC/MS spectrum of compound 11g

6-chloro-5-((4-chloro-3-fluorophenyl)amino)-2-(trifluoromethyl)-1H-benzo[d]imidazole-4,7-dione (11h) Following the general procedure for the synthesis of **11a-k**, the substitution reaction of **9b** with 4-chlro-3-fluoroaniline afforded **11h**. red purple powder. Yield 39%; ^{1}H NMR (400 MHz, METHANOL-d4) δ ppm 6.86 (dd, J=8.70, 0.92 Hz, 1 H) 6.89 - 6.98 (m, 1 H) 7.36 (t, J=8.47 Hz, 1 H); ^{13}C NMR (100 MHz, METHANOL-d4) δ 175.09, 174.29, 143.04, 141.47, 139.63, 139.05, 129.36, 119.91, 119.87, 115.26, 115.07, 113.25, 111.45, 111.21, peaks in 158.67 and 156.22 ppm are impurities; LC/MS (ESI, m/z) 392.2 [M - H]$^{-}$ 394.1 [M + H]$^{+}$.

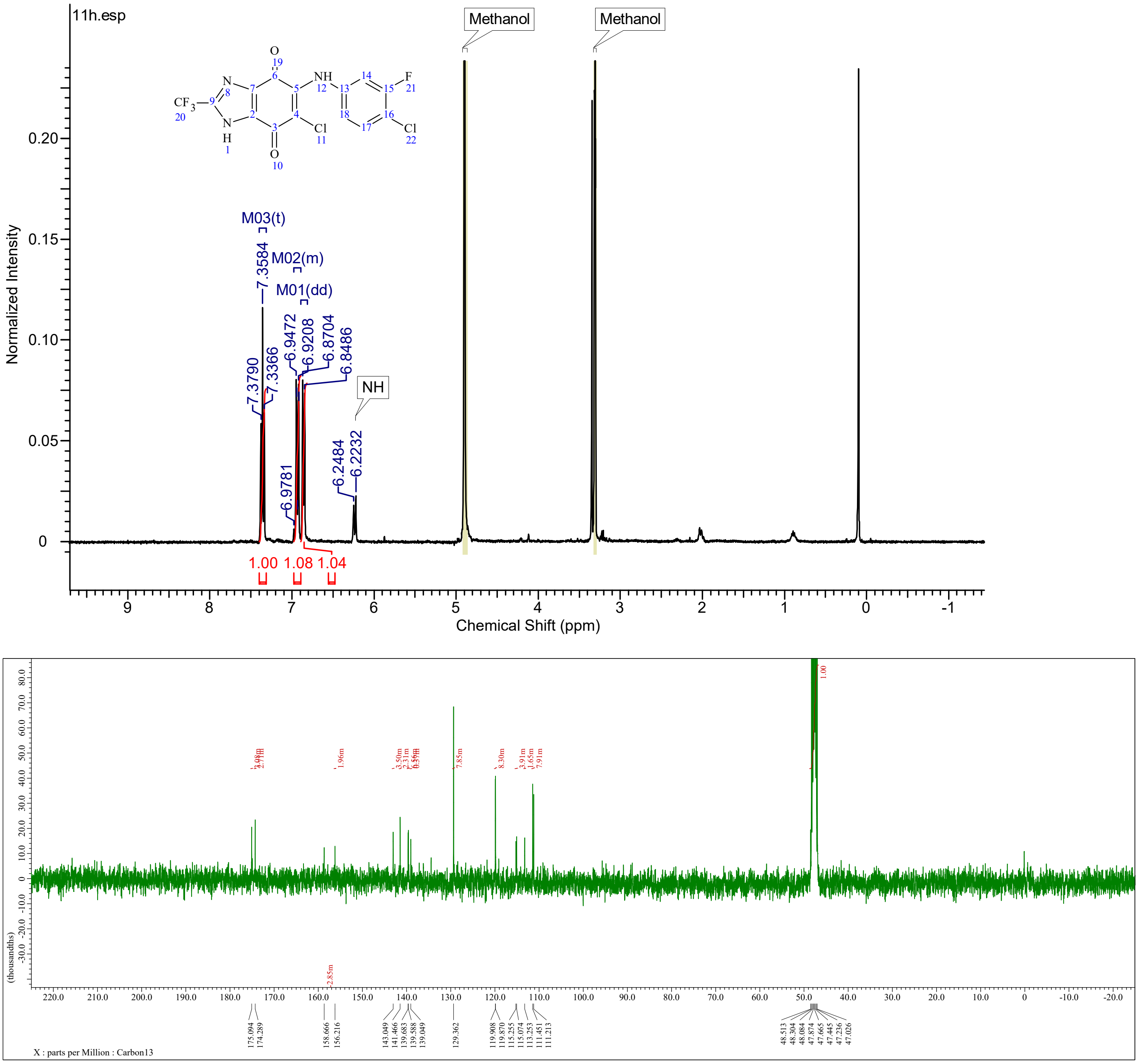

Figure S17. 1D 1H and 13C NMR spectrum of compound 11h

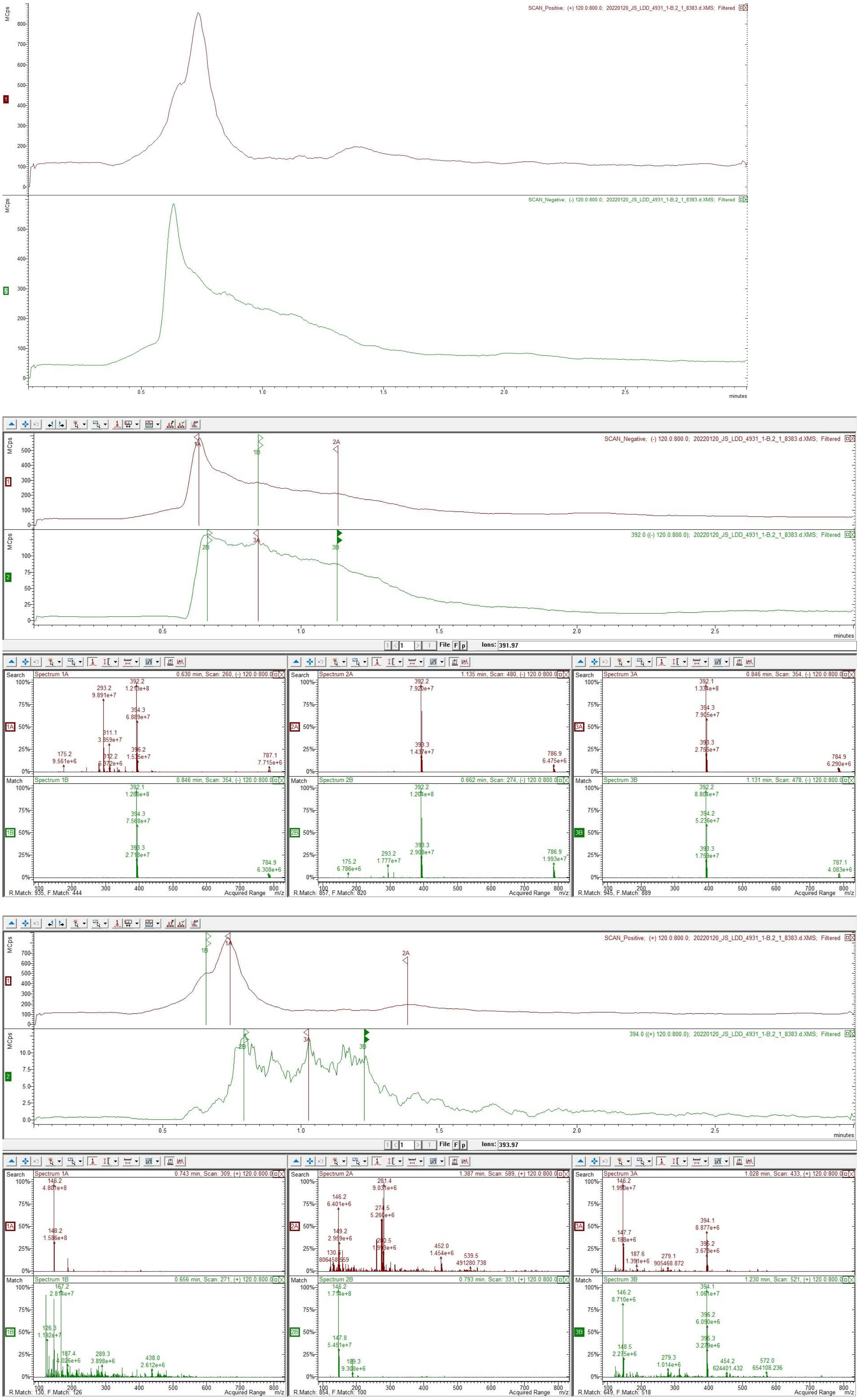

Figure S18. LC/MS spectrum of compound 11h

5-((3-bromo-4-fluorophenyl)amino)-6-chloro-2-(trifluoromethyl)-1H-benzo[d]imidazole-4,7-dione (11i) Following the general procedure for the synthesis of **11a-k**, the substitution reaction of **9b** with 3-bromo-4-fluoroaniline afforded **11i**. purple powder. Yield 63%; ^{1}H NMR (400 MHz, METHANOL-d4) δ ppm 7.06 - 7.11 (m, 1 H) 7.13 - 7.18 (m, 1 H) 7.34 (dd, J=5.95, 2.29 Hz, 1 H); ^{13}C NMR (100 MHz, METHANOL-d4) δ 174.43, 173.89, 142.12, 141.68, 137.99, 136.05, 128.83, 124.98, 124.90, 115.41, 115.17, 111.07, 107.54, 107.32, peaks in 157.57 and 155.14 ppm are impurities; LC/MS (ESI, m/z) 436.1 [M - H]$^{-}$ 437.8 [M + H]$^{+}$.

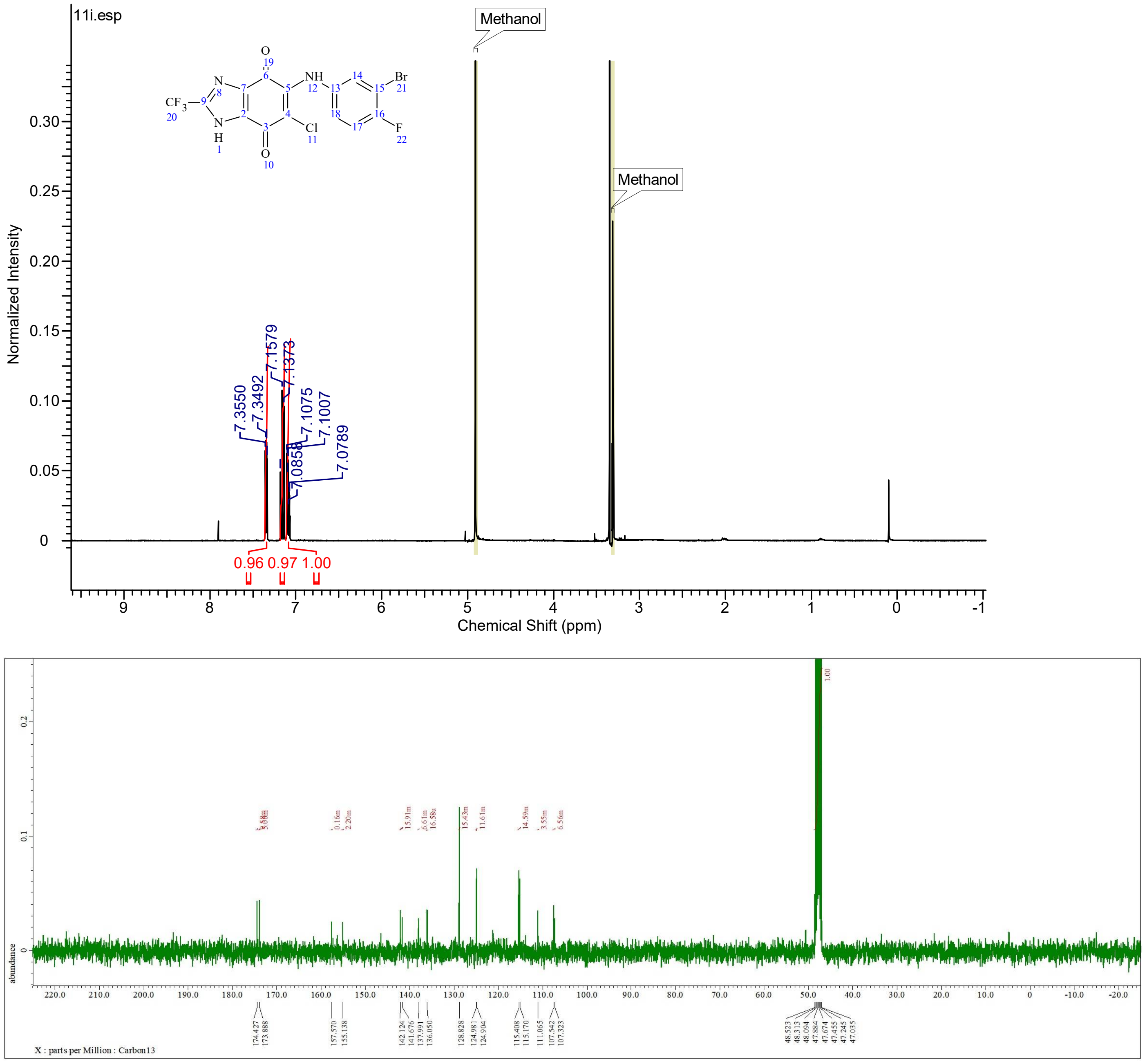

Figure S19. 1D 1H and 13C NMR spectrum of compound 11i

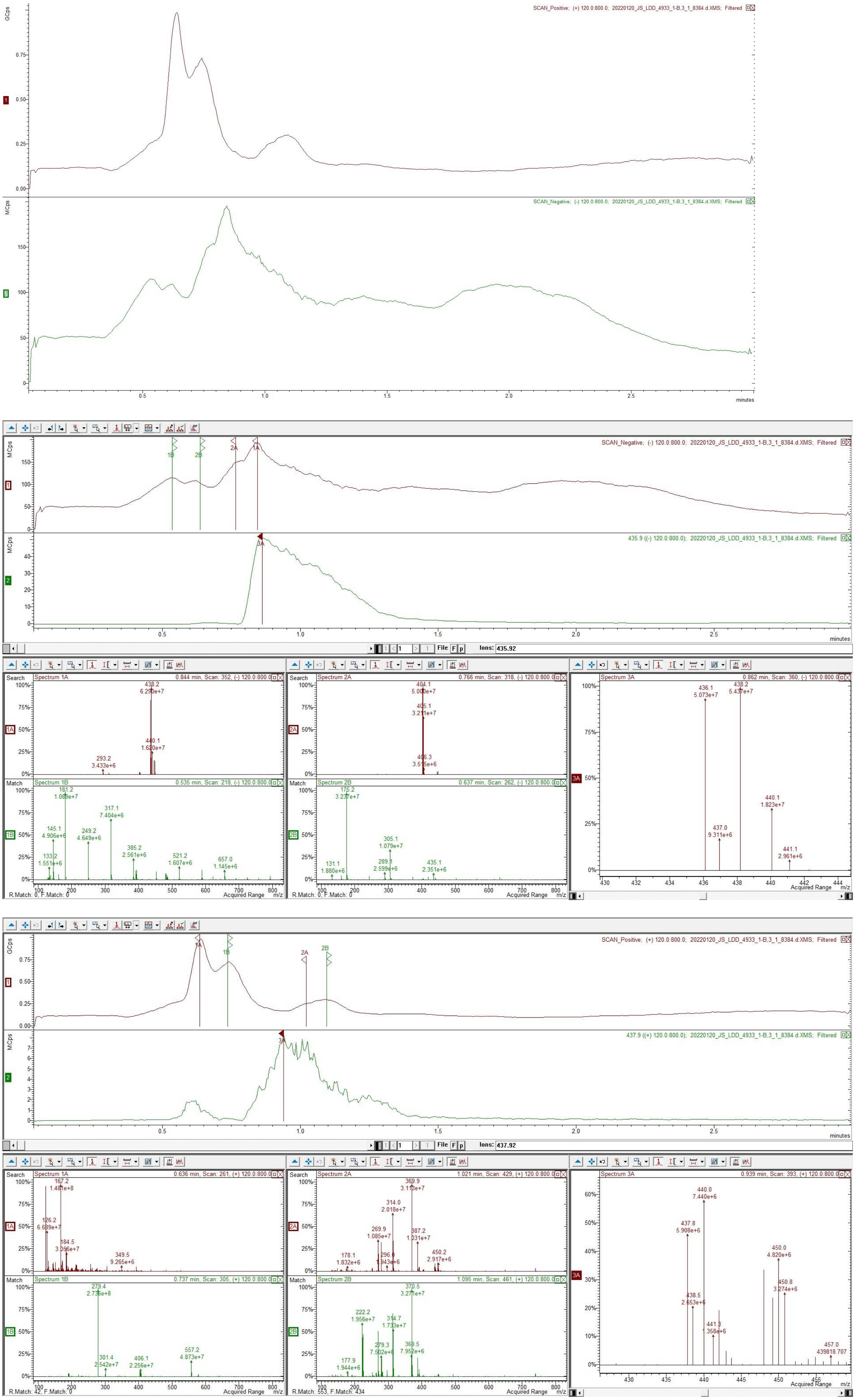

Figure S20. LC/MS spectrum of compound 11i

5-((4-bromo-3-fluorophenyl)amino)-6-chloro-2-(trifluoromethyl)-1H-benzo[d]imidazole-4,7-dione (11j) Following the general procedure for the synthesis of **11a-k**, the substitution reaction of **9b** with 4-bromo-3-fluoroaniline afforded **11j**. red purple powder. **11j** fully dissolved in 600 μL of the methanol-d4 and few drops of acetone. Yield 51%; ^{1}H NMR (400 MHz, METHANOL-d4) δ ppm 6.77 - 6.88 (m, 1 H) 6.94 (dd, J=10.08, 2.29 Hz, 1 H) 7.41 - 7.60 (m, 1 H); ^{13}C NMR (100 MHz, METHANOL-d4) δ 174.81, 174.27, 148.44, 148.06, 142.67, 141.38, 140.45, 140.35, 138.87, 132.27, 120.21, 113.56, 111.23, 110.98, peaks in 159.75, 157.32, and 102.54 ppm are impurities; LC/MS (ESI, m/z) 436.1 $[M - H]^{-}$ 438.0 $[M + H]^{+}$.

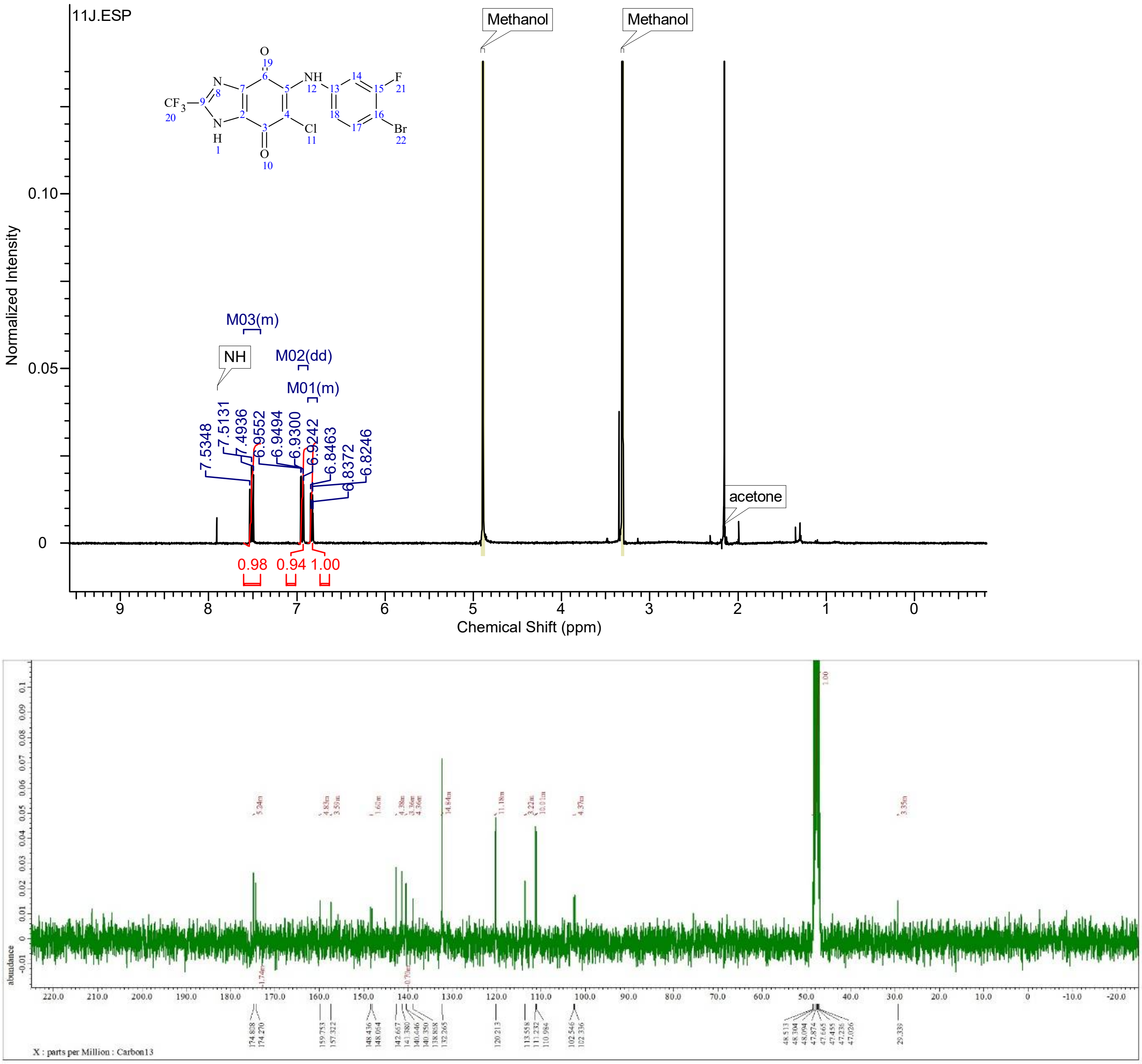

Figure S21. 1D 1H and 13C NMR spectrum of compound 11j

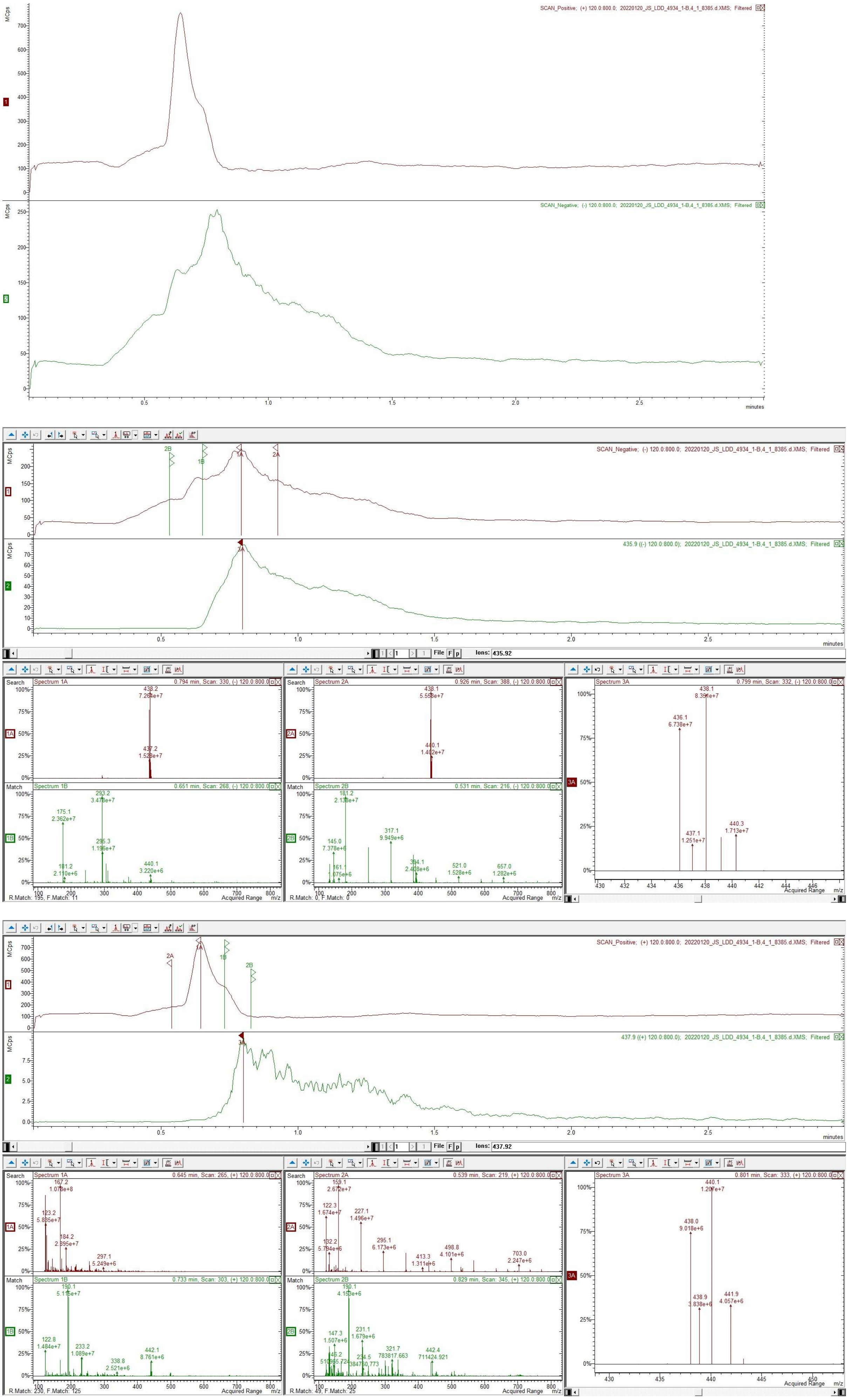

Figure S22. LC/MS spectrum of compound 11j

6-chloro-5-((4-chloro-2-iodophenyl)amino)-2-(trifluoromethyl)-1H-benzo[d]imidazole-4,7-dione (11k) Following the general procedure for the synthesis of **11a-k**, the substitution reaction of **9b** with 4-chloro-2-iodoaniline afforded **11k**. purple powder. **11k** fully dissolved in 600 μL of the methanol-d4 and few drops of acetone. Yield 24%; ^{1}H NMR (400 MHz, METHANOL-d4) δ ppm 7.14 (d, J=8.24 Hz, 1 H) 7.38 (d, J=10.99 Hz, 1 H) 7.88 (d, J=2.29 Hz, 1 H); ^{13}C NMR (100 MHz, METHANOL-d4) δ 175.75, 174.94, 141.80, 140.10, 138.43, 137.95, 131.35, 127.92, 127.78, 127.54, 125.09, 117.77, 110.53, 96.60, other peaks are impurities; LC/MS (ESI, m/z) 499.8 [M - H]$^-$ 501.9 [M + H]$^+$.

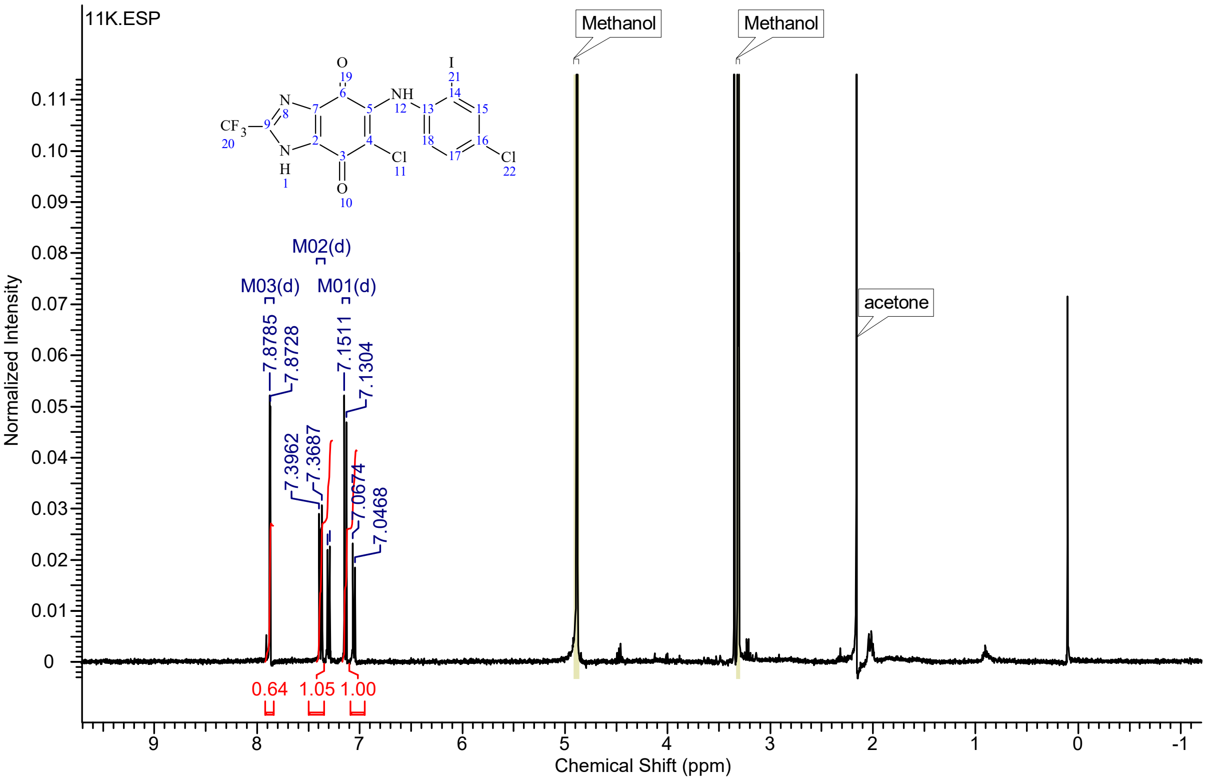

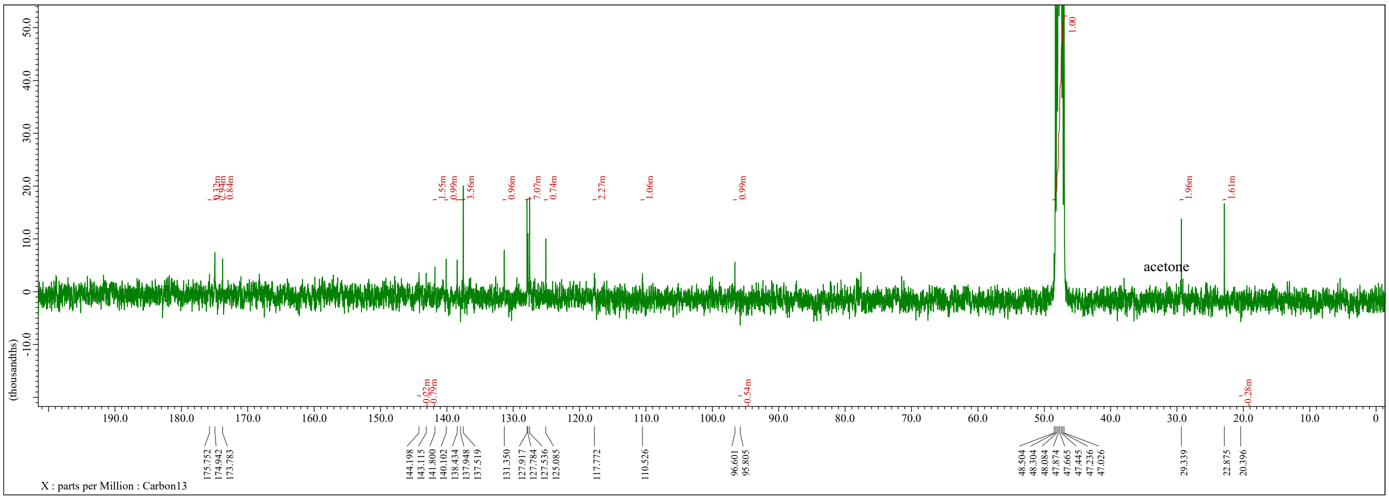

Figure S23. 1D 1H and 13C NMR spectrum of compound 11k

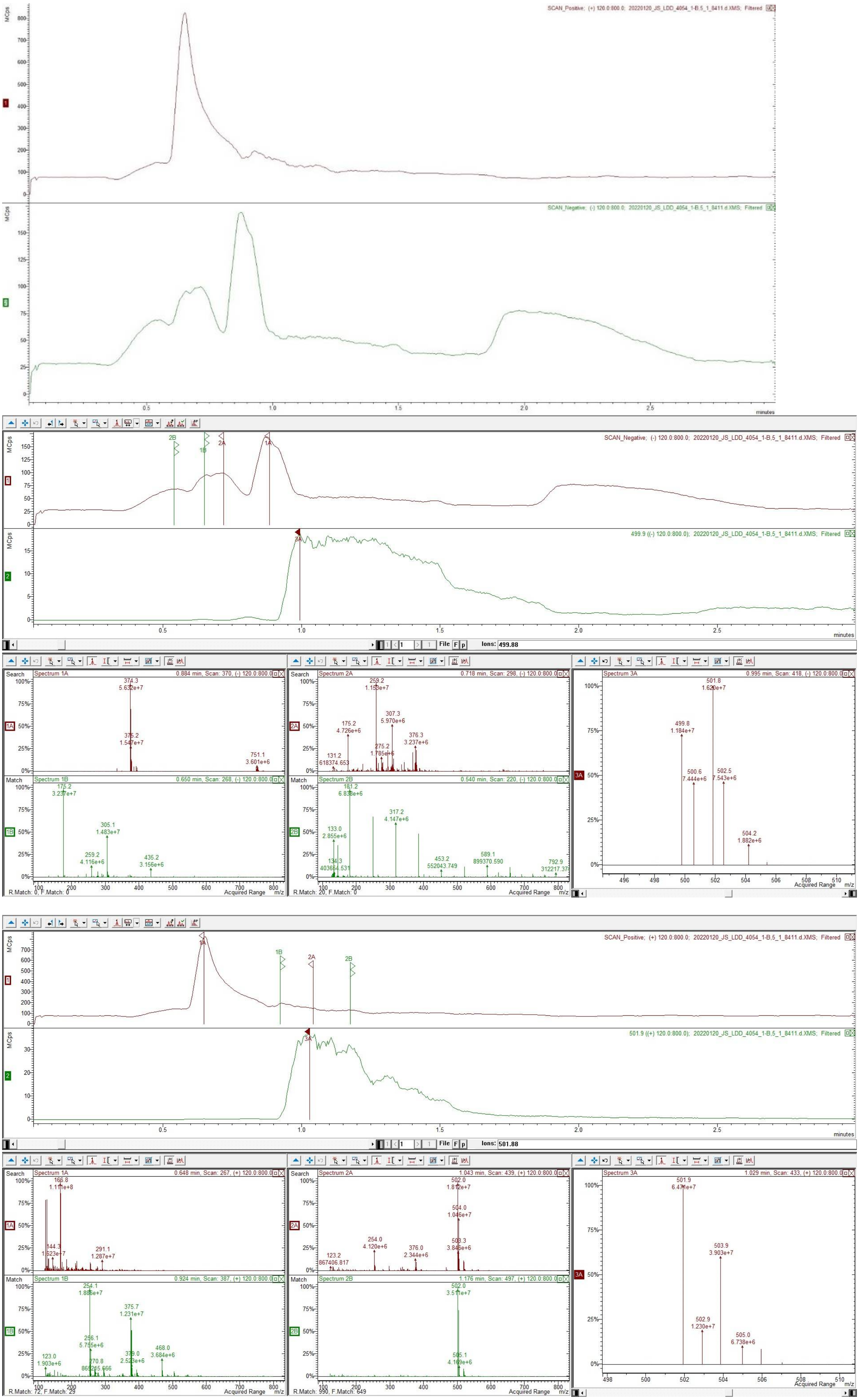

Figure S24. LC/MS spectrum of compound 11k

methyl-4-((6-chloro-4,7-dioxo-2-(trifluoromethyl)-4,7-dihydro-1H-benzo[d]imidazol-5-yl)amino)piperidine-1-carboxylate (14a) Following the general procedure for the synthesis of **14a-k**, the substitution reaction of **9b** with **13a** afforded **14a**. purple powder. **14a** fully dissolved in 600 μL of the methanol-d4 and a drop of acetone. Yield 37%; ^{1}H NMR (400 MHz, METHANOL-d4) δ ppm 1.52 (d, J=15.11 Hz, 2 H) 2.04 (br. s., 2 H) 3.00 (br. s., 2 H) 3.69 (s, 3 H) 4.09 (br. s., 2 H) 4.61 (s, 1 H); ^{13}C NMR (100 MHz, METHANOL-d4) δ 175.76, 173.83, 169.11, 156.24, 144.98, 143.53, 138.29, 122.12, 119.44, 51.97, 50.52, 42.29, 32.84, other peaks are impurities; LC/MS (ESI, m/z) 404.9 $[M - H]^{-}$ 406.8 $[M + H]^{+}$.

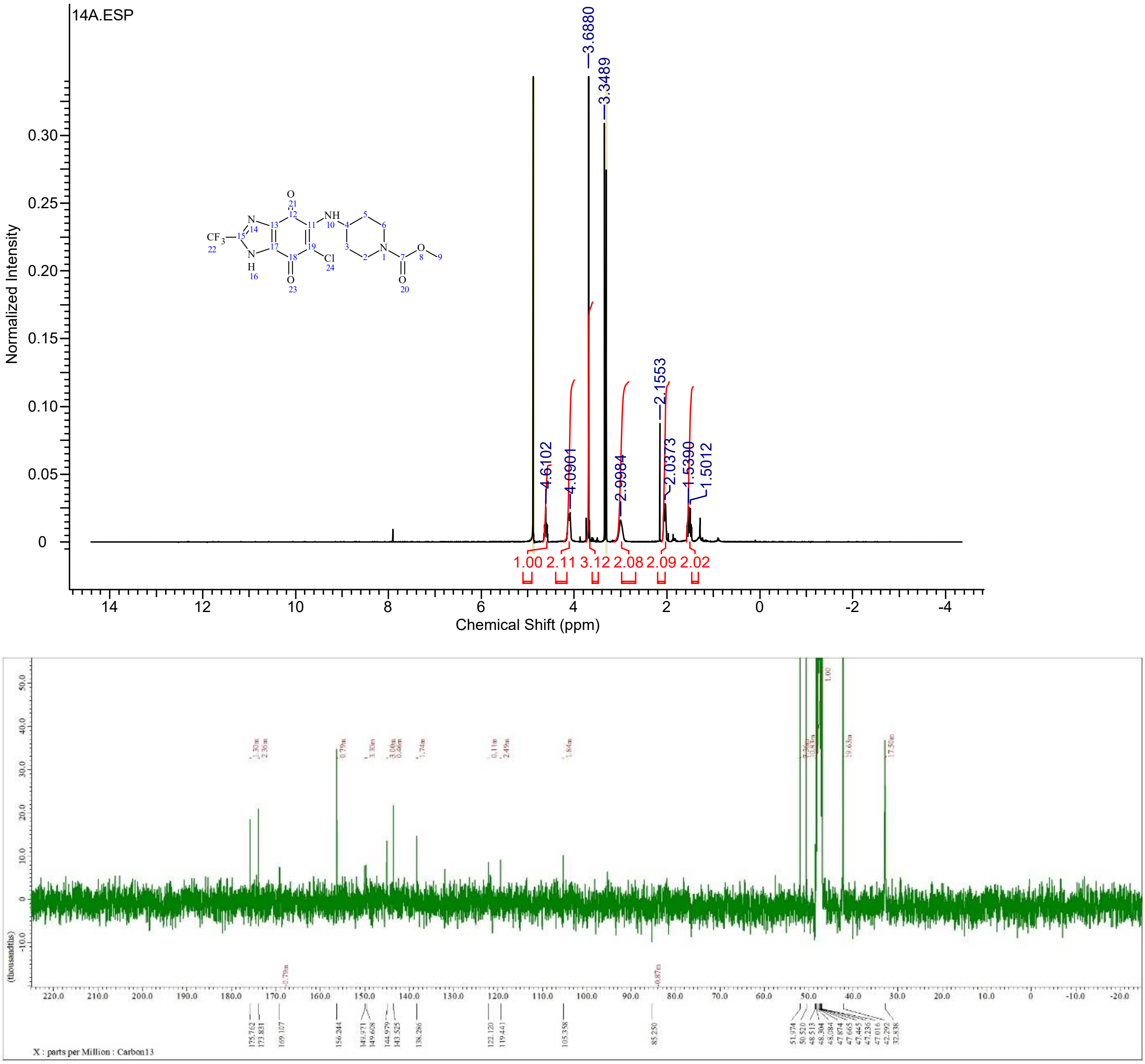

Figure S25. 1D 1H and 13C NMR spectrum of compound 14a

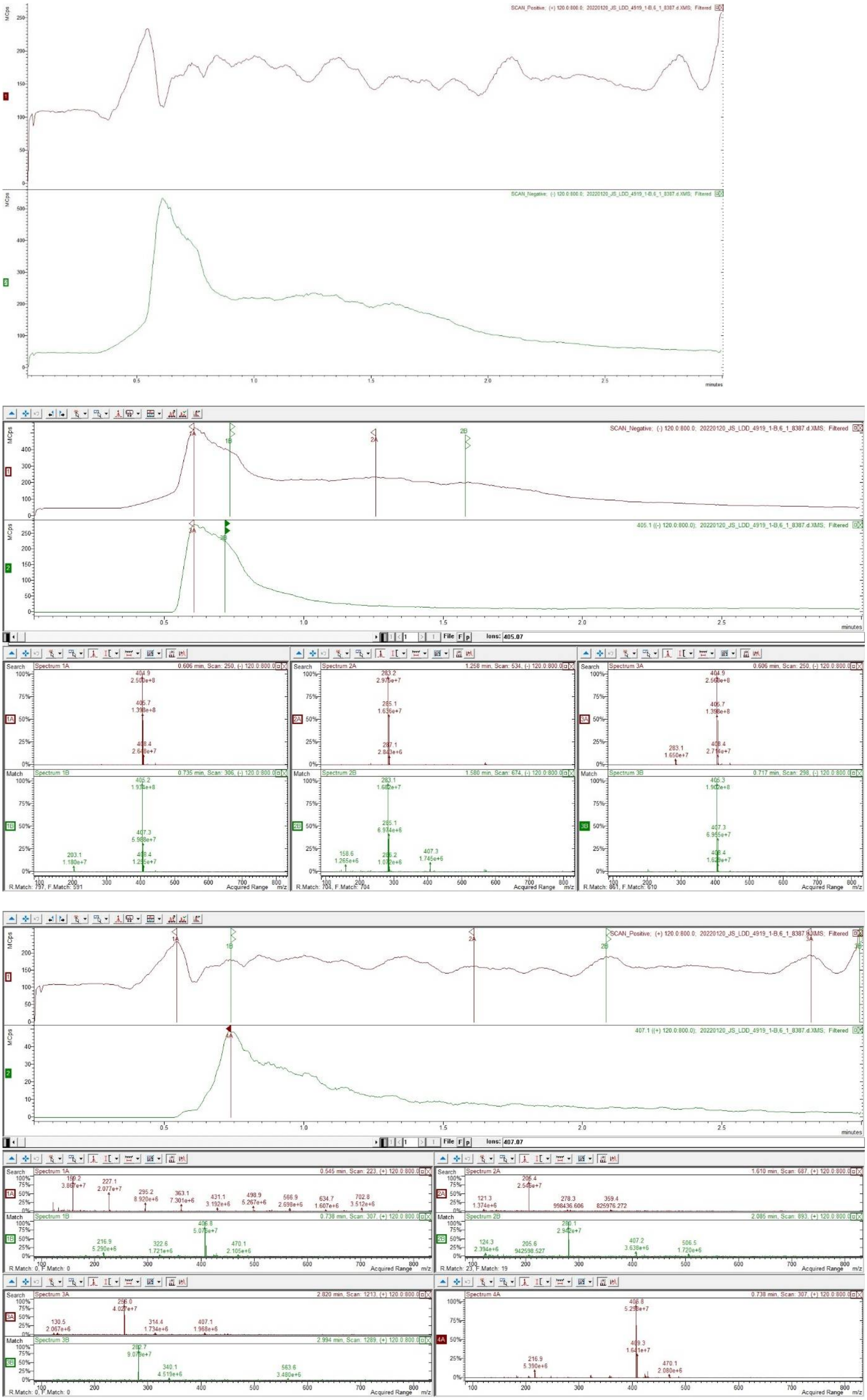

Figure S26. LC/MS spectrum of compound 14a

6-chloro-5-((1-propionylpiperidin-4-yl)amino)-2-(trifluoromethyl)-1H-benzo[d]imidazole-4,7-dione (14b) Following the general procedure for the synthesis of **14a-k**, the substitution reaction of **9b** with **13b** afforded **14b**. red purple powder. **14b** fully dissolved in 600 μL of the methanol-d4 and a drop of acetone. Yield 30%; ^{1}H NMR (400 MHz, METHANOL-d4) δ ppm 1.06 - 1.16 (m, 3 H) 1.42 - 1.56 (m, 1 H) 1.56 - 1.64 (m, 1 H) 2.00 - 2.15 (m, 2 H) 2.44 (q, J=7.48 Hz, 2 H) 2.82 (t, J=14.20 Hz, 1 H) 3.22 (t, J=11.68 Hz, 1 H) 3.98 (d, J=15.11 Hz, 1 H) 4.51 (d, J=14.66 Hz, 1 H) 4.67 (s, 1 H); ^{13}C NMR (100 MHz, METHANOL-d4) δ 175.44, 173.79, 173.41, 149.41, 144.47, 143.58, 138.12, 121.97, 119.29, 50.62, 43.92, 40.20, 25.95, 8.64, other peaks are impurities and acetone; LC/MS (ESI, m/z) 403.3 [M - H]$^{-}$ 405.4 [M + H]$^{+}$.

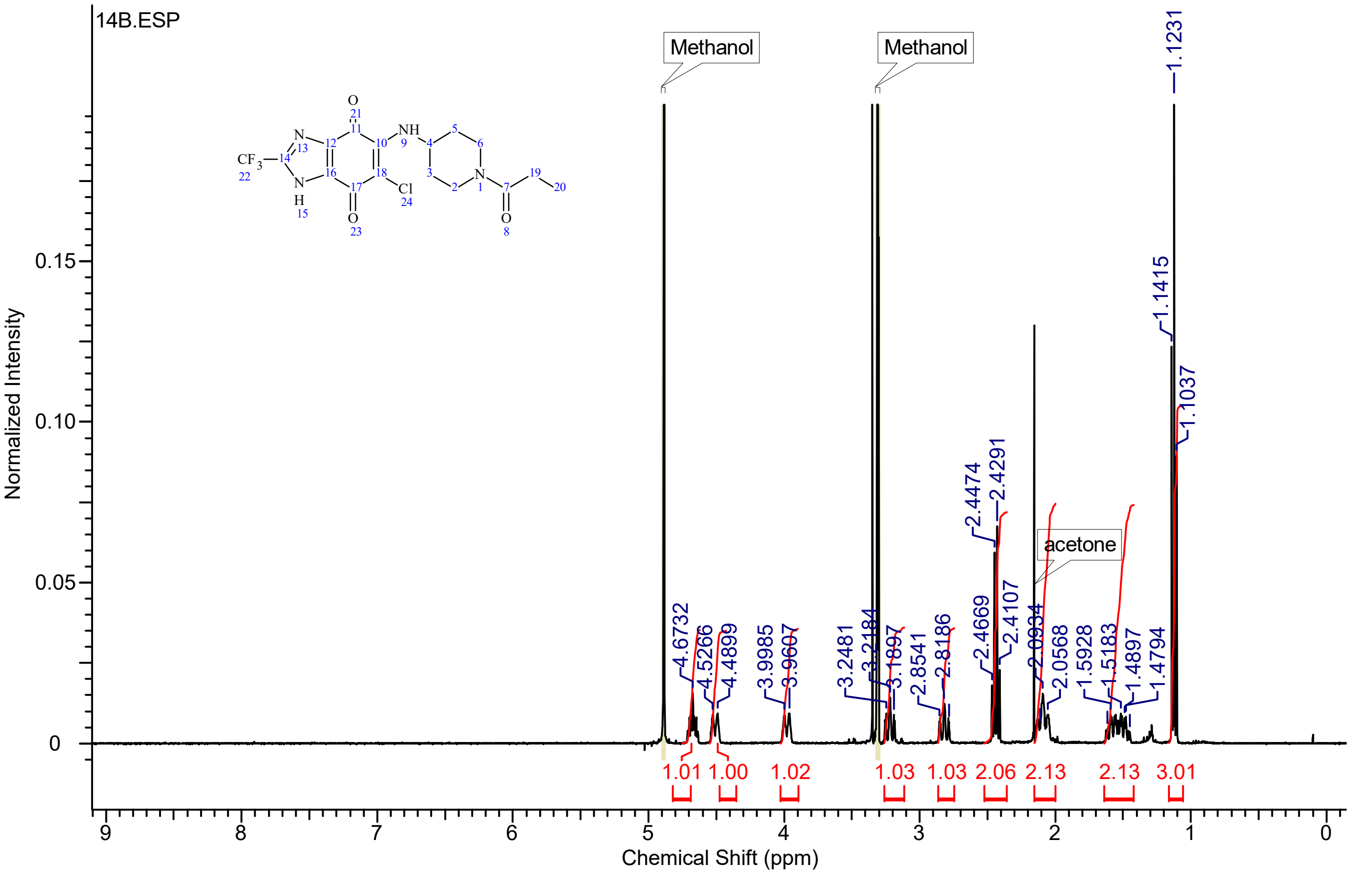

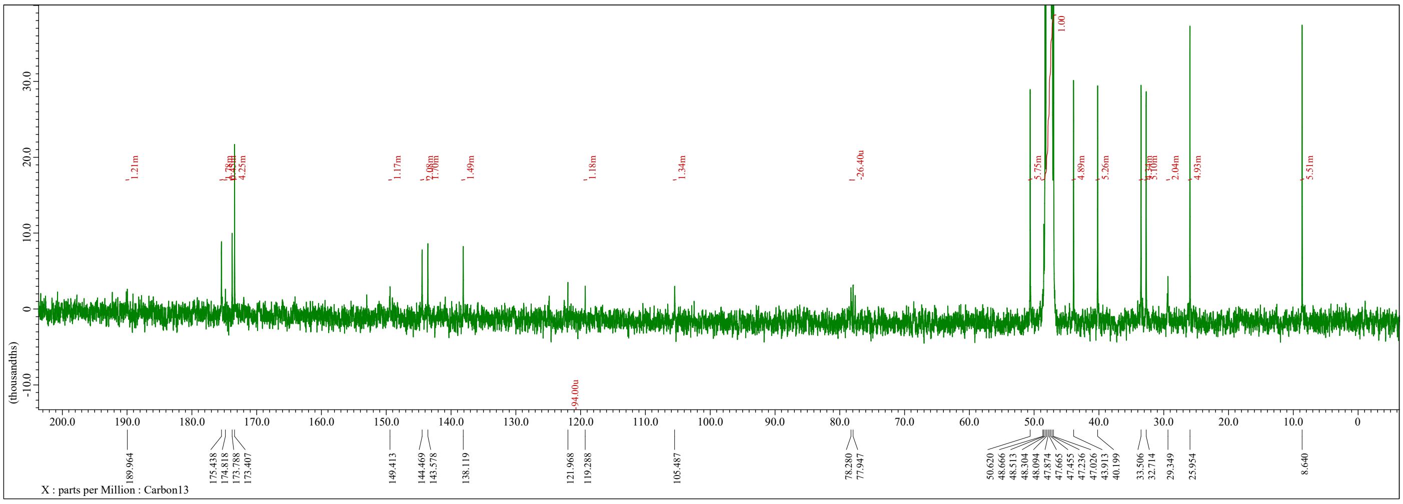

Figure S27. 1D 1H and 13C NMR spectrum of compound 14b

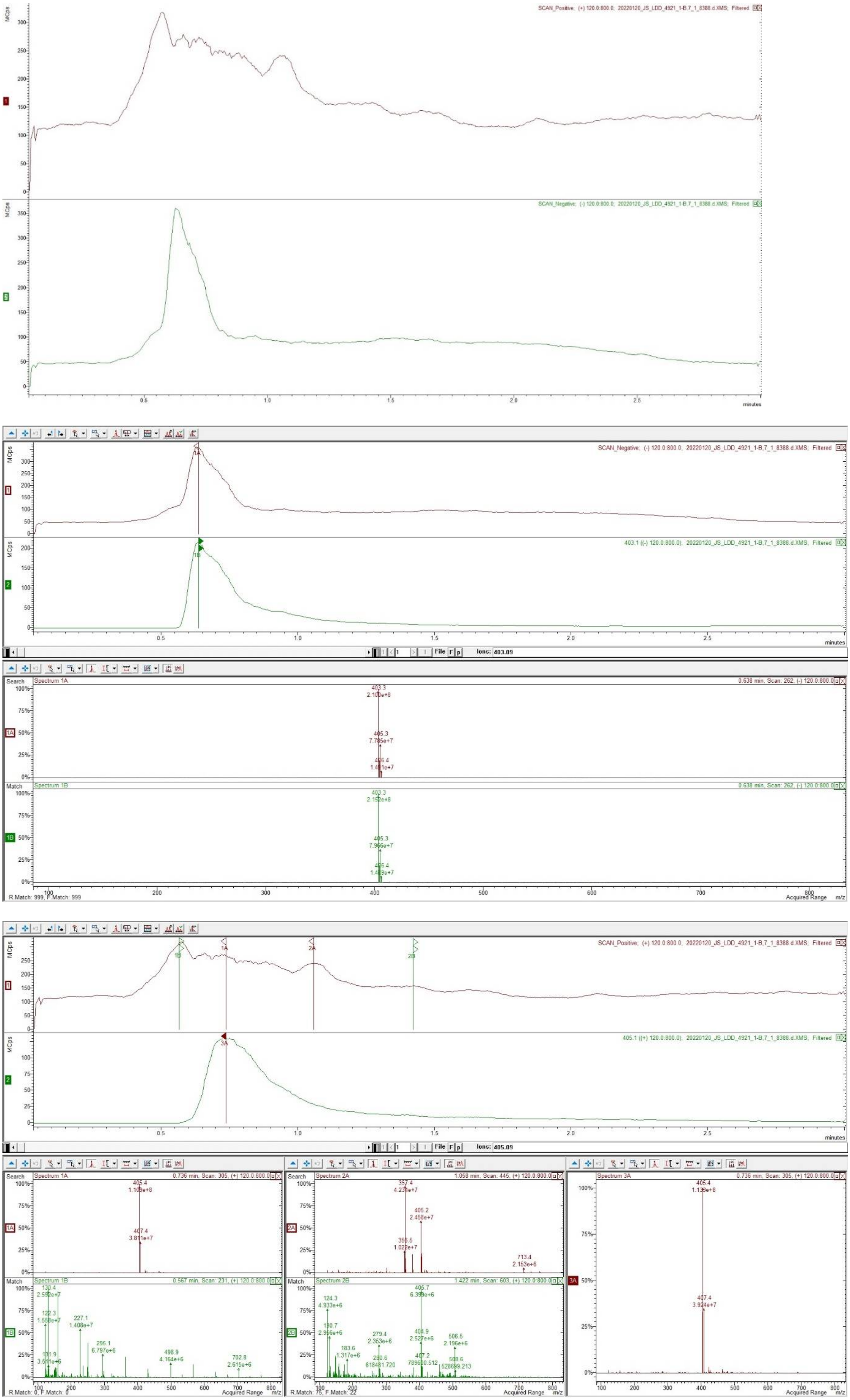

Figure S28. LC/MS spectrum of compound 14b

5-((1-benzoylpiperidin-4-yl)amino)-6-chloro-2-(trifluoromethyl)-1H-benzo[d]imidazole-4,7-dione (14c) Following the general procedure for the synthesis of **14a-k**, the substitution reaction of **9b** with **13c** afforded **14c**. purple powder. Yield 43%; **14c** fully dissolved in 600 µL of the methanol-d4 and a drop of acetone. ^{1}H NMR (400 MHz, METHANOL-d4) δ ppm 1.66 (d, J=13.74 Hz, 2 H) 2.02 (br. s., 1 H) 2.20 (br. s., 1 H) 3.05 (br. s., 1 H) 3.26 (br. s., 1 H) 3.76 (br. s., 1 H) 4.63 (br. s., 1 H) 4.75 (s, 1 H) 7.42 - 7.50 (m, 5 H); ^{13}C NMR (100 MHz, METHANOL-d4) δ 173.56, 171.21, 146.20, 143.85, 141.35, 136.89, 135.64, 129.76, 129.37, 128.41, 126.51, 50.88, 46.23, 40.68, 2 carbon peaks in 173.56 ppm overlapped and peaks in 33 and 32 ppm are acetone; LC/MS (ESI, m/z) 451.5 $[M - H]^-$ 453.1 $[M + H]^+$.

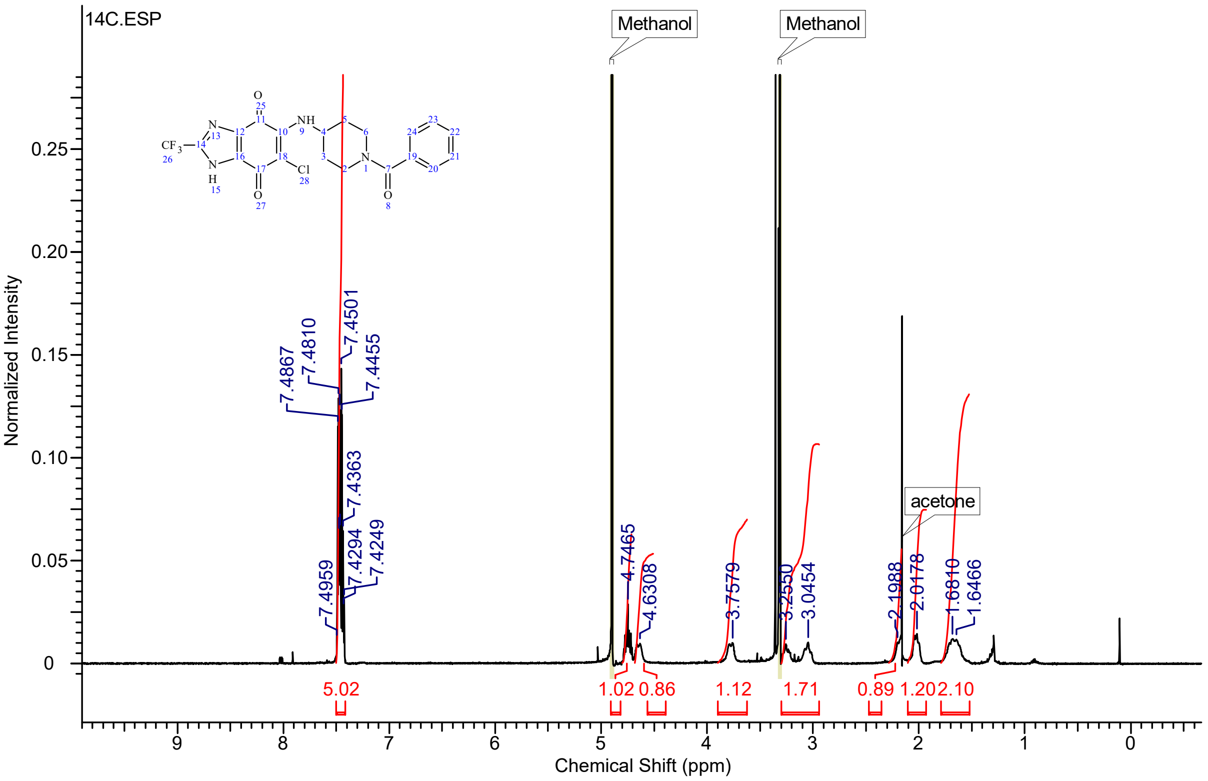

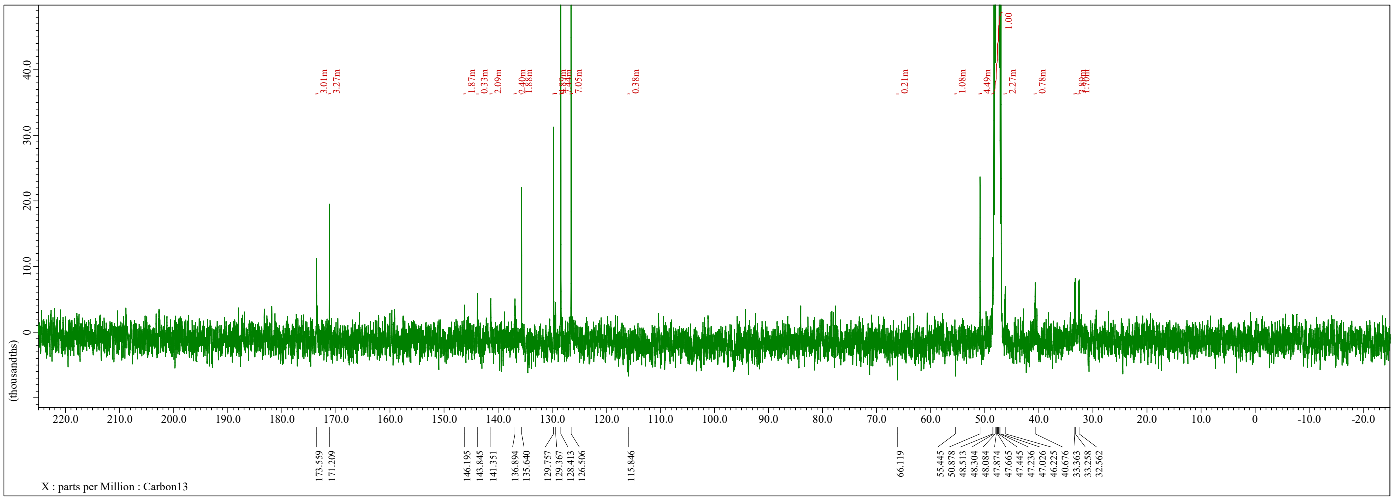

Figure S29. 1D 1H and 13C NMR spectrum of compound 14c

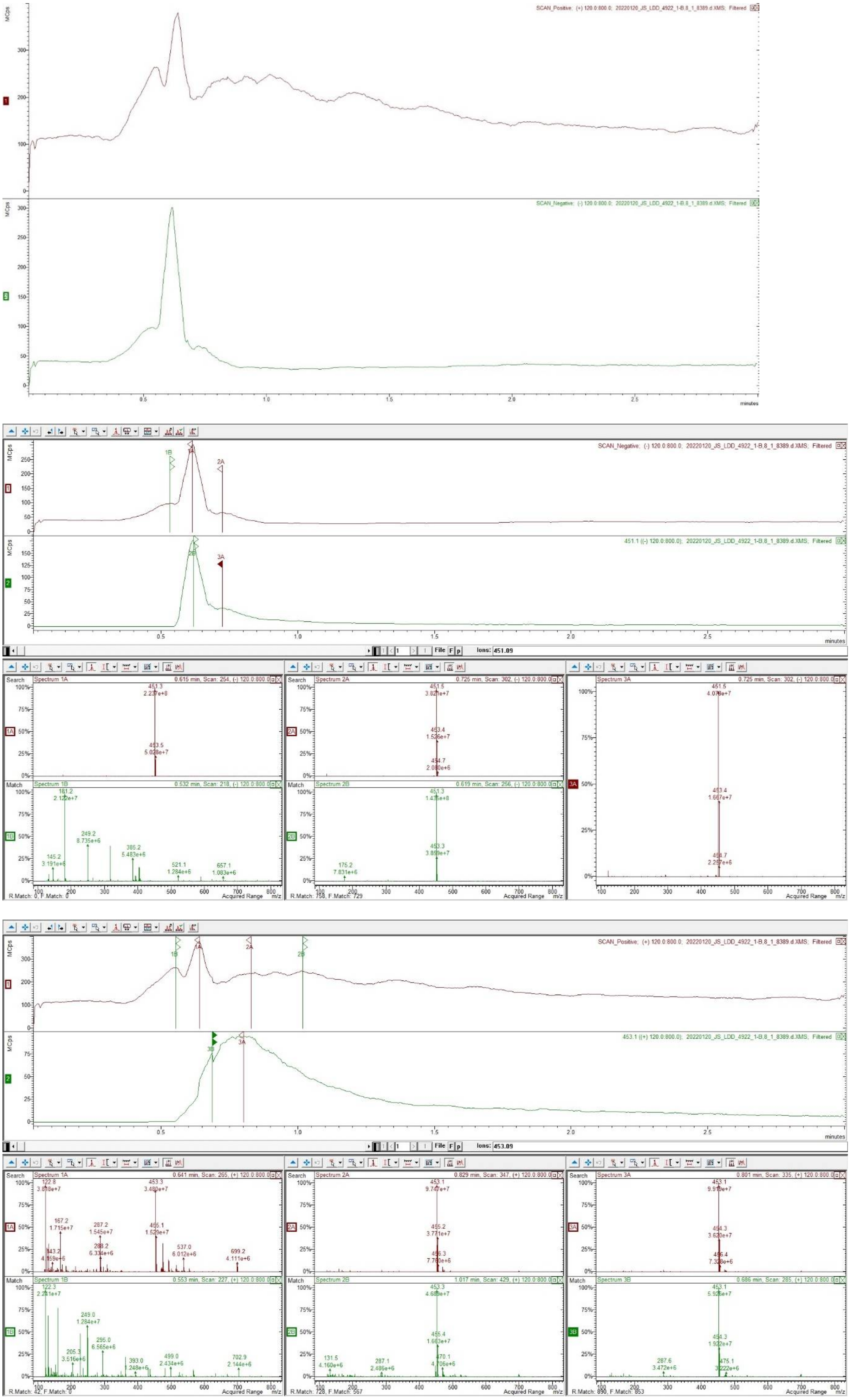

Figure S30. LC/MS spectrum of compound 14c

6-chloro-5-((1-(4-fluorobenzoyl)piperidin-4-yl)amino)-2-(trifluoromethyl)-1H-benzo[d]imidazole-4,7-dione (14d)

Following the general procedure for the synthesis of **14a-k**, the substitution reaction of **9b** with **13d** afforded **14d**. red purple powder. **14d** fully dissolved in 600 μL of the acetone-d6 and a drop of methanol. Yield 67%; m.p. 205-206 ℃; ^{1}H NMR (400 MHz, ACETONE-d6) δ ppm 1.65 - 1.85 (m, 2 H) 2.11 (br. s., 2 H) 2.98 - 3.28 (m, 2 H) 3.80 (br. s., 1 H) 4.56 (br. s., 1 H) 4.76 (br. s., 1 H) 6.47 (br. s., 1 H) 7.14 - 7.24 (m, 2 H) 7.45 - 7.54 (m, 2 H); ^{13}C NMR (100 MHz, DMSO-d6) δ 174.06, 173.14, 168.60, 144.11, 137.82, 137.76, 133.13, 133.10, 129.79, 129.70, 116.05, 115.83, 105.68, 51.15, 49.12, 46.49, peak in 33 ppm is acetone; LC/MS (ESI, m/z) 469.4 [M - H]$^-$ 471.1 [M + H]$^+$.

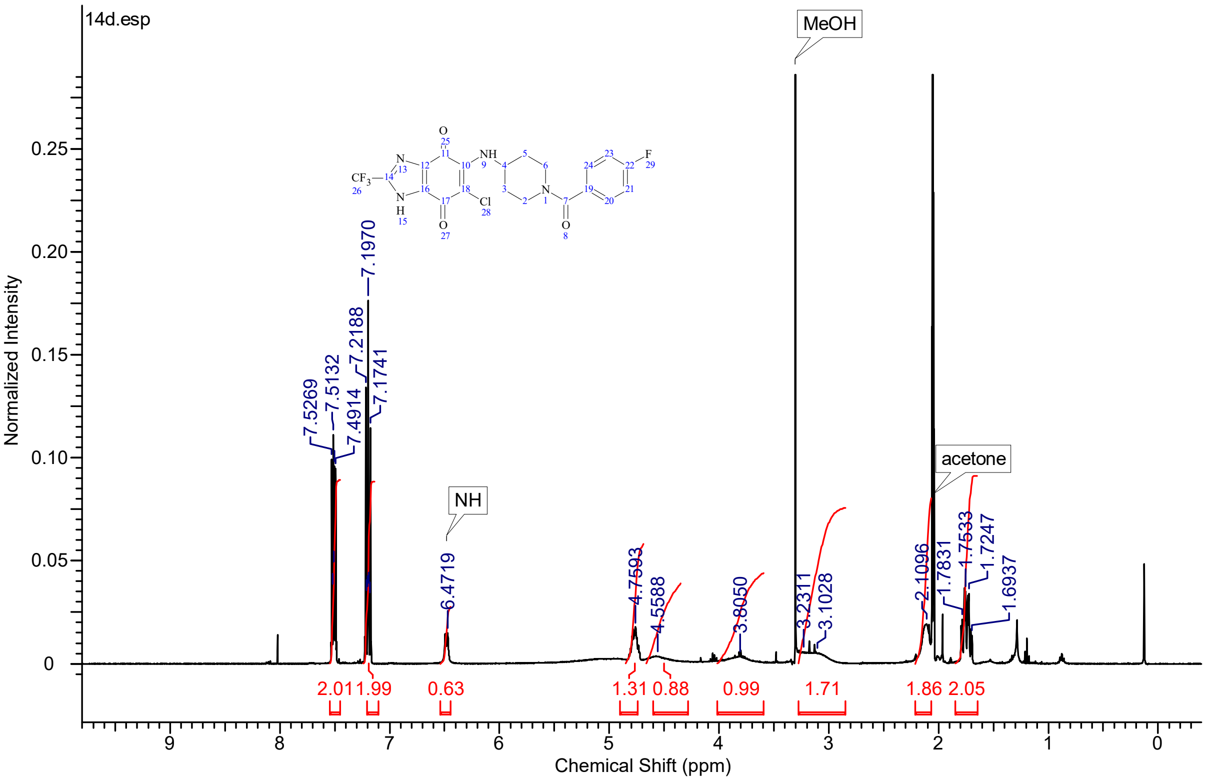

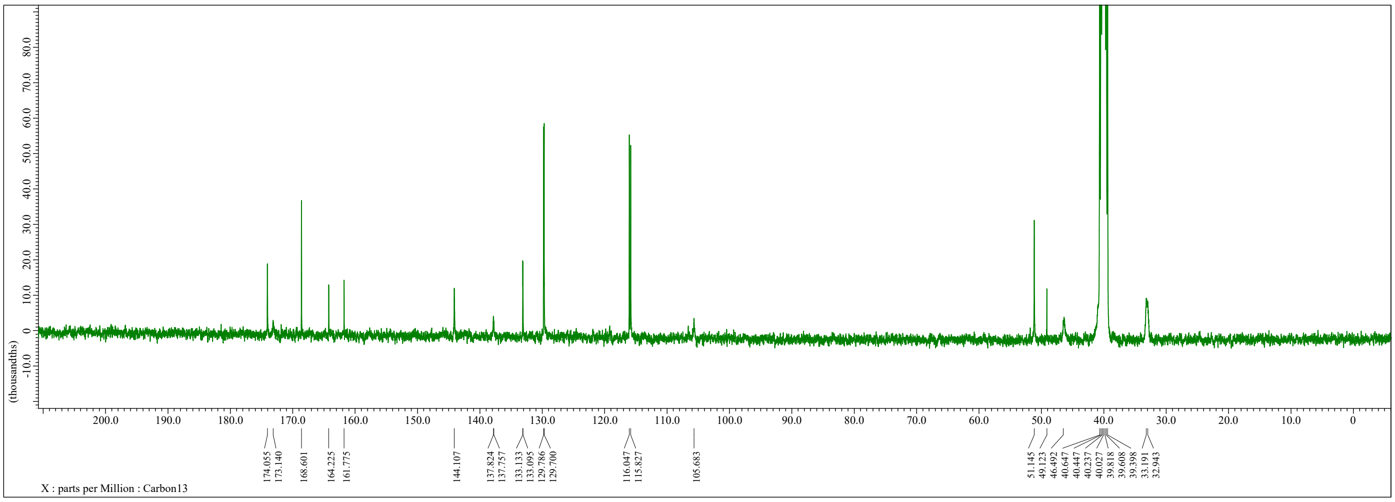

Figure S31. 1D 1H and 13C NMR spectrum of compound 14d

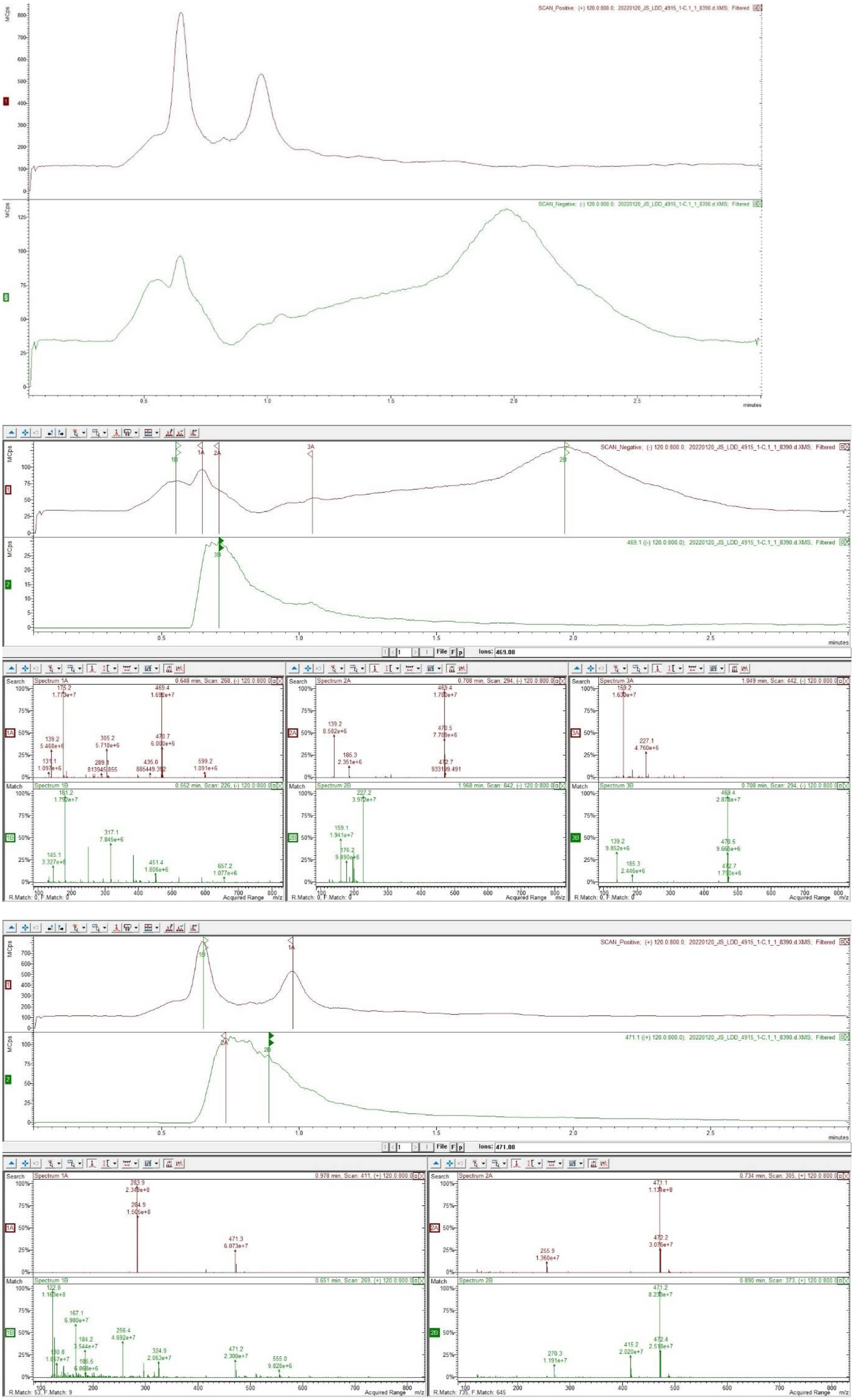

Figure S32. LC/MS spectrum of compound 14d

6-chloro-5-((1-(4-chlorobenzoyl)piperidin-4-yl)amino)-2-(trifluoromethyl)-1H-benzo[d]imidazole-4,7-dione (14e)
Following the general procedure for the synthesis of **14a-k**, the substitution reaction of **9b** with **13e** afforded **14e**. purple powder. Yield 77%; m.p. 249-251 ℃; ^{1}H NMR (400 MHz, METHANOL-d4) δ ppm 1.65 (br. s., 2 H) 2.03 (br. s., 1 H) 2.16 (br. s., 1 H) 3.04 (br. s., 1 H) 3.27 (br. s., 1 H) 3.74 (br. s., 1 H) 4.61 (br. s., 1 H) 4.73 (s, 1 H) 7.42 - 7.45 (m, 2 H) 7.46 - 7.50 (m, 2 H); ^{13}C NMR (100 MHz, METHANOL-d4) δ 173.72, 173.46, 170.02, 143.95, 141.67, 136.97, 135.70, 134.19, 128.61, 128.37, 50.84, 46.27, 40.77, 2 carbon peaks in 141.67 ppm are overlapped; LC/MS (ESI, m/z) 485.3 $[M - H]^{-}$ 487.1 $[M + H]^{+}$.

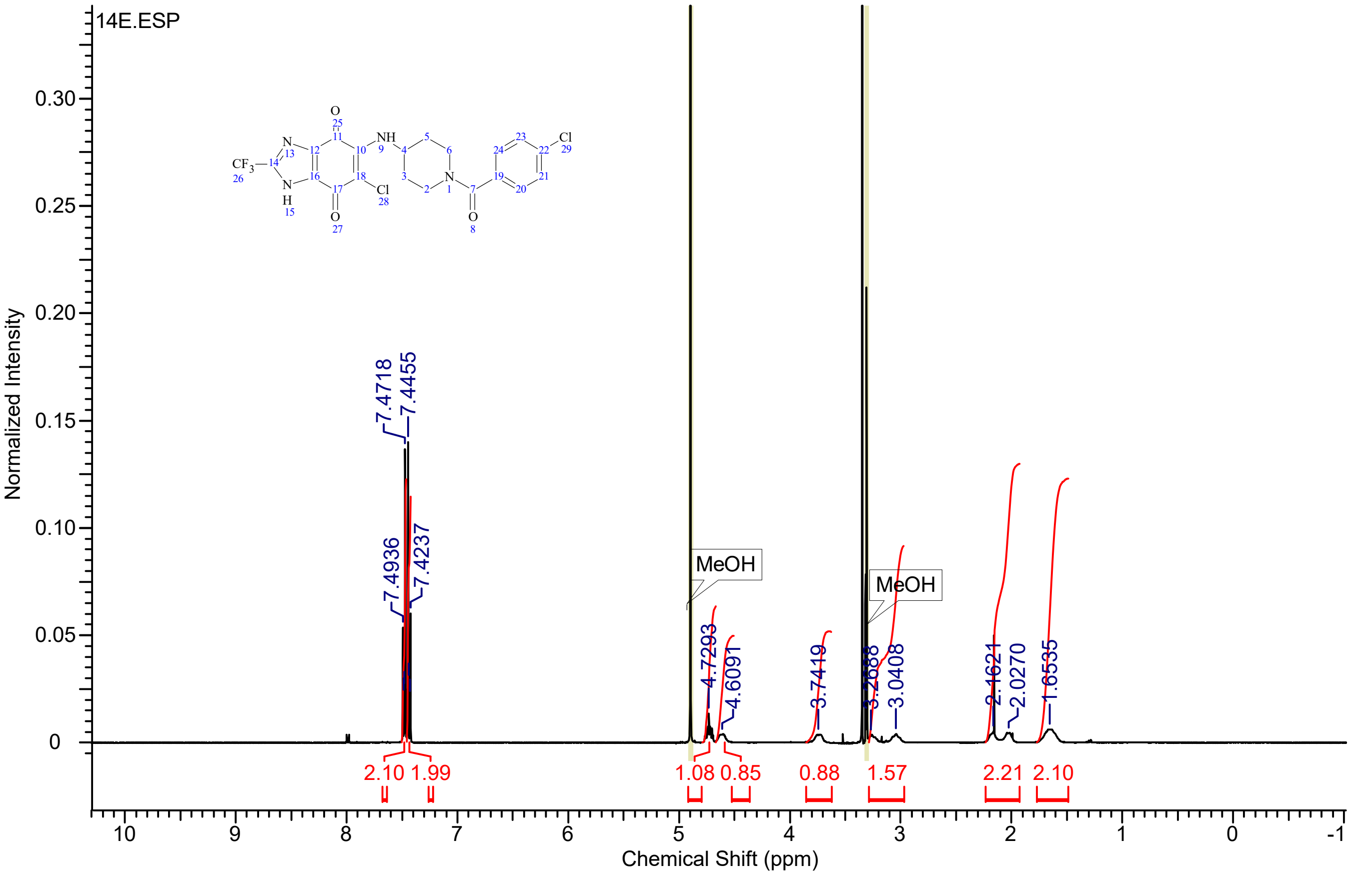

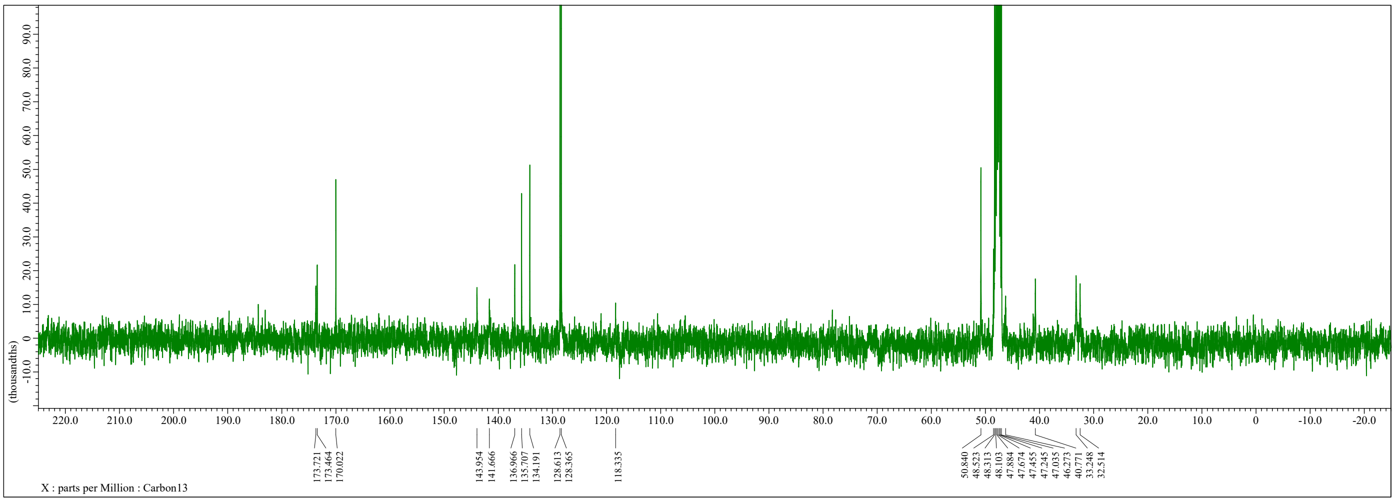

Figure S33. 1D 1H and 13C NMR spectrum of compound 14e

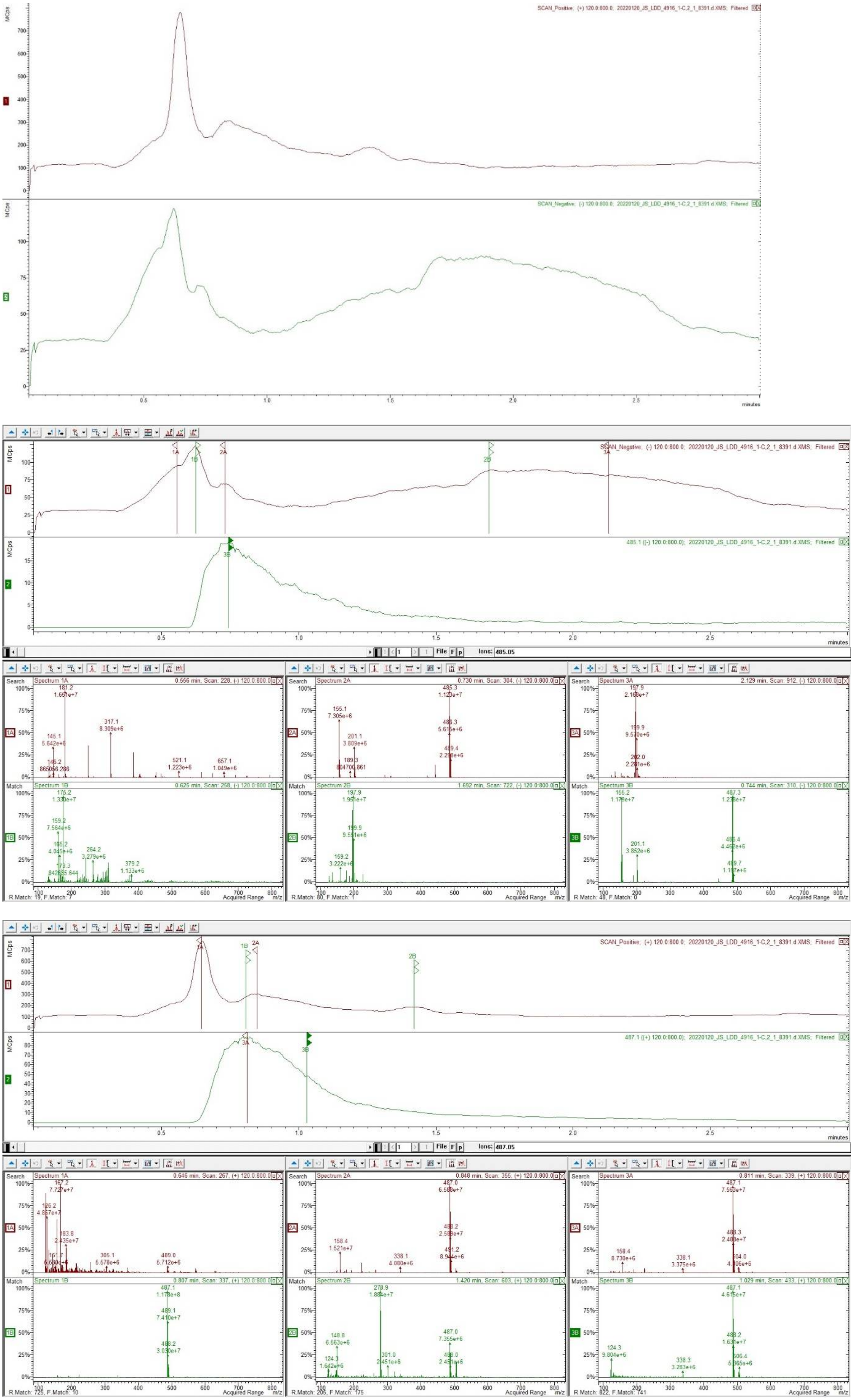

Figure S34. LC/MS spectrum of compound 14e

5-((1-(4-bromobenzoyl)piperidin-4-yl)amino)-6-chloro-2-(trifluoromethyl)-1H-benzo[d]imidazole-4,7-dione (14f)

Following the general procedure for the synthesis of **14a-k**, the substitution reaction of **9b** with **13f** afforded **14f**. purple powder. **14f** fully dissolved in 600 μL of the methanol-d4 and a drop of acetone. Yield 68%; ^{1}H NMR (400 MHz, METHANOL-d4) δ ppm 1.65 (br. s., 2 H) 2.04 (br. s., 1 H) 2.20 (br. s., 1 H) 3.06 (br. s., 1 H) 3.27 (br. s., 1 H) 3.73 (br. s., 1 H) 4.61 (br. s., 1 H) 4.76 (s, 1 H) 7.38 (d, J=8.70 Hz, 2 H) 7.65 (d, J=8.24 Hz, 2 H); ^{13}C NMR (100 MHz, METHANOL-d4) δ 173.46, 173.36, 170.07, 143.99, 141.08, 138.59, 136.80, 134.63, 131.63, 128.51, 123.81, 120.86, 118.17, 115.484, 50.88, 46.25, 40.72, other peaks are impurities; LC/MS (ESI, m/z) 529.2 $[M - H]^-$ 532.9 $[M + H]^+$.

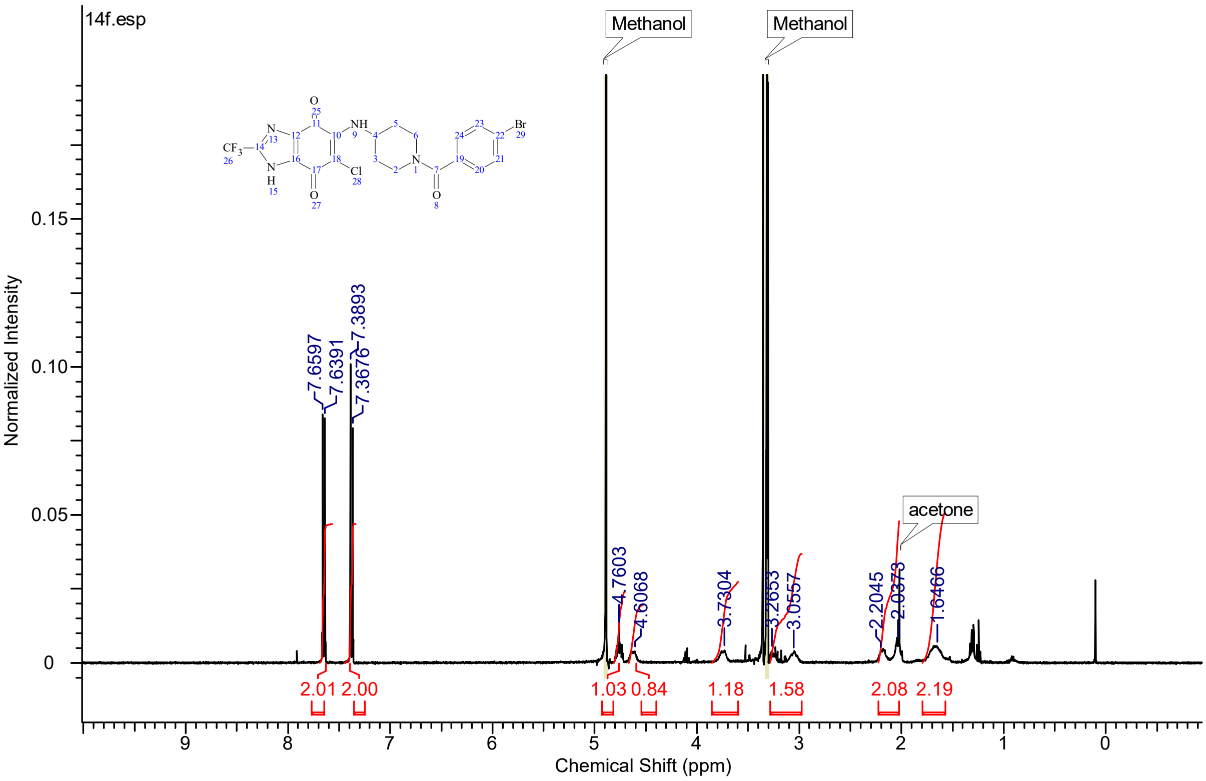

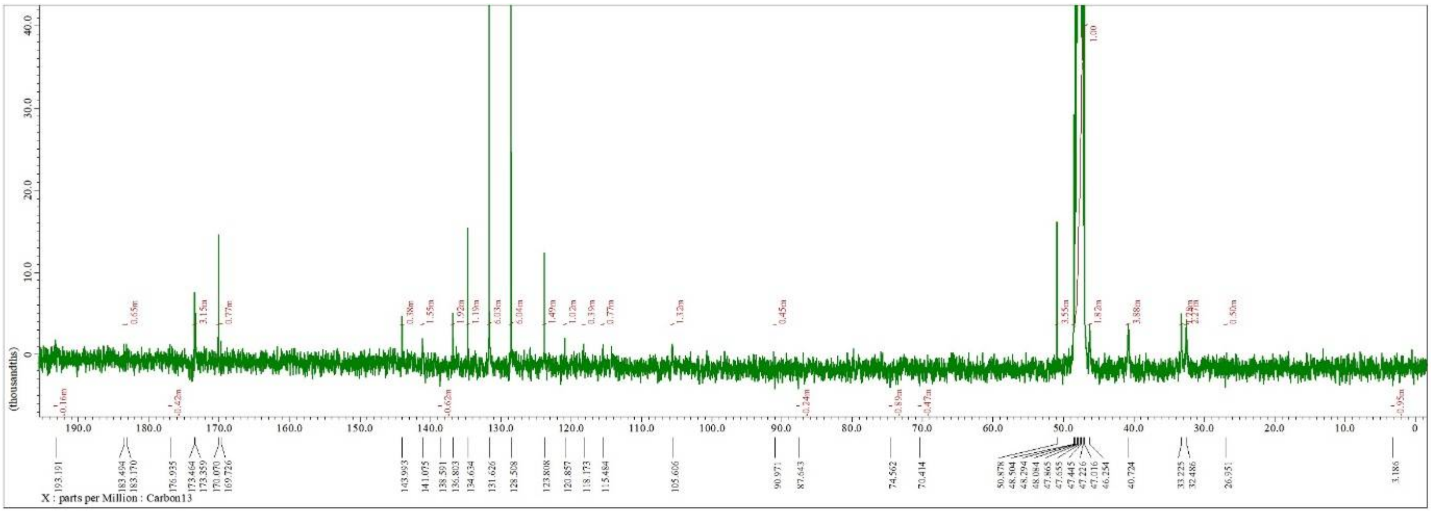

Figure S35. 1D 1H and 13C NMR spectrum of compound 14f

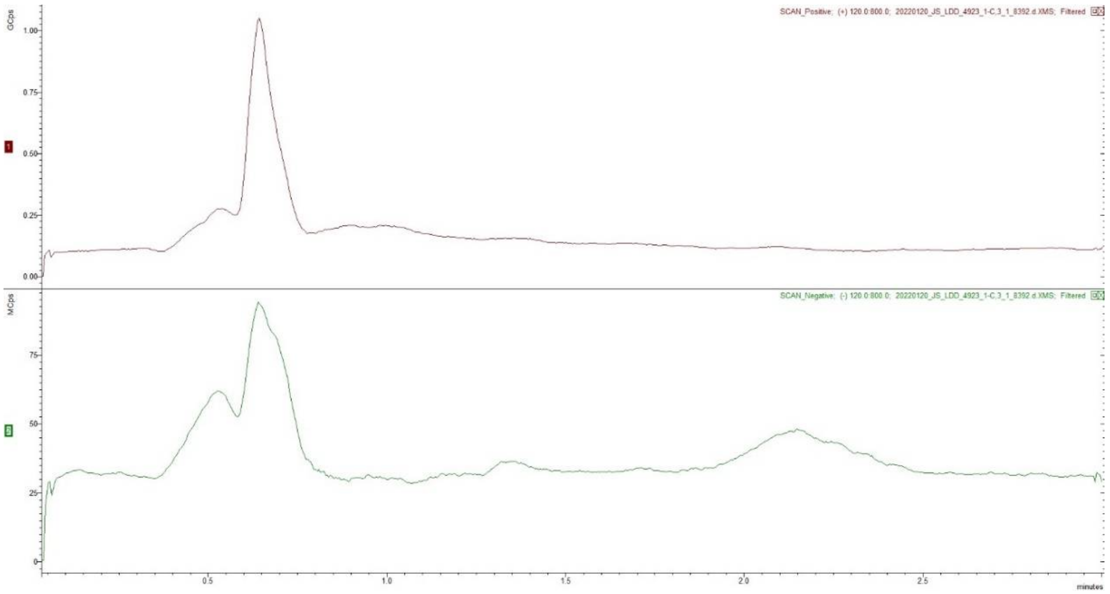

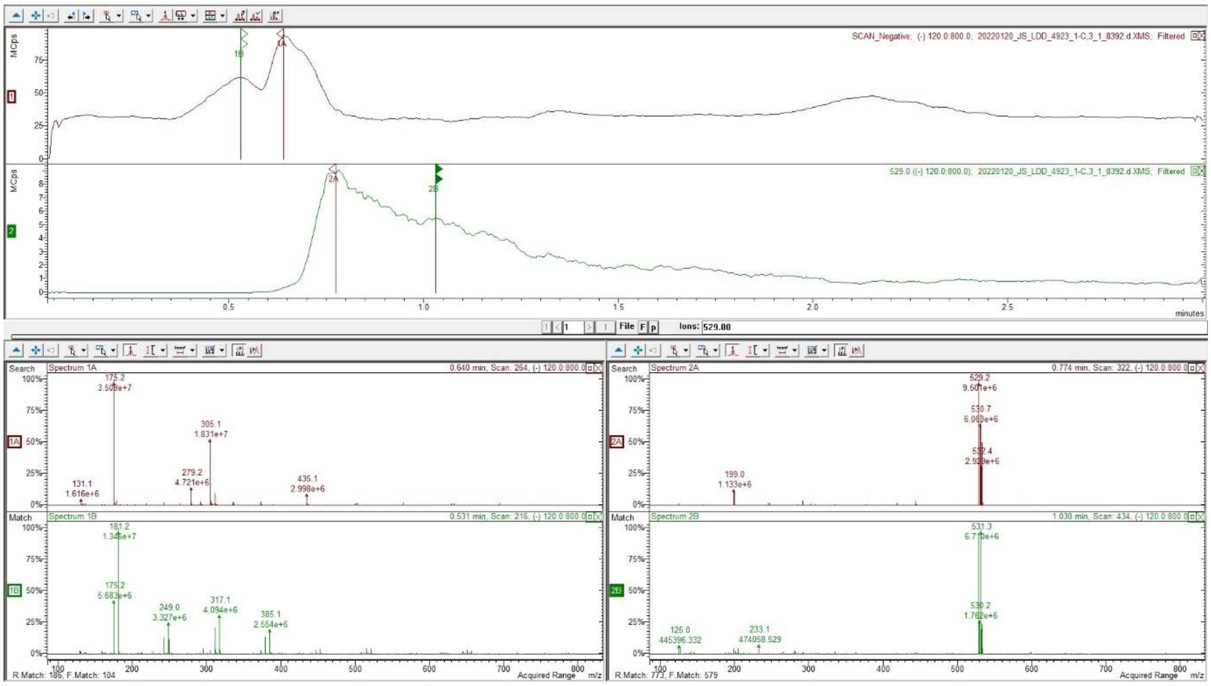

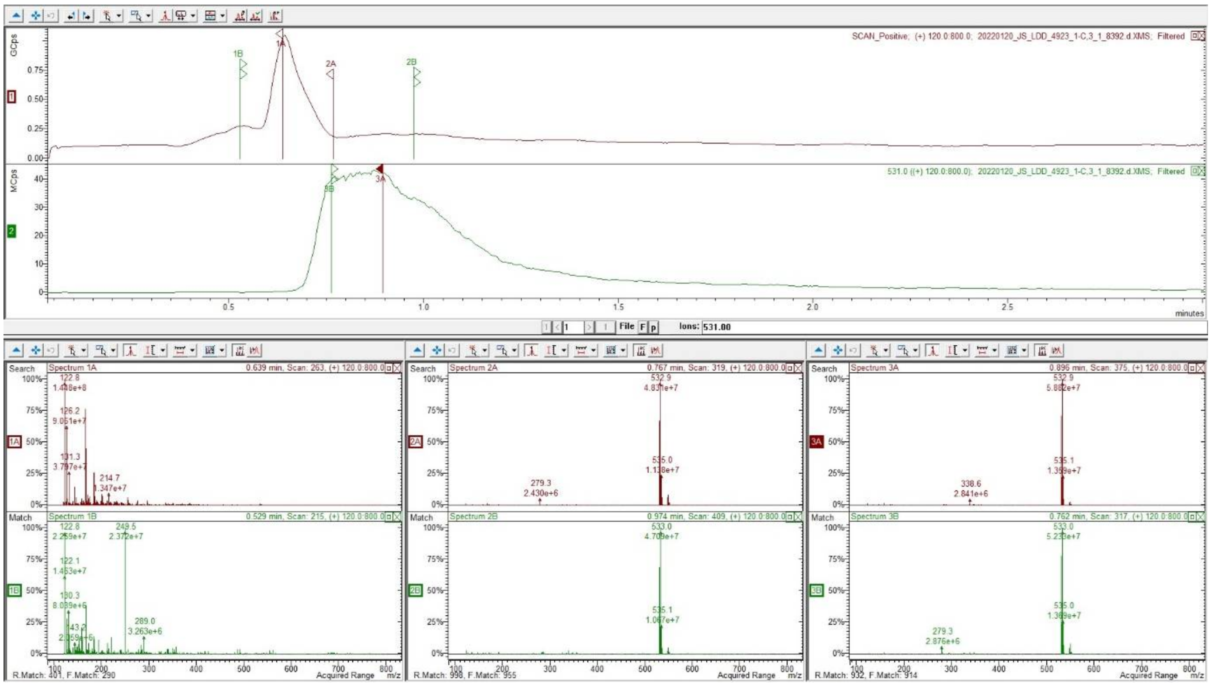

Figure S36. LC/MS spectrum of compound 14f

6-chloro-5-((1-(3,4-dichlorobenzoyl)piperidin-4-yl)amino)-2-(trifluoromethyl)-1H-benzo[d]imidazole-4,7-dione (14g)

Following the general procedure for the synthesis of **14a-k**, the substitution reaction of **9b** with **13g** afforded **14g**. purple powder. Yield 66%; ^{1}H NMR (400 MHz, METHANOL-d4) δ ppm 1.68 (br. s., 2 H) 2.03 (br. s., 1 H) 2.18 (br. s., 1 H) 3.05 (br. s., 1 H) 3.28 (br. s., 1 H) 3.72 (br. s., 1 H) 4.60 (br. s., 1 H) 4.76 (s, 1 H) 7.38 (dd, J=8.24, 1.83 Hz, 1 H) 7.58 - 7.68 (m, 2 H); ^{13}C NMR (100 MHz, METHANOL-d4) δ 174.24, 173.44, 168.49, 144.06, 142.34, 137.26, 135.90, 133.71, 132.55, 130.69, 128.84, 126.41, 123.50, 122.12, 118.55, 50.77, 46.21, 40.87, 40.72, other peaks are impurities; LC/MS (ESI, m/z) 519.2 [M - H]$^{-}$ 521.0 [M + H]$^{+}$.

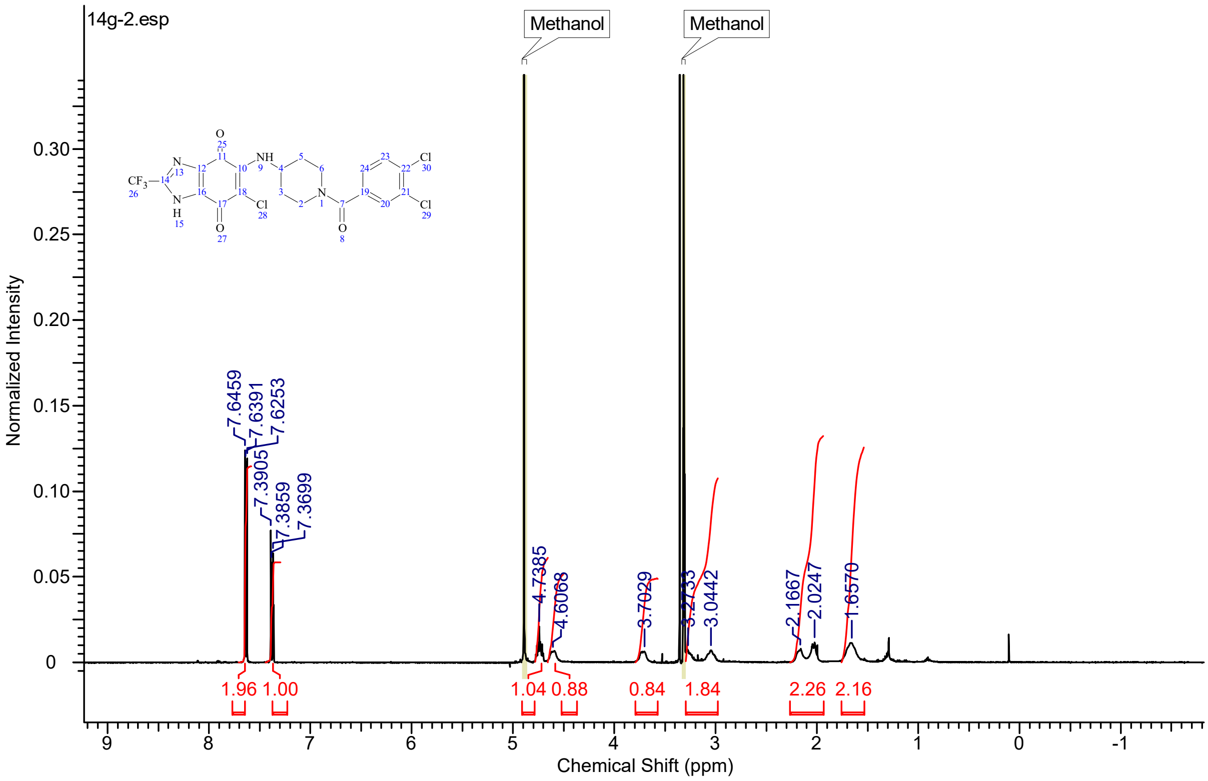

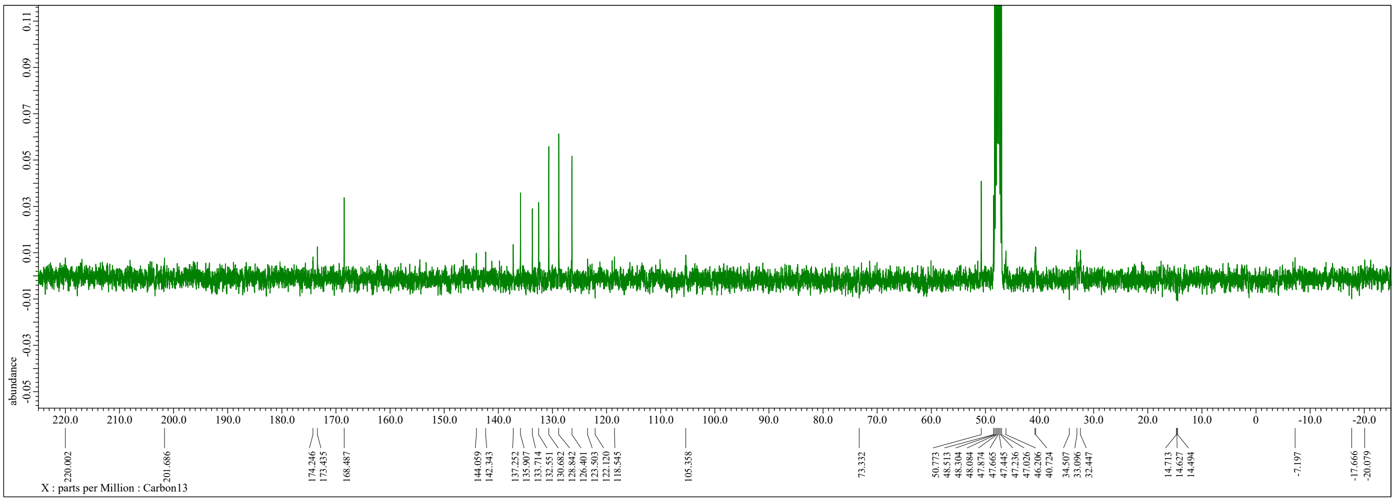

Figure S37. 1D 1H and 13C NMR spectrum of compound 14g

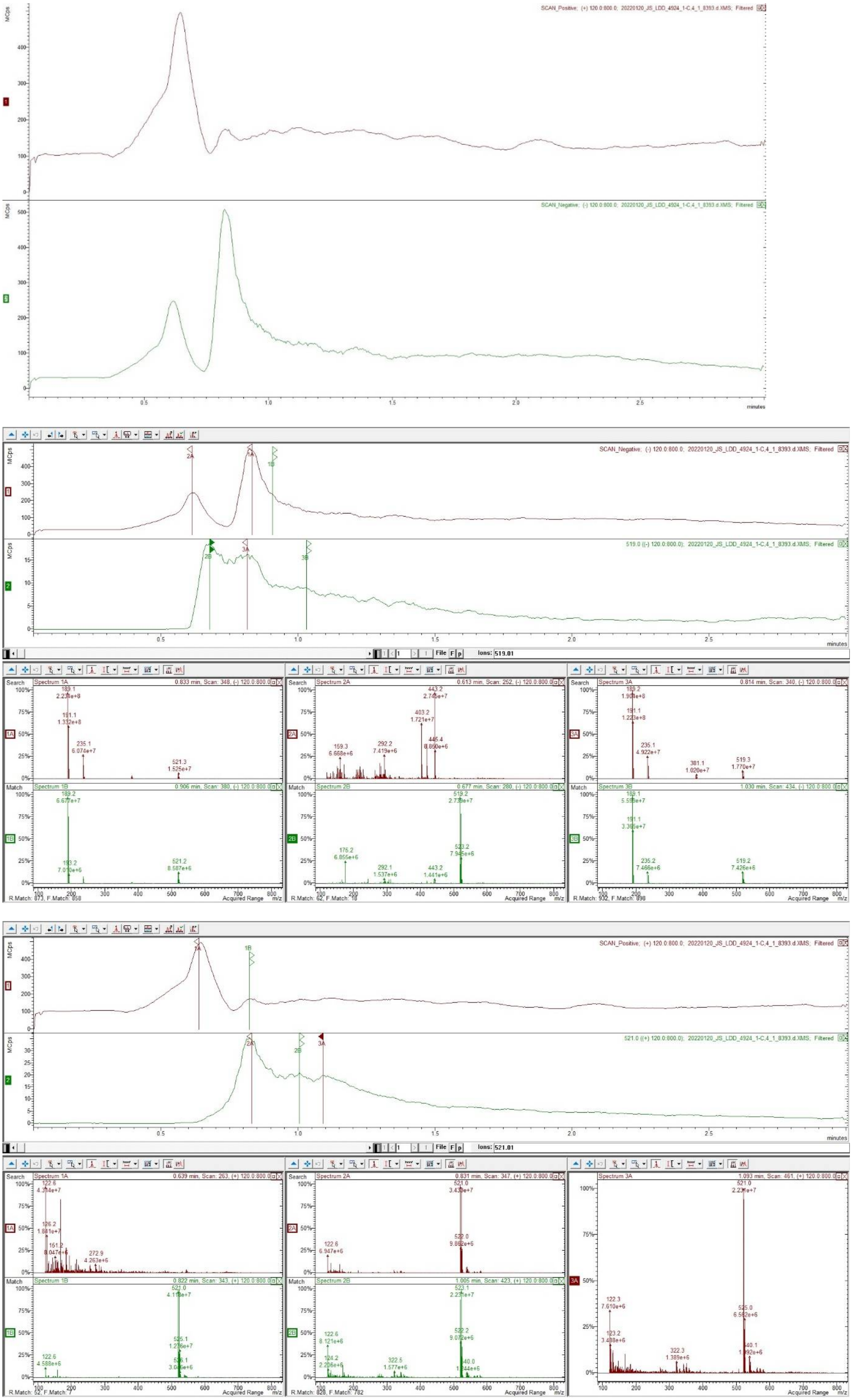

Figure S38. LC/MS spectrum of compound 14g

6-chloro-5-((1-(3,4-difluorobenzoyl)piperidin-4-yl)amino)-2-(trifluoromethyl)-1H-benzo[d]imidazole-4,7-dione (14h)

Following the general procedure for the synthesis of **14a-k**, the substitution reaction of **9b** with **13h** afforded **14h**. red purple powder. Yield 67%; m.p. 204-205 ℃; ^{1}H NMR (400 MHz, METHANOL-d4) δ ppm 1.57 (br. s., 2 H) 1.99 (br. s., 1 H) 2.11 (br. s., 1 H) 3.00 (br. s., 1 H) 3.20 (br. s., 1 H) 3.69 (br. s., 1 H) 4.50 (br. s., 1 H) 4.61 (s, 1 H) 7.18 - 7.24 (m, 1 H) 7.26 - 7.41 (m, 2 H); ^{13}C NMR (100 MHz, METHANOL-d4) δ 173.33, 173.02, 168.73, 143.97, 140.64, 136.48, 132.83, 132.78, 123.74, 123.70, 120.67, 117.99, 117.63, 117.46, 116.47, 116.29, 50.91, 46.24, 40.87, peaks in 152~148 ppm are impurities; LC/MS (ESI, m/z) 487.4 [M - H]$^-$ 489.1 [M + H]$^+$.

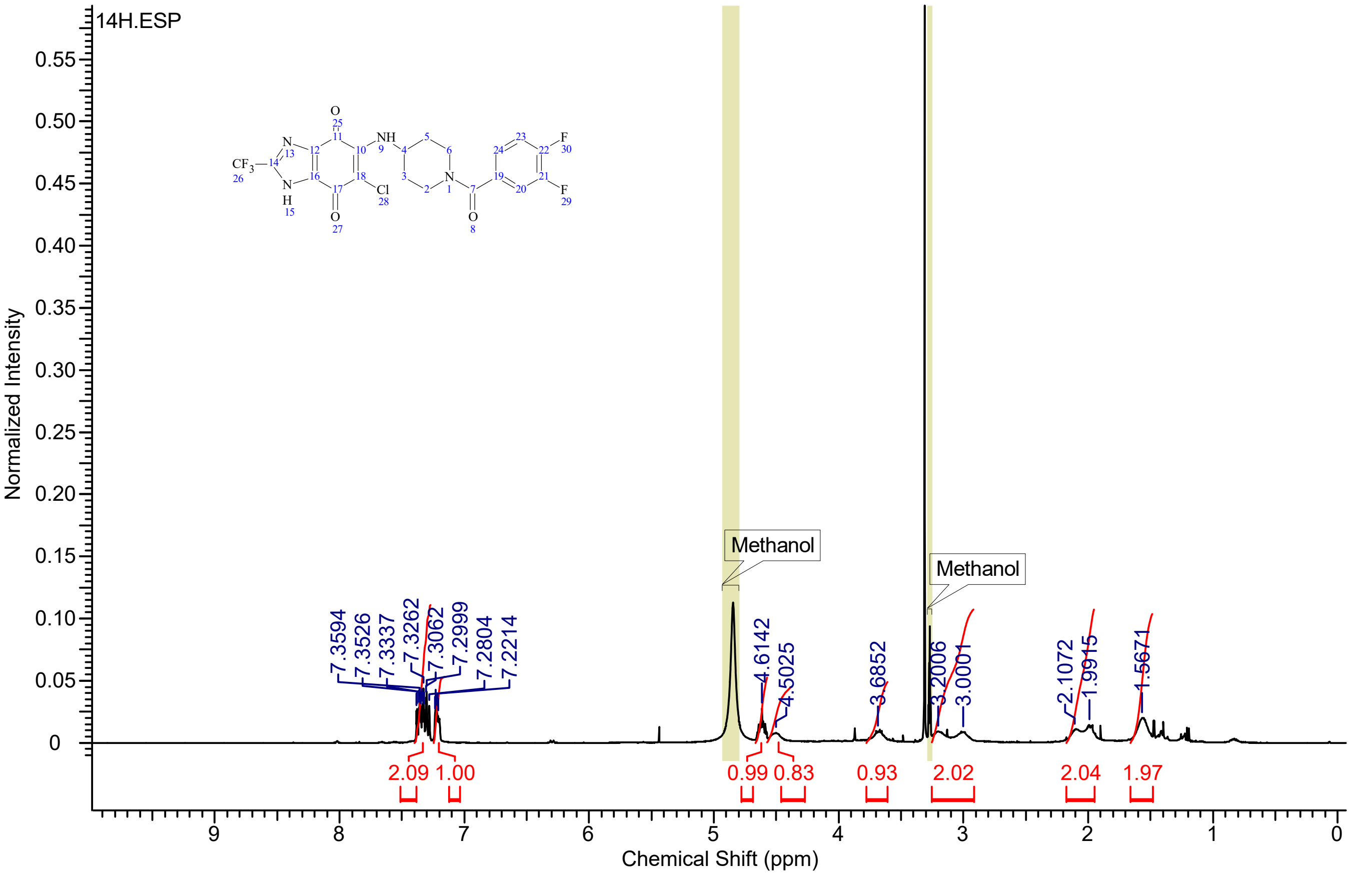

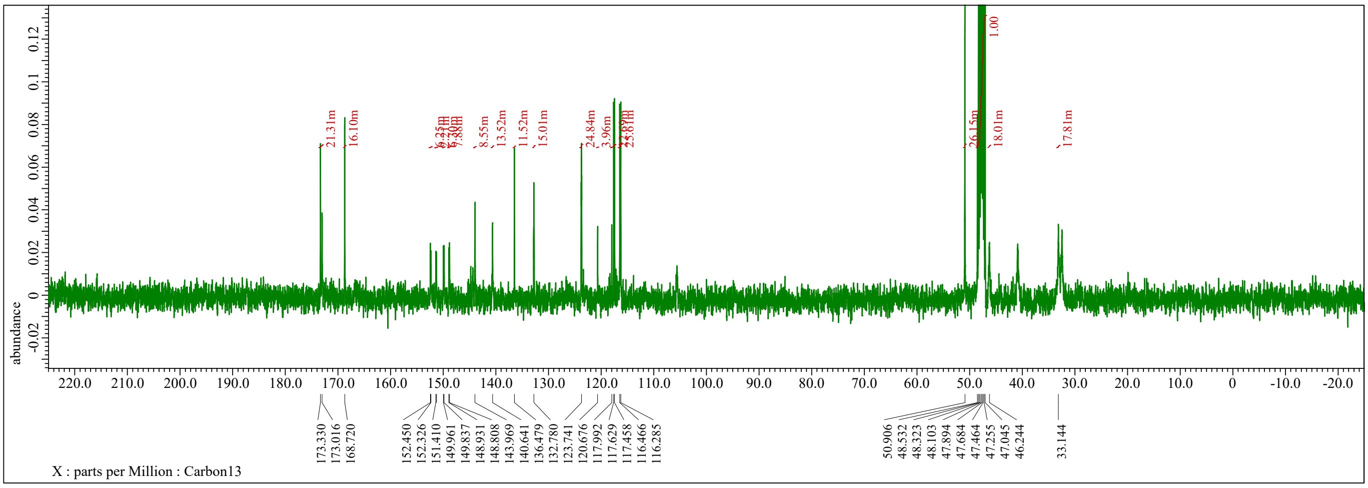

Figure S39. 1D 1H and 13C NMR spectrum of compound 14h

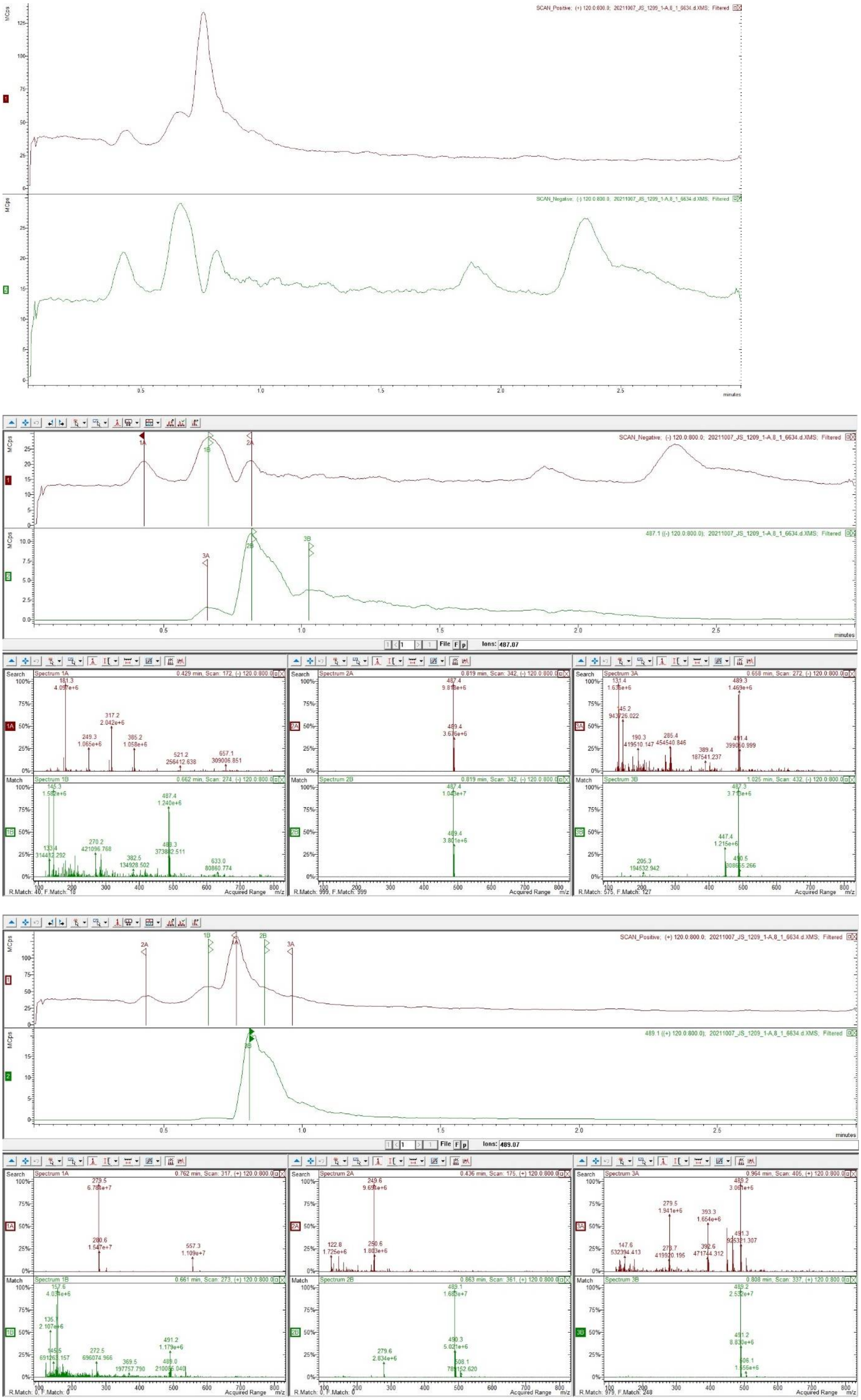

Figure S40. LC/MS spectrum of compound 14h

6-chloro-5-((1-(3-chloro-4-fluorobenzoyl)piperidin-4-yl)amino)-2-(trifluoromethyl)-1H-benzo[d]imidazole-4,7-dione (14i) Following the general procedure for the synthesis of **14a-k**, the substitution reaction of **9b** with **13i** afforded **14i**. red purple powder. **14i** fully dissolved in 600 μL of the acetone-d6 and a drop of methanol. Yield 75%; ^{1}H NMR (400 MHz, ACETONE-d6) δ ppm 1.68 - 1.84 (m, 2 H) 2.13 (br. s., 1 H) 3.4 (br. s., 2 H) 3.82 (br. s., 1 H) 4.51 (br. s., 1 H) 4.76 (br. s., 1 H) 7.35 - 7.41 (m, 1 H) 7.47 (ddd, J=8.47, 4.58, 2.06 Hz, 1 H) 7.58 - 7.62 (m, 1 H); ^{13}C NMR (100 MHz, METHANOL-d4) δ 173.49, 168.67, 136.98, 133.14, 129.42, 127.38, 127.31, 127.10, 121.10, 121.01, 120.92, 116.84, 116.73, 116.62, 50.79, 46.33, 41.43, 2 carbon peaks in 173.49 ppm overlapped and other peaks are impurities; LC/MS (ESI, m/z) 505.1 $[M - H]^-$ 503.3 $[M + H]^+$.

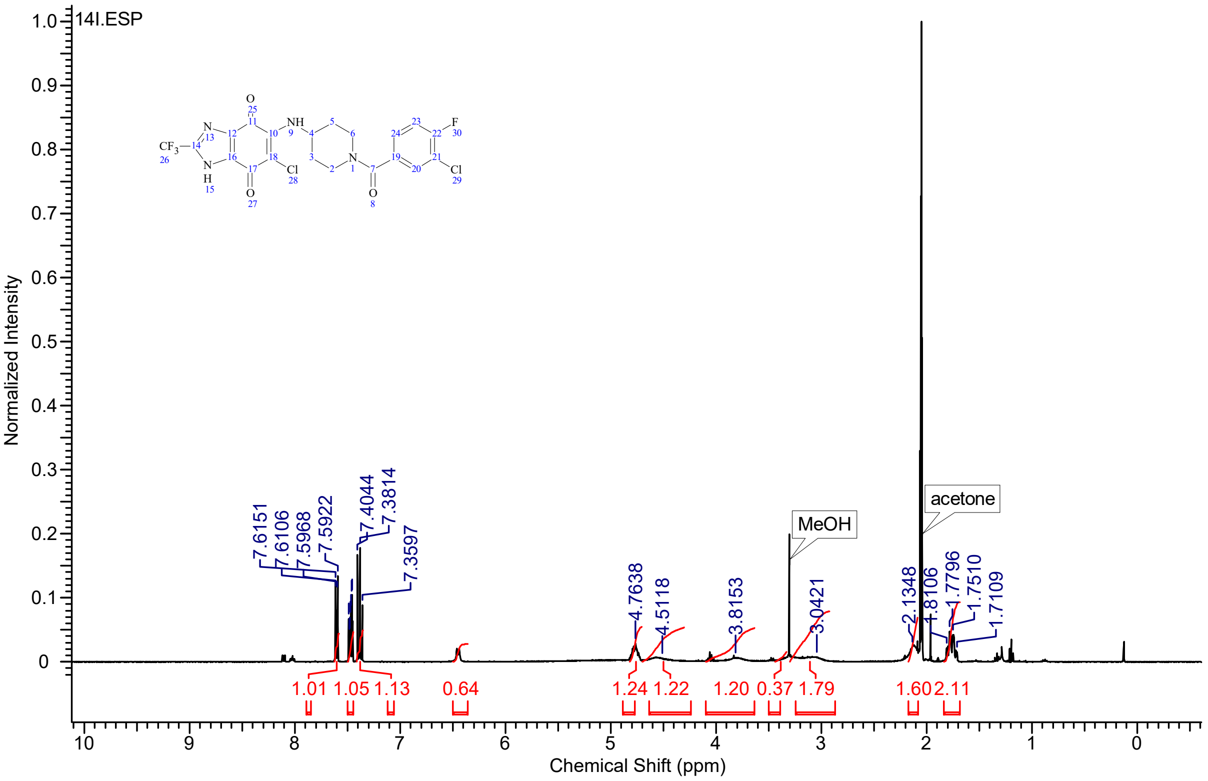

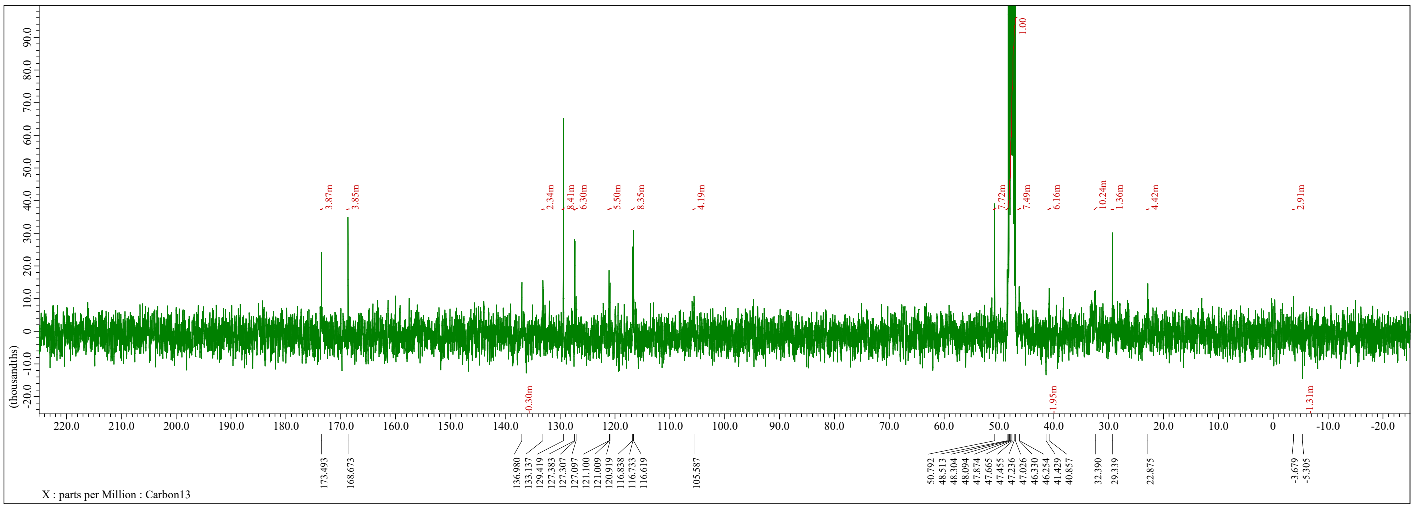

Figure S41. 1D 1H and 13C NMR spectrum of compound 14i

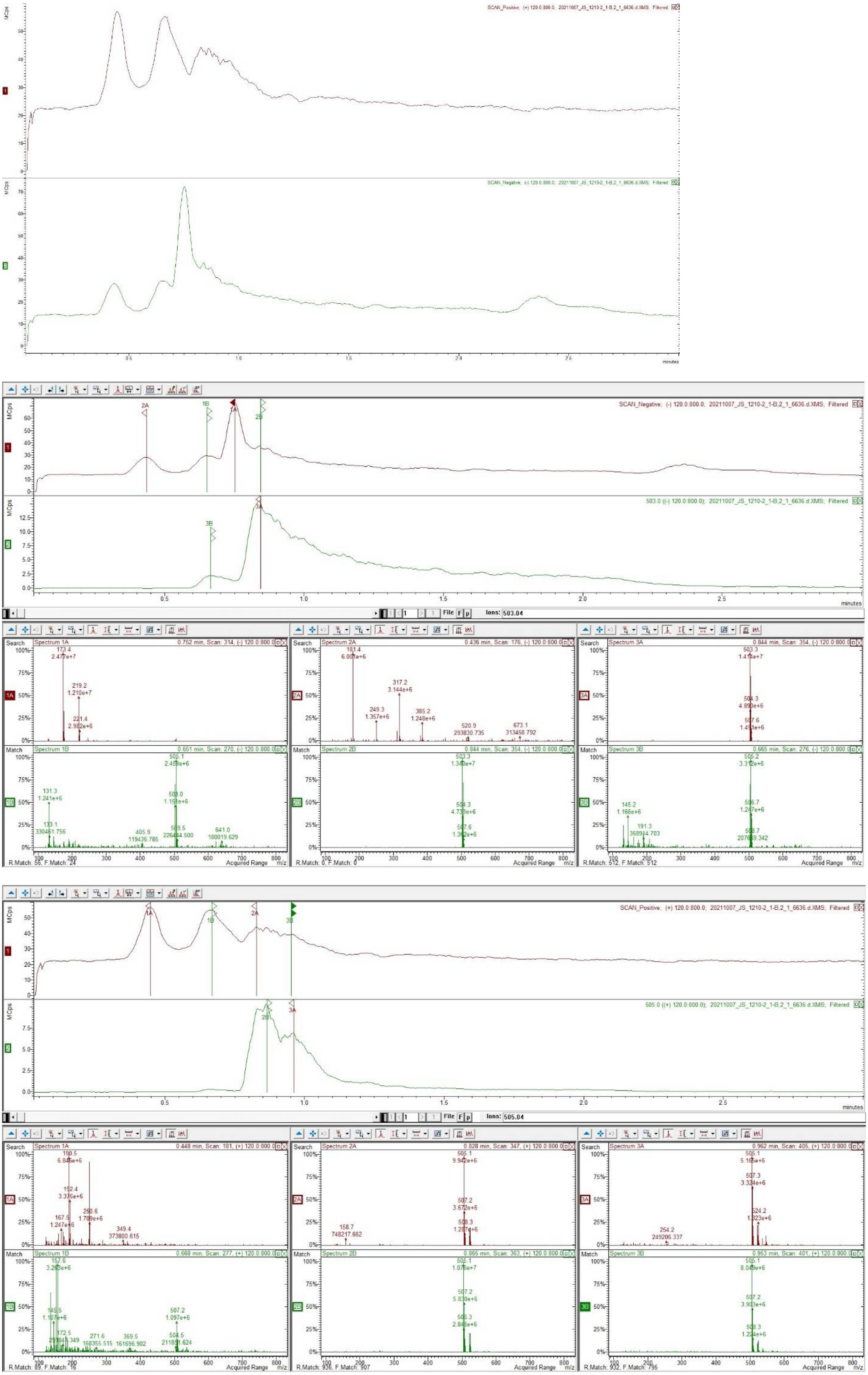

Figure S42. LC/MS spectrum of compound 14i

4-(4-((6-chloro-4,7-dioxo-2-(trifluoromethyl)-4,7-dihydro-1H-benzo[d]imidazol-5-yl)amino)piperidine-1-carbonyl)benzonitrile (14j) Following the general procedure for the synthesis of **14a-k**, the substitution reaction of **9b** with **13j** afforded **14j**. purple powder. **14j** fully dissolved in 600 μL of the methanol-d4 and a drop of acetone. Yield 70%; ^{1}H NMR (400 MHz, METHANOL-d4) δ ppm 1.62 - 1.76 (m, 2 H) 2.00 - 2.07 (m, 1 H) 2.20 (d, J=10.99 Hz, 1 H) 3.06 (br. s., 1 H) 3.22 - 3.30 (m, 1 H) 3.66 (d, J=11.45 Hz, 1 H) 4.65 (d, J=12.82 Hz, 1 H) 4.78 (s, 1 H) 7.62 (d, J=8.70 Hz, 2 H) 7.86 (d, J=8.24 Hz, 2 H); ^{13}C NMR (100 MHz, METHANOL-d4) δ 173.55, 169.09, 143.89, 143.00, 142.10, 140.21, 137.57, 132.43, 129.39, 127.46, 117.72, 113.36, 50.66, 46.07, 40.65, 2 carbon peaks in 173.55 ppm overlapped and low peak signal in 142.10 ppm; LC/MS (ESI, m/z) 476.6 $[M - H]^-$ 478.3 $[M + H]^+$.

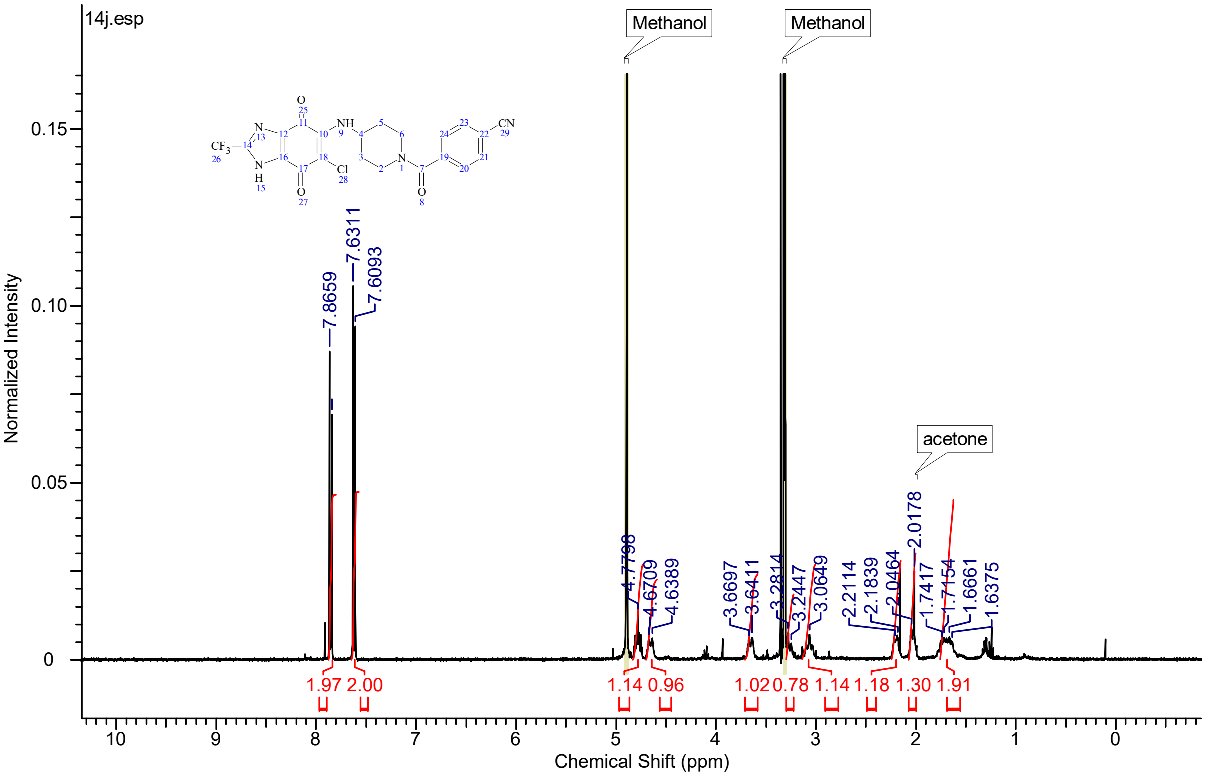

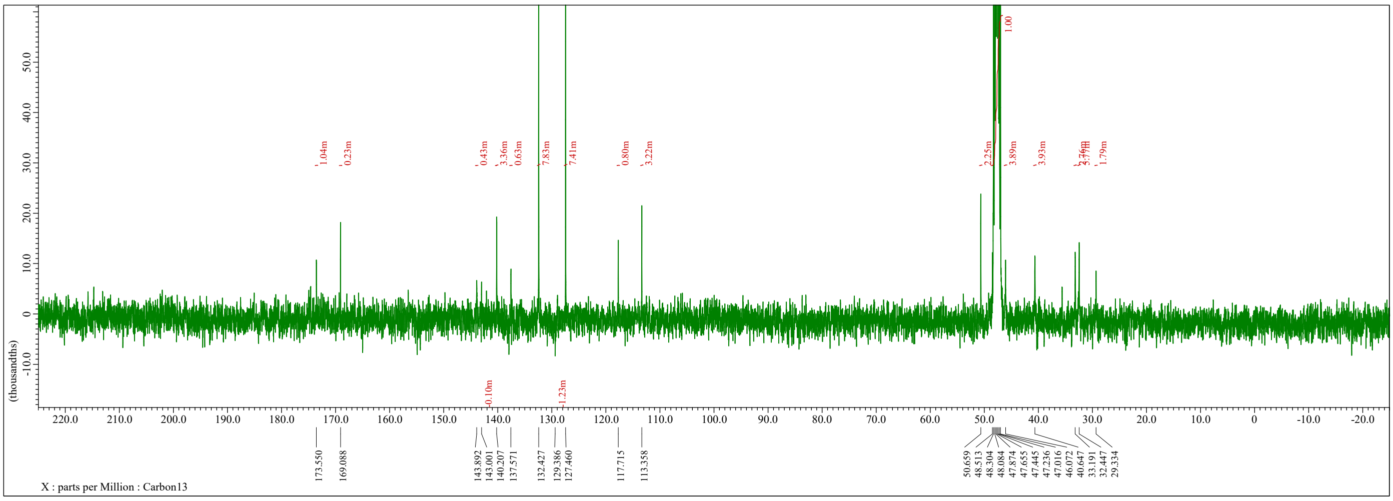

Figure S43. 1D 1H and 13C NMR spectrum of compound 14j

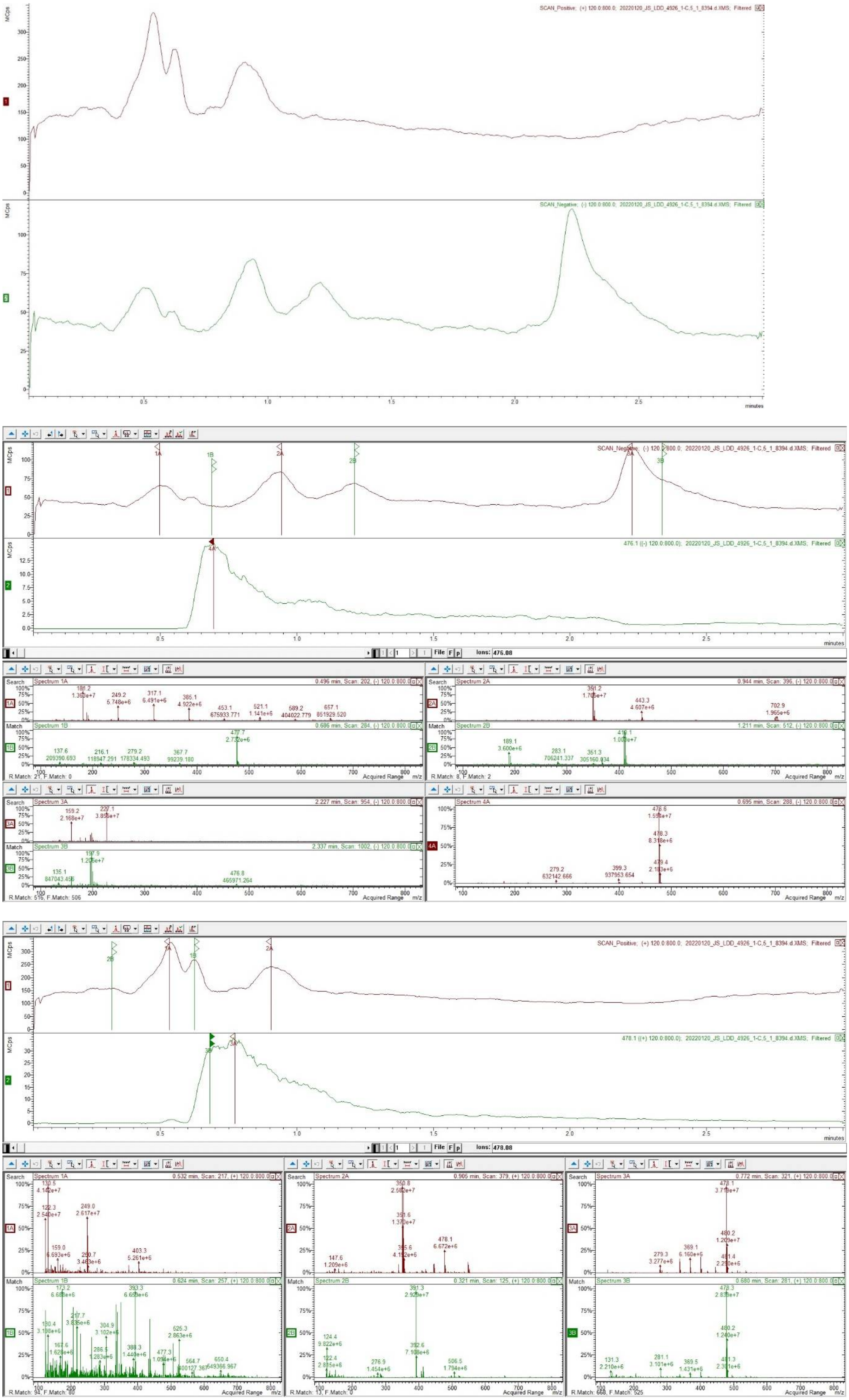

Figure S44. LC/MS spectrum of compound 14j

6-chloro-2-(trifluoromethyl)-5-((1-(4-(trifluoromethyl)benzoyl)piperidin-4-yl)amino)-1H-benzo[d]imidazole-4,7-dione (14k) Following the general procedure for the synthesis of **14a-k**, the substitution reaction of **9b** with **13k** afforded **14k**. purple powder. **14k** fully dissolved in 600 µL of the methanol-d4 and a drop of acetone. Yield 72%; ^{1}H NMR (400 MHz, METHANOL-d4) δ ppm 1.64 (d, J=6.87 Hz, 1 H) 1.72 (d, J=11.45 Hz, 1 H) 2.04 (br. s., 2 H) 2.21 (d, J=6.87 Hz, 1 H) 2.99 - 3.15 (m, 1 H) 3.15 - 3.29 (m, 1 H) 3.67 (d, J=12.37 Hz, 1 H) 4.65 (d, J=14.20 Hz, 1 H) 4.76 (s, 1 H) 7.64 (m, J=7.79 Hz, 2 H) 7.79 (m, J=8.24 Hz, 2 H); ^{13}C NMR (100 MHz, METHANOL-d4) δ 173.51, 169.56, 143.96, 139.61, 136.98, 131.58, 131.25, 127.26, 125.48, 125.44, 50.81, 46.14, 40.66, 2 carbon peaks in 173.51 ppm overlapped and 2 carbon peaks low signal in 140~145 ppm; LC/MS (ESI, m/z) 519.3 $[M - H]^-$ 521.4 $[M + H]^+$.

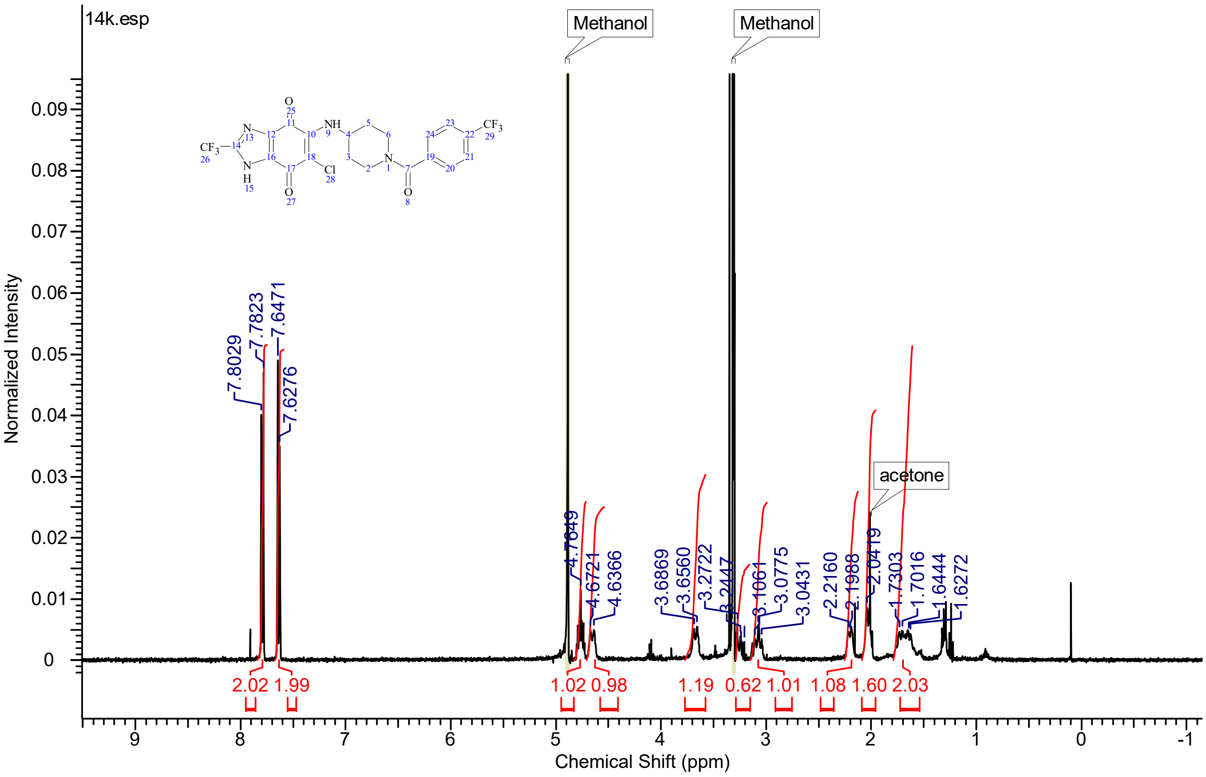

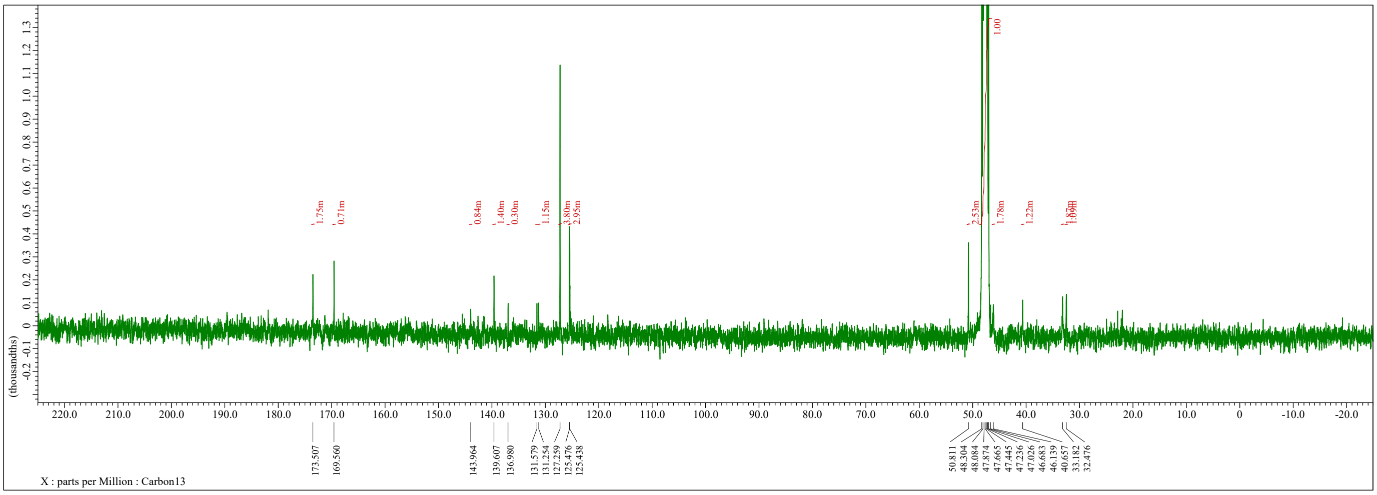

Figure S45. 1D 1H and 13C NMR spectrum of compound 14k

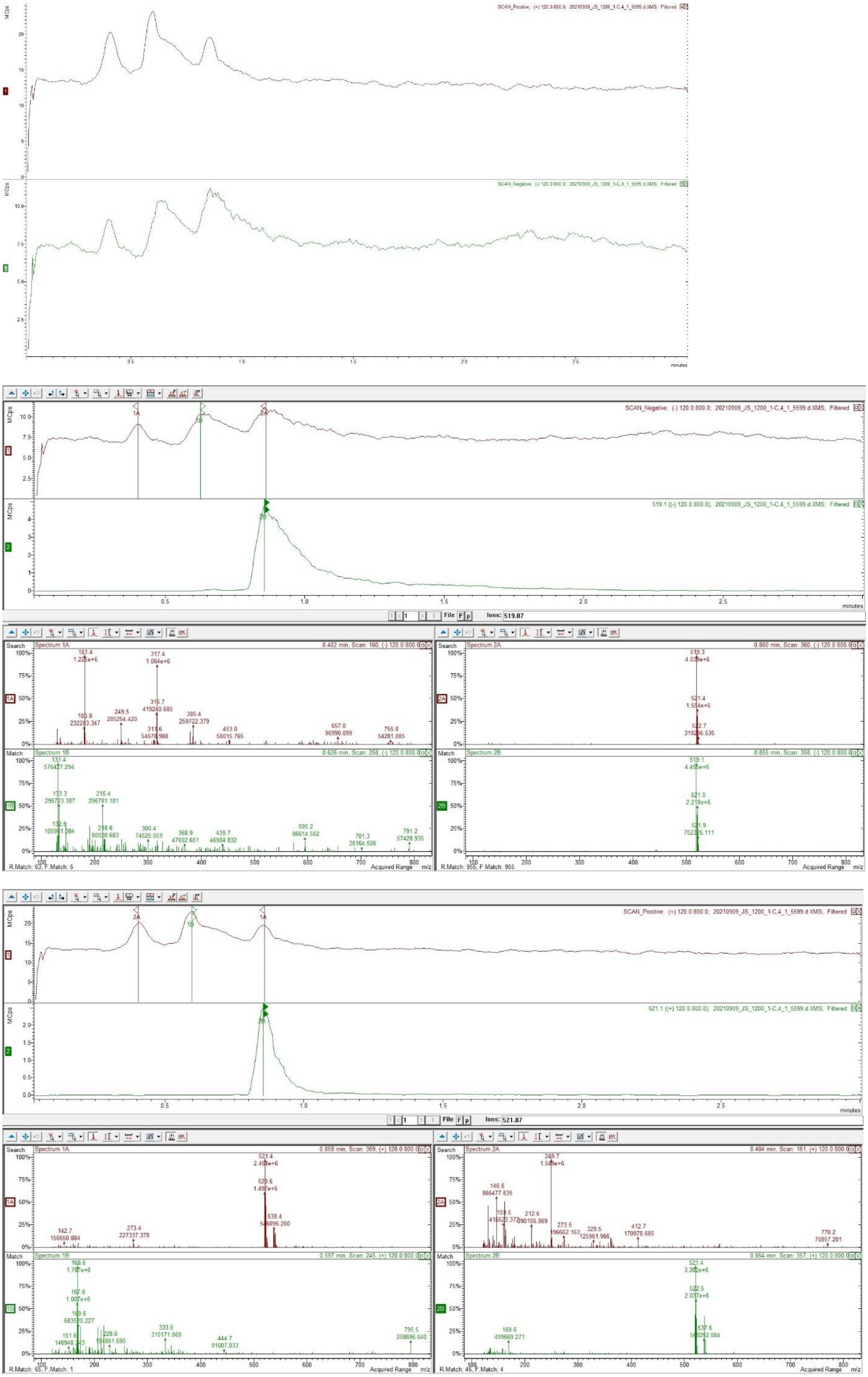

Figure S46. LC/MS spectrum of compound 14k

Reference

1. Khakh, B. S., and North, R. A. (2006) P2X receptors as cellsurface ATP sensors in health and disease. Nature 442, 527−532.
2. Evans, R. J., Derkach, V., and Surprenant, A. (1992) ATP mediates fast synaptic transmission in mammalian neurons. Nature 357, 503−505.
3. Surprenant, A., and North, R. A. (2009) Signaling at Purinergic P2X Receptors. Annu. Rev. Physiol. 71, 333−359.
4. Hattori, M., and Gouaux, E. (2012) Molecular mechanism of ATP binding and ion channel activation in P2X receptors. Nature 485, 207−212.
5. North, R. A. (2002) Molecular physiology of P2X receptors. Physiol. Rev. 82, 1013−67.
6. Kaczmarek-Hajek, K., Lorinczi, E., Hausmann, R., and Nicke, A. (2012) Molecular and functional properties of P2X receptors–recent progress and persisting challenges. Purinergic Signalling 8, 375−417.
7. Dal Ben, D., Buccioni, M., Lambertucci, C., Marucci, G., Thomas, A., and Volpini, R. (2015) Purinergic P2X receptors: Structural models and analysis of ligand-target interaction. Eur. J. Med. Chem. 89, 561− 580.
8. Dunn, P. M., Zhong, Y., and Burnstock, G. (2001) P2X receptors in peripheral neurons. Prog. Neurobiol. 65, 107−134.
9. Bradbury, E. J., Burnstock, G., and McMahon, S. B. (1998) The expression of P2X3 purinoreceptors in sensory neurons: effects of axotomy and glial-derived neurotrophic factor. Mol. Cell. Neurosci. 12, 256−268.
10. Honore, P., Kage, K., Mikusa, J., Watt, A. T., Johnston, J. F., Wyatt, J. R., Faltynek, C. R., Jarvis, M. F., and Lynch, K. (2002) Analgesic profile of intrathecal P2X3 antisense oligonucleotide treatment in chronic inflammatory and neuropathic pain states in rats. Pain 99,11−19.
11. Dorn, G., Patel, S., Wotherspoon, G., Hemmings-Mieszczak, M., Barclay, J., Natt, F. J. C., Martin, P., Bevan, S., Fox, A., Ganju, P., Wishart, W., and Hall, J. (2004) siRNA relieves chronic neuropathic pain. Nucleic Acids Res. 32, e49.
12. Barclay, J., Patel, S., Dorn, G., Wotherspoon, G., Moffatt, S., Eunson, L., Abdel'al, S., Natt, F., Hall, J., Winter, J., Bevan, S., Wishart, W., Fox, A., and Ganju, P. (2002) Functional downregulation of P2X3 receptor subunit in rat sensory neurons reveals a significant role in chronic neuropathic and inflammatory pain. J. Neurosci. 22, 8139− 8147.
13. Honore, P., Mikusa, J., Bianchi, B., McDonald, H., Cartmell, J., Faltynek, C., and Jarvis, M. F. (2002) TNP-ATP, a potent P2X3 receptor antagonist, blocks acetic acid-induced abdominal constriction in mice: Comparison with reference analgesics. Pain 96,99−105.
14. Young-Hwan Jung, Yong-Chul Kim, Discovery of Potent Antiallodynic Agents for Neuropahtic Pain Targeting P2X3 Receptors, ACS Chem. Neurosci., 2017, 8, 1465-1478.
15. Jaclyn Smith, Michael Kitt, Alyn Morice, Surinder Birring, Lorcan McGarvey, Mandel Sher, Anthony Ford, Inhibition of P2X3 by MK-7264 reduces 24-hour cough frequency in a randomized, controlled, Phase 2b clinical trial, European Respiratory Journal 2017 50: OA2932; DOI: 10.1183/1393003.congress-2017.OA2932.
16. Hedvig Bölcskei, Bence Farkas, P2X3 and P2X2/3 receptor antagonists, Pharmaceutical Pantent Analyst, Vol 3, no.1, Dec 2013.
17. Sung-Yu Hong, Kwang-Hoe Chung, Chung-Kyu Ryu, Synthesis and biological evaluation of benzimidazole-4,7-diones that inhibit vascular smooth muscle cell proliferation, Bioorganic & Medicinal Chemistry Letters, 14 (2004), 3563-3566.
18. Kwang-Hoe Chung, Sung-Yu Hong, Hea-Jung You, Rae-Eun Park, Chung-Kyu Ryu, Synthesis and biological evaluation of 5-arylamino-1H-benzo[d]imidazole-4,7-diones as inhibitor of endothelial cell proliferation, Bioorganic & Medicinal Chemistry, 14 (2006), 5795-5801.
